# Alkali‐Metal Base Catalyzed Electrocyclization of Isoprene Derivatives

**DOI:** 10.1002/chem.71057

**Published:** 2026-05-03

**Authors:** Mikaël Le Roch, Krzysztof Piech, Aurélien Crochet, Andreu Tortajada

**Affiliations:** ^1^ Department of Chemistry University of Fribourg Fribourg Switzerland

**Keywords:** alkali‐metal, biofuels, catalysis, cyclization

## Abstract

The catalytic electrocyclization of heptatrienes represents an attractive strategy to access seven‐membered carbocycles from acyclic precursors, whose medium‐size cyclic saturated hydrocarbon counterparts display physicochemical properties of interest for jet‐fuel applications. Here we uncover the potential of alkali‐metal amides to enable an efficient, high‐yielding and multigram‐scale electrocyclization of biobased ocimene and related trienes derived from isoprene. Experimental results show that both the nature of alkali metal (Li vs Na) and coordination by PMDETA (*N,N,N′,N″,N″*‐pentamethyldiethylenetriamine) play a decisive role in enabling efficient turnovers. Trapping and structural authentication of key metalated intermediates, together with DFT calculations, provide valuable mechanistic insights into the cyclization pathway and the factors governing reactivity. The transformation can proceed catalytically at relatively low loadings in neat conditions, with lithium‐based catalysis providing selectively 1,1,4‐trimethylcycloheptadienes albeit at higher catalyst loadings of 10 mol%, whereas sodium‐based systems can operate at lower loadings of 2 mol% and can promote the isomerization/cyclization of other trienes.

## Graphical abstract

Simple and abundant alkali‐metal base catalysis in combination with Lewis basic polyamines enables the electrocyclization of isoprene‐derived trienes to cycloheptadienes under neat conditions. Mechanistic insight supported by DFT guides a scalable route to seven‐membered carbocycles which, after reduction, give saturated cycloalkanes relevant as candidate jet‐fuel blendstocks.

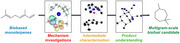

## 1 Introduction

Alkali‐metal organometallic bases, particularly those derived from lithium and sodium, play a central role in modern chemical synthesis due to their exceptional basicity, nucleophilicity, and overall reactivity profile [1, 2, 3, 4]. Alkali‐metal amides, alkoxides and alkyl reagents have enabled rapid and/or selective metalation, functional group manipulation, and bond‐forming transformations that often are inaccessible to weaker bases [5]. Despite their broad utility, one of the main limitations of their use is the need of (over)stoichiometric amounts of base to carry out the desired transformation, with consequent cost and low atom economy. Trying to solve this problem, in recent years a series of efficient transformations using catalytic amounts of alkali‐metal bases have been reported, showing the underdeveloped potential of these reagents in organic synthesis. For example, alkali‐metal bases and superbases have been employed in a range of catalytic transformations, from the allylation of imines using NaHMDS (HMDS = hexamethyldisilazide) [6] or superbasic LiTMP/KO*t*Bu mixtures (TMP = 2,2′,6,6′‐tetramethylpiperidide) [7] to olefin hydroamination [8, 9, 10], hydrophosphination of C═C bonds [11, 12], C─H bond silylation [13, 14, 15, 16, 17, 18, 19], catalytic hydride transfer [20, 21], and even isotopic exchange of aromatic substrates [22, 23, 24, 25, 26]. More recently the borylation of (hetero)aromatic substrates [27] or the isomerization of alkenes have been also achieved catalytically [28, 29], showcasing the diversity of transformations to which this strategy can be applied. Bringing forward this research field and advancing the state‐of‐the‐art of catalytic reactions mediated by alkali‐metals, here we tackled another challenging reaction, the electrocyclization of heptatrienyl anions to form 7‐membered ring carbocycles. This medium‐size carbocycle is difficult to form from neutral cycloaddition reactions, where typically Diels‐Alder favours the six‐membered ring product ([4+2]), and certain transition metal catalysts can promote the formation of eight‐membered rings ([4+4]) [30, 31, 32].

**FIGURE 1:**
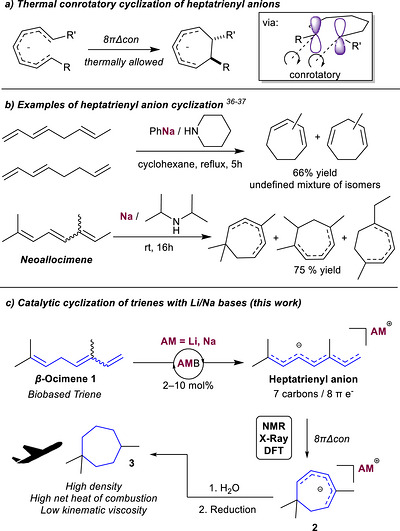
Electrocyclization of heptatrienyl anions.

Traditional synthesis of seven‐membered carbocycles requires the cleavage of bicyclic intermediates, ring closing metathesis or cyclization catalyzed by transition metals [33, 34]. However, the electrocyclization of heptatrienyl anions, an eight electron/seven atom thermally allowed reaction according to the Woodward–Hoffmann rules of pericyclic reactions (Figure 1a) [35], can deliver 7‐membered rings in a straightforward way, but it has been largely underutilized (Figure 1a). The process is thermodynamically driven by the formation of a C─C σ bond at the expense of a C─C π double bond and results in the formation of a cycloheptadiene with a conrotatory closing mechanism.

The first electrocyclizations using in situ–generated alkali‐metal amides (from the metal and an amine solvent) were reported independently by Kergomard [36], Kleinschmidt [37], Kloozterziel [38], and Bates [39] in the late 1960s, demonstrating that such strong bases can promote formation of seven‐membered rings. However, their advances were limited by poorly defined mixtures of isomers and competing polymerization pathways (Figure 1b). Since then, little progress has been made, as highlighted in a 2023 review by Orellana and Komijani [40], which underscored the challenges of this transformation, including the need of harsh conditions, (super)stoichiometric base loadings, and poor selectivity. Recent work by Orellana showcases the use of catalytic amounts of organic base DBU (1,8‐diazabicyclo[5.4.0]undec‐7‐ene) for the electrocyclization of heptatrienes, although this approach is limited to electron‐deficient trienes with relatively acidic C─H bonds [41].

Advancing alkali‐metal base–catalyzed transformations, here we report the LiTMP– and NaTMP–catalyzed electrocyclization of *β*‐ocimene **1** and related trienes under mild conditions (Figure 1c), supported by NMR monitoring, X‐ray crystallography of key intermediates, and computational analysis of the cyclization manifold. We target the cyclization of simple trienes/terpenes as sustainable aviation fuel blendstocks, since medium‐sized cycloalkanes can offer higher densities, higher heats of combustion, and often more suitable viscosities than their linear analogues [42]. Yet the absence of efficient routes to seven‐membered rings from readily available synthons has constrained access to cycloheptane derivatives for fuel applications, which are typically obtained only via harsh hydrogenation of strained terpenic bicyclic precursors under high temperature and high H_2_ pressure [43]. As a biorenewable, commercially available monoterpene (C_10_H_16_) accessible from plants (e.g., basil), *α*‐pinene thermolysis [44], or microbial fermentation [45, 46] *β*‐ocimene **1** features an alkene and a diene separated by a doubly allylic ─CH_2_─ unit that we expected to undergo preferential deprotonation to give a heptatrienyl anion, which can cyclize to form the seven‐membered scaffold **2** (Figure 1c). Subsequent hydrolysis and reduction would furnish cycloalkane **3**, a precursor identified by the Harvey group as having favourable physicochemical properties for jet‐fuel applications [43].

## 2 Results and Discussion

### 2.1 Stoichiometric Cyclization of *β*‐Ocimene

**FIGURE 2:**
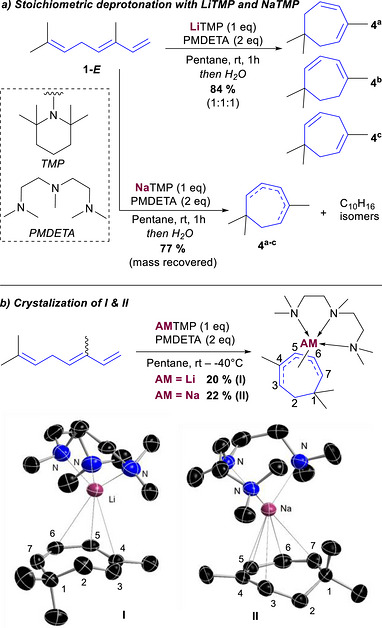
Stoichiometric deprotonation of **1‐*E*** (Top). XRD structure of metalated products **I** & **II**, ellipsoids are displayed at 30% probability and disorder/H atoms have been omitted for clarity (Bottom).

Inspired by the seminal work using in situ formed alkali‐metal amides [36, 37, 38, 39] we started our investigation by treating *E*‐*β*‐ocimene **1‐*E*** with stoichiometric amounts LiTMP or NaTMP in the presence of two equivalents of the tridentate Lewis donor PMDETA in pentane, to ensure solubility of the metalated intermediates. Indeed, the AMTMP/PMDETA combination has been shown experimentally and by DFT calculations, to be a highly reactive and non‐nucleophilic base in solution [22, 28, 47]. In both cases, the complete conversion of starting material was achieved within an hour of reaction, but they gave different outcomes. Using LiTMP, we observed the formation of three products arising from the expected electrocyclization of **1‐*E*** in 84% yield (Figure 2). Those products have been isolated by semi‐preparative GC and their structure elucidated by NMR spectroscopy (^1^H COSY, HMBC, HSQC). They consist of three different isomers with a seven‐membered ring scaffold bearing 1,1,4‐methyl substituents and which would result from the direct protonation of anion **2** (Figure 1), exhibiting distinct position of the double bonds.

They were found in a 1:1:1 ratio (determined by ^1^H NMR spectroscopy), where compounds **4^a^** and **4^b^** are conjugated dienes, whereas **4^c^** is a skipped diene. On the other hand, NaTMP gave only partially the **4^a–c^** isomers along with other species that could be analysed by GC‐MS, all sharing the same m/z ratio than the starting material, indicating that all those molecules are C_10_H_16_ isomers. Despite extensive attempts, semi‐preparative GC did not allow their isolation and structural characterization. Under Na‐mediated conditions, the **4^a–c^** species appear to be minor components relative to the other C_10_H_16_ isomers.

To gain further information about the metalation of ocimene, crystallization experiments using LiTMP and NaTMP enabled the isolation of cyclic metalated intermediates in moderate yields, and their structures could be determined by X‐ray single crystal diffraction (Figure 2b). Monomeric structures of metalated species were found, both featuring a M–π biallylic interaction motif. For the Li‐metalated intermediate **I** (PMDETA·LiC_10_H_15_), the cation is positioned between the π‐system of the seven‐membered ring and the PMDETA ligand. The shortest Li─C_4_ contact is 2.256(10) Å, with a Li─C_5_─C_1_ angle of 109.8(3)°, indicating that the cation is not fully centred above the seven‐membered ring. The ring displays a delocalized geometry that can be described as a partial skipped diene, with shorter C_3_─C_4_ and C_6_─C_7_ bonds (1.351(8) and 1.335(8) Å, respectively) compared to the longer C_4_─C_5_ and C_5_─C_6_ bonds (1.435(8) and 1.401(7) Å). Those values are coherent with similar lithium pentadienyl anions crystallized in the literature, also showing similar skipped diene conformation [48].

For the Na‐metalated intermediate **II** (PMDETA·NaC_10_H_15_), two molecules crystallized within the unit cell with an average of Na−C_5_ length of 2.67(3) Å and a Na─C5─C1 angle of 70.5(6)°, indicating a more centred cation on the seven‐membered ring and a bigger delocalization pattern, as evidenced by the more homogeneous C─C bond lengths of 1.35(3), 1.39(3), 1.40(15), and 1.37(2) Å for the C_3_─C_4_─C_5_─C_6_─C_7_ motif. In addition, the sodium centre engages in five Na···C π‐contacts in **II**, compared with three Li···C in **I**.

The Li‐ and Na‐metalated species were studied in C_6_D_6_ solution by ^1^H DOSY NMR spectroscopy. Crystals of **I** and **II** were soluble in C_6_D_6_, and their apparent molecular weights in solution, determined using the Stalke calibration curves [49], were 327 g·mol^−^^1^ (4% error) for **I** and 316 g·mol^−^^1^ (5% error) for **II**. These results indicate that complexes **I** and **II** are monomeric in solution, consistent with their solid‐state structures determined by X‐ray diffraction.

**FIGURE 3:**
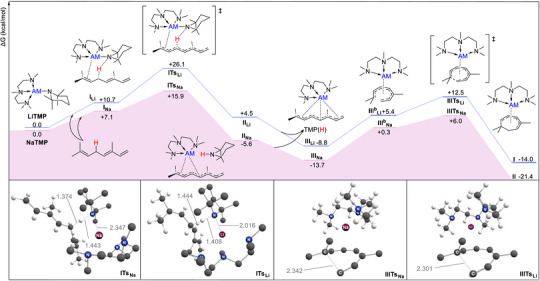
Lowest Gibbs energy profile of electrocyclization of *β*‐ocimene **1** calculated in gas phase at T = 298.15K and p = 1atm where AM = Li (blue path) or Na (pink path) (kcal/mol). Structures of TS are displayed with relevant bond length in Å, with some H omitted for clarity. Images have been generated using Chemcraft software.

We then conducted density functional theory (DFT) calculations at the ωB97X‐D3/def2‐TZVP [50, 51, 52] level of theory to rationalize the mechanism of cyclization (Figure 3). The monomeric LiTMP and NaTMP species coordinated by PMDETA were selected, as the corresponding complexes [(PMDETA)AM(TMP)] (AM = Li or Na) represent one of the most stable aggregation states in solution [47]. With Li, the first step involves the approach of LiTMP (alkali metal amide) with ocimene to form the **I_Li_** adduct. In this complex, a proton from the −CH_2_− group is positioned toward the nitrogen of TMP, which facilitates deprotonation through the **ITS_Li_** transition state. This step has an overall energy barrier of +26.1 kcal·mol^−^^1^, leading to the formation of the linear heptatrienyl anion **II_Li_**. After decoordination of TMP(H), forming **III_Li_**, an isomerization from this linear structure to the twisted **III^b^_Li_** is necessary and happens with a cost of +14.2 kcal·mol^−^^1^, probably due to the reduced delocalization and steric hindrance caused by this deformation. Nevertheless, this structure is perfectly set to allow the electrocyclization through the conrotatory transition state **IIITS_Li_**, for an overall +21.3 kcal·mol^−^^1^ barrier, leading to the metalated cyclized product **I**.

For Na, the reaction proceeds through a similar pathway, with a reaction globally more exergonic than in the case with Li, involving a first deprotonation by NaTMP through a +15.9 kcal·mol^−^^1^ barrier, along with an isomerization to the twisted structure to form **III^b^_Na_**, and the same cyclization process allowing the formation of **II** with an overall barrier of +19.7 kcal·mol^−^^1^.

**FIGURE 4:**
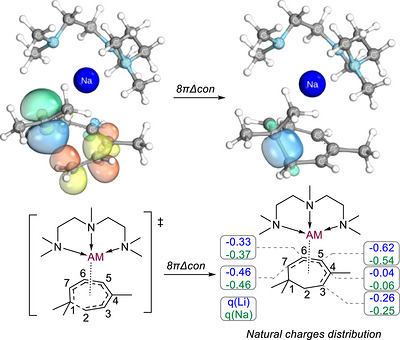
Intrinsic atomic orbitals interaction responsible for electrocyclization for AM = Na (Top). Natural charges distribution along the pentadienyl anion with AM = Li and Na (Bottom). Images have been generated using IBOView software [54].

Natural Bonding Orbitals (NBO) [53] and Intrinsic Atomic Orbitals (IAO) [54] analyses provide an orbital insight of the cyclization (Figure 4). In the cyclization transition state with Li and Na (**IIITs**) the negative charge is mainly located in a π‐type orbital on the terminal carbon C_1_. During ring closure, second‐order perturbation analysis indicates donation from the C_1_‐centered π‐type lone pair into the π* antibonding orbital of the C_2_─C_3_ double bond, resulting in a delocalized three‐centre, four‐electron interaction (C_1_─C_2_─C_3_) that promotes formation of the new C_1_─C_2_ σ bond in **I** and **II**. As a result, the negative charge becomes delocalized over the remaining C_3_─C_7_ fragment. In the Li‐metalated cyclized product **I**, the more localized nature of the charge is evidenced by the −0.62 natural charge on C_5_, compared to −0.54 in **II**, as well as by the presence of a more populated lone pair on C_5_ (1.44 e^−^ in **I** versus 1.38 e^−^ in **II**). These features agree with the more pronounced “skipped diene” character observed in the X‐ray structures for the lithium‐metalated specie.

### 2.2 Moving Toward Catalytic Regimes

Although substoichiometric amounts of alkali‐metal bases were employed in the late 1960s [36, 37, 38, 39], the absence of structural elucidation of the products, blurred identity of organometallic species involved and limited mechanistic understanding rendered these reactions of limited practical utility. With the generation of biofuel precursors in mind, we envisioned shifting toward a catalytic and selective electrocyclization in neat conditions. Because of the high amounts needed for a neat reaction, commercial Z‐*β*‐ocimene **1‐*Z*** (75% pure, contaminated with limonene) has been used as optimization starting material. Based on the selectivity observed with stoichiometric LiTMP cyclization, we first started our investigation by adding 10 mol% of LiTMP along with 10 mol% PMDETA at room temperature for 2 h, allowing the formation of neoalloocimene **1’** as an isomerized product arising from **1** (6:1 ratio for the *E*:*E* isomer against *E:Z*) and 34% of cyclized product (Table 1, **entry 1**). The reaction was then allowed to proceed for 16 h, and we were pleased to observe complete conversion of ocimene, affording 64% of the cyclized conjugated dienes **4^a^** and **4^b^** in a quasi 1:1 ratio, along with traces of the skipped diene **4^c^**, consistent with the higher thermodynamic stability of the conjugated dienes (**entry 2**). Traces of other isomers and dimeric products could also be observed by GC‐MS. Slightly heating the reaction to 40°C allowed the yield to reach 70% (**entry 3**). We then tried to reduce the amount of LiTMP and donor to 5 mol% and still observed an acceptable yield of 53% (**entry 4**).

**TABLE 1:** Optimization of catalytic electrocyclization of *β*‐ocimene **1**.

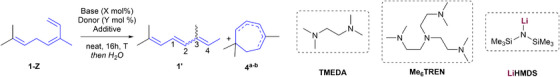
Entry	Base (X mol%)	Donor (Y mol%)	T (°C)	1’ (%)^a^	4^a^+4^b^ (%)^a^
1^b^	LiTMP (10)	PMDETA (10)	r.t.	28% (6:1)^c^	34%
2	LiTMP (10)	PMDETA (10)	r.t.	—	64%
3	LiTMP (10)	PMDETA (10)	40	—	70%
4	LiTMP (5)	PMDETA (5)	40	—	53%
5	LiTMP (10)	TMEDA (10)	40	20% (33:1)^c^	25%
6	LiTMP (10)	Me_6_TREN (10)	40	71% (3:1)^c^	—
7^d^	LiTMP (10)	PMDETA (10)	40	—	61%^e^
8^f^	LiTMP (10)	PMDETA (10)	40	—	71%
9	LiHMDS (10)	PMDETA (10)	40	< 5%	—
10	LiCH_2_SiMe_3_ (10)	PMDETA (10)	40	—	32%
11	**I** (10)	—	40	—	53%
12^b^	NaTMP (10)	PMDETA (10)	r.t.	—	∼20%^g^
13	NaTMP (2)	PMDETA (5)	r.t.	—	∼20%^g^

^a^ NMR yield determined using 1,3,5‐Trimethoxybenzene as internal standard.

^b^ 2 h of reaction.

^c^ d.r ratio between the *E*(C_1_ = C_2_)/*E*(C_3_ = C_4_) isomer and the *E*(C_1_ = C_2_)/*Z*(C_3_ = C_4_) isomer determined by ^1^H NMR spectroscopy.

^d^ Using **1‐*E*** (> 95% purity).

^e^ Isolated yield.

^f^ A 1:1 mixture of **1‐*E*** and **1‐*Z***, containing 13% limonene, was used.

^g^ Only a part of known cyclized product could be detected, alongside with other cyclized isomers (see Figure 7).

To further assess the role of the Lewis base donor, we modified the lithium coordination sphere by replacing the tridentate ligand PMDETA with the bidentate *N,N,N',N'*‐tetramethylethylenediamine (TMEDA) (**entry 5**). Under these conditions, the reaction proceeded significantly more slowly, leading primarily to isomerization of ocimene into neoalloocimene (4:1), with only 25% yield of the cyclized product. In contrast, using the tetradentate ligand tris[2‐(dimethylamino)ethyl]amine (Me_6_TREN) completely suppressed formation of the electrocyclization product, leading exclusively to full conversion to neoalloocimene within 16 h (**entry 6**). This highlights that the reaction outcome can be tuned depending on the conditions, particularly the choice of Lewis base donor, allowing electrocyclization to be avoided. Taken together, these latter examples showcase the unique effectiveness of PMDETA, which promotes faster reactivity and enables access to the electrocyclization pathway.

Then, the role of limonene impurity in the catalytic cycle has been discarded, as the reaction has been performed with the purified **1‐*E*** following our best conditions (**entry 7**) and allowed the formation and isolation of 61% cyclized product. Similar results were also obtained when a mixture of **1‐*E*** and **1‐*Z*** containing limonene were cyclized, obtaining 71% yield (**entry 8**). These results also showed the possibility of using independently *β*‐ocimene in its *E* or *Z* configuration, obtaining comparable yields of cyclized products.

To probe mechanistic features of this transformation, the weaker lithium amide LiHMDS was evaluated and showed no conversion of ocimene overnight under otherwise identical conditions, probably due to its lower basicity compared to LiTMP (**entry 9**).

**FIGURE 5:**
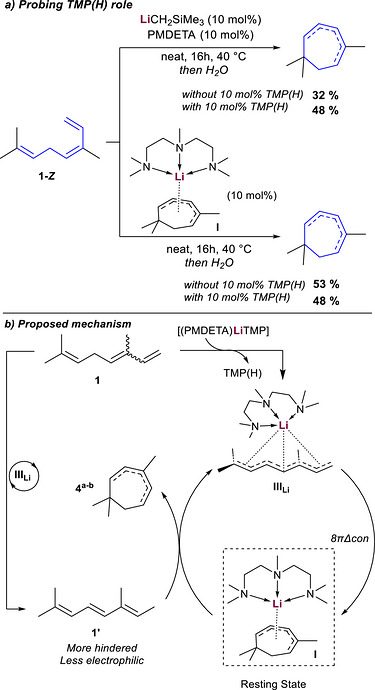
Alkyllithium electrocyclization of heptatrienyl anion (Top). Proposed mechanism for catalytic electrocyclization (Bottom).

In contrast, an alkyllithium reagent (LiCH_2_SiMe_3_) promoted cyclization, albeit in only 32% yield and with substantial oligomer formation (detected by GC and NMR) (**Entry 10** and Figure 5a). These results indicate that cyclization is not specific to TMP‐based amides, but that competing pathways can significantly lower the yield. In this case, oligomerization is attributed to nucleophilic addition of the alkyllithium to the ocimene diene. Catalysis was next carried out using the well‐defined metalated cycle **I** (PMDETA·LiC_10_H_15_) (**Entry 11** and Figure 5a). The seven‐membered rings were obtained in 53% yield, although oligomeric byproducts were still detected by NMR (Figure 5a). Unlike the LiCH_2_SiMe_3_ experiments, pre‐mixing of TMP(H) did not improve the result (48% yield, Figure 5a), consistent with **I** being unable to deprotonate TMP(H) and showing that dimerization/oligomerization can also occur in the absence of an alkyllithium reagent. In situ monitoring further revealed that ocimene **1** is not isomerized to neoalloocimene **1′** in the presence of **I** (Figure S28), whereas LiTMP catalysis leads to complete isomerization within 10 min. Neoalloocimene might be a slightly poorer electrophile than ocimene, due to the lack of a readily accessible terminal double bond, which would make nucleophilic addition slightly less competitive and deprotonation slightly more favourable, allowing the cyclization process to be faster than the nucleophilic attack which may account for the superior performance in the presence of LiTMP.

On this basis, we propose that **III_Li_**, formed upon deprotonation by LiTMP, cyclizes to the monomeric cyclometalated lithium species **I**, which is observed by ^1^H NMR monitoring during the reaction. Species **I** could then deprotonate neoalloocimene **1′** (generated catalytically from **1‐*E*** via isomerization promoted by **III_Li_**), thereby regenerating **III_Li_** and releasing **4^a–b^** (Figure 5b). This is consistent with TMP(H) not participating in turnover and with isolated **I** being a competent species for catalysis.

We explored this turnover step by DFT, finding that the calculations capture the qualitative trend observed experimentally, with deprotonation of **I** by TMP(H) disfavoured by +15.9 kcal·mol^−^^1^ relative to deprotonation by neoalloocimene. However, despite multiple attempts to compute the deprotonation in different conformations, or using solvation models, the computed activation barrier for the proton transfer in gas phase (ΔG^‡^ = + 31.0 kcal·mol^−^^1^; Figures S50–S54 for further details) is likely overestimated compared to the speed of ocimene **1** (or neoalloocimene **1’**) conversion observed experimentally (Figure 5a) and cannot be interpreted quantitatively. Under neat conditions, the alkali‐metal species (ion pairing and PMDETA/substrate coordination) can differ from the model used in the calculations, which may significantly affect the effective barrier. Overall, considering the DFT insights and the experimental observations, the proposed mechanism appears reasonable.

**FIGURE 6:**
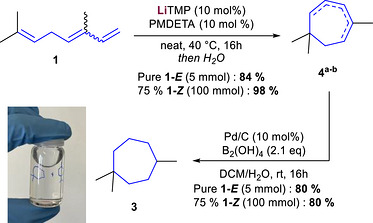
Selective cyclization and hydrogenation to form cycloalkane **3**.

To assess the scalability of this procedure, a 5 mmol catalytic electrocyclization was first performed using pure **1‐*E*** as starting material (Figure 6). This scaled‐up reaction afforded the cyclized product **4^a‐b^** in 84% isolated yield, which was subsequently hydrogenated using Pd/C and B_2_(OH)_4_ to give the desired 1,1,4‐trimethylcycloheptane **3** seven‐membered alkane ring in 80% isolated yield [55]. Further scale‐up was achieved by applying the same protocol to 100 mmol of commercially available **1‐*Z***, resulting in the formation of the limonene + seven‐membered ring mixture in 98% isolated yield. Subsequent hydrogenation provided a mixture of seven‐ and six‐membered aliphatic cycles in 80% yield, demonstrating the potential of this catalytic approach for the preparation of the cycloalkane **3** on a larger scale.

**FIGURE 7:**
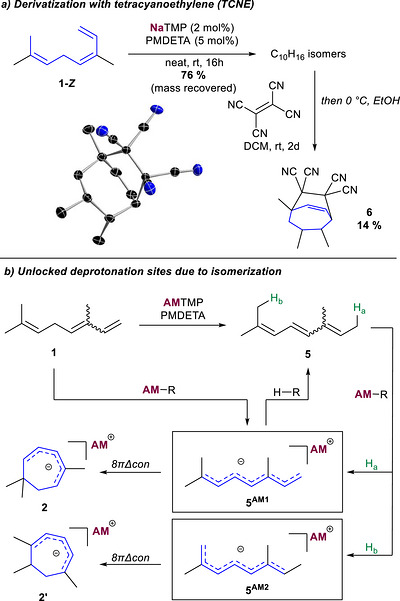
Derivatization of C_10_H_16_ isomers derived from NaTMP catalysis (XRD structure displayed at 50% probability and H atoms have been omitted for clarity) (Top). Proposed explanation for the formation of **2’** scaffolds under catalytic conditions (Bottom).

Then, sodium‐base catalysis was investigated (Table 1 **entry 12–13** and Figure 7). Using 10 mol% NaTMP in combination with 10 mol% PMDETA led to complete conversion of **1‐*Z*** within 120 min at room temperature, confirming the expected higher reactivity compared to LiTMP. However, similarly to the stoichiometric experiments, no selectivity was observed and a mixture of isomers was obtained. Interestingly, decreasing the catalyst loading to 2 mol% NaTMP and 5 mol% PMDETA still resulted in complete conversion overnight (16 h, 76% mass recovered). Derivatization of the crude mixture with tetracyanoethylene (TCNE) allowed us to crystallize a [4+2] adduct **6** in 14% crystalline yield over two steps, showing a 1,2,6‐trimethyl scaffold seven‐membered ring. This result demonstrates that lower amounts of NaTMP are sufficient to promote the catalytic process, which represents an advantage for large‐scale jet‐fuel precursor production, where high selectivity in cyclization is not required to achieve suitable physicochemical properties [56]. The formation of different seven‐membered rings suggests the presence of multiple isomers differing in methyl substitution patterns, as already deciphered by Kergomard with in situ generated sodium amide starting from neoalloocimene (Figure 1b) [36].

Although the isomerization of *β*‐ocimene **1** to neoalloocimene **1’** is observed by NMR and occurs under both lithium‐ and sodium‐catalyzed conditions, the difference in selectivity is proposed to arise from the distinct reactivity of the two alkali metal species rather than from the isomerization itself (Figure 7b). Upon formation of the fully conjugated heptatriene, two terminal deprotonation sites become accessible: deprotonation at *Ha* leads to the heptatrienyl anion **5^AM1^**, which cyclizes to the 1,1,4‐trimethyl seven‐membered ring **2**, whereas deprotonation at *Hb* generates the alternative heptatrienyl anion **5^AM2^**, responsible for the formation of the 1,3,6‐trimethyl isomer **2’**. Under sodium catalysis, the higher reactivity of the heavier alkali‐metal base allows deprotonation at both *Ha* and *Hb*. In contrast, under lithium catalysis, the lower reactivity of the alkali‐metal base is proposed to favour deprotonation at the most acidic, less crowded position (*Ha*), thereby restricting access only to the alternative intermediate **5^AM1^** and resulting in increased regioselectivity. The latter example shows that the reduced reactivity of Li‐catalyzed process can allow a more chemoselective transformation compared to its Na‐catalyzed equivalent. Overall, they add up to the recent examples of the divergent reactivity that can be obtained using different alkali‐metals [3, 4, 57, 58].

### 2.3 Catalytic Cyclization of Monoterpenes Derivatives

**FIGURE 8:**
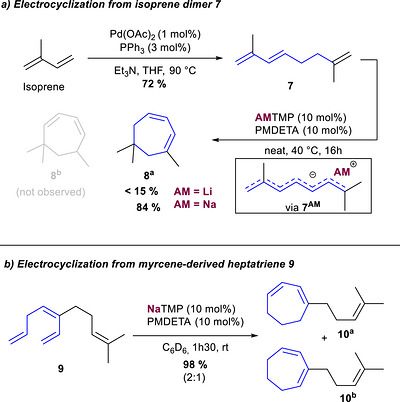
Catalytic electrocyclization of isoprene dimer **7** (Top) and myrcene derivative **9** (Bottom).

With the optimal conditions identified, isoprene was selected as substrate, as it is the fundamental building block of all monoterpenes (including ocimene) and the most abundant one. Early reports from the late 1960s described the dimerization of isoprene using in situ generated alkali‐metal amides [59, 60, 61, 62, 63, 64]; however, these systems generally suffered from low yields and poor selectivity due to competitive polymerization. The use of transition‐metal catalysis, in particular palladium complexes [65, 66], later enabled more selective isoprene dimerization into well‐defined monoterpene structure. For example, the Pd(OAc)_2_/PPh_3_ catalytic system can be used, affording **7** in 72% yield from isoprene (Figure 8a) [65].

Submitting neat **7** to 10 mol% LiTMP and PMDETA at 40°C did not allow electrocyclization to occur completely and led to 15% conversion. This can be explained by the fact that unlike *β*‐ocimene, **7** does not possess a double‐allylic −CH_2_− moiety that would allow direct formation of a heptatrienyl anion. Formation of **7^AM^** requires a preliminary isomerization step, followed by a second deprotonation. More reactive NaTMP, known for its enhanced efficiency for alkene isomerization reactions [28] was then used in the same conditions, leading to full conversion of **7** selectively into the cyclic product **8^a^** in 84% isolated yield. These results show that the different reactivity profiles of the Li and Na species can be exploited for the different substrates to finely tune the overall outcome of the cyclization reactions. For the electrocyclization of **7**, the exclusive formation of a single double‐bond positional isomer could arise from the symmetrical carbon framework of **7^AM^** and the higher thermodynamic stability of the more substituted trisubstituted double‐bond pattern in **8^a^**, allowing the formation of a single diastereoisomer in high yield starting from very abundant and economic isoprene. When other trienes were tested under catalytic conditions either with lithium or sodium bases, competitive polymerization was observed in the presence of a conjugated diene to a carbonyl group or aromatic ring, as well as if we started from an already conjugated linear triene, showing that the outcome of electrocyclization is highly substrate dependent (see SI, Figures S43–S48, for examples). However, the myrcene‐derived substrate **9** proved particularly well‐suited, undergoing smooth electrocyclization within 1.5 h at room temperature to give the corresponding product **10^a‐b^** in 98% isolated yield as a mixture of positional isomers, reflecting their comparable thermodynamic stability (Figure 8b).

## 3 Conclusion

In this work, we uncover the unique capacity of lithium‐ and sodium‐based reagents to induce the catalytic electrocyclization of biobased ocimene, to form the corresponding 7‐membered ring. Initial stoichiometric experiments with a lithium amide base in combination with the tridentate Lewis donor PMDETA confirmed the quantitative formation of a 7‐membered ring framework bearing a 1,1,4‐trimethyl substitution pattern as the major product, whereas the deprotonation using a more reactive sodium base afforded a mixture of isomeric 7‐membered rings with different substitution patterns. Single‐crystal X‐ray diffraction studies shed light on the key intermediates involved in the cyclization, adding to the scarce examples of non‐aromatic pentadienyl anions reported to date. In parallel, DFT calculations were carried out to elucidate the reaction mechanism, revealing an overall exergonic pathway leading to the metalated seven‐membered ring. The computations support a conrotatory thermal electrocyclization mechanism in agreement with Woodward–Hoffmann rules and confirm the structural trend observed by XRD, namely a more delocalized anion in the presence of the bulkier sodium counterion compared to lithium. The implementation of catalytic conditions enabled access to seven‐membered ring systems from ocimene in neat conditions on large scale, in which lithium‐based catalysis ensured selective formation of 1,1,4‐trimethylcycloheptadienes, albeit with reduced reactivity and higher catalyst loadings, whereas sodium‐based systems displayed higher reactivity at lower loadings but with lower selectivity. Overall, this methodology enables the formation of jet fuel blendstock candidates on a 100 mmol scale using mild and neat conditions, starting from ocimene or triene **7**, synthesized from isoprene, one of the most abundant renewable building blocks available.

## 4 Experimental Section

### 4.1 General Considerations

All manipulations were carried out under an argon atmosphere using standard Schlenk‐line and glovebox techniques. Pentane was degassed, purified through an MBraun SPS 5 system, and stored over 4 Å molecular sieves. C_6_D_6_ was dried over NaK, degassed by three freeze–pump–thaw cycles, and stored over 4 Å molecular sieves. Substrates were degassed prior to use, and polydentate amines were dried over CaH_2_, distilled under reduced pressure, and stored over molecular sieves. NMR spectra were recorded at room temperature, and DOSY experiments were performed in C_6_D_6_ using the external calibration curve method described by Stalke and coworkers [49].

### 4.2 Preparation of Organometallic Intermediates **I** and **II**

In a glovebox, an oven‐dried ampoule equipped with a magnetic stir bar was charged with pentane (5 mL), PMDETA (0.209 mL, 1.0 mmol, 2.0 equiv), and commercial *β*‐ocimene **1‐*Z*** (0.114 mL, 0.5 mmol, 1.0 equiv, 74.4% purity). Addition of LiTMP (73.6 mg, 0.5 mmol, 1.0 equiv), followed by heating at 40°C for 1 h, afforded after cannula filtration and storage at −40°C yellow crystals of **I** (32 mg, 20%) after washing with cold pentane. Under analogous conditions, replacement of LiTMP with NaTMP (81.6 mg, 0.5 mmol, 1.0 equiv) afforded black crystals of **II** (30 mg, 22%) after 1 h stirring and cooling to −40°C.

**I**: **^1^H NMR** (400 MHz, C_6_D_6_) δ 5.98 (dd, *J* = 10.3, 7.6 Hz, 1H), 4.75 (tdd, *J* = 6.4, 2.2, 1.0 Hz, 1H), 4.56 (d, *J* = 10.3 Hz, 1H), 3.46 (dd, *J* = 7.7, 2.3 Hz, 1H), 2.45 (d, *J* = 6.5 Hz, 2H), 2.17 (d, *J* = 1.0 Hz, 3H), 2.01 (s, 4H), 1.91 (s, 11H), 1.82 – 1.68 (m, 8H), 1.58 (s, 6H). **^13^C NMR** (101 MHz, C_6_D_6_) δ 141.6, 128.5, 107.1, 94.1, 71.3, 57.4, 54.1, 48.2, 45.7, 40.2, 34.5, 27.0. **Elemental analysis** calculated for C_19_H_38_N_3_Li: C, 72.34; H, 12.14: N, 13.32, found: C, 72.7; H, 12.31: N, 13.34.

**II: ^1^H NMR** (400 MHz, C_6_D_6_) δ 6.30 (dd, J = 10.3, 8.0 Hz, 1H), 4.51 (tdd, J = 6.3, 2.1, 1.0 Hz, 1H), 4.39 (dd, J = 10.3, 1.1 Hz, 1H), 3.92 (ddd, J = 8.1, 2.2, 1.1 Hz, 1H), 2.47 (d, J = 6.3 Hz, 2H), 2.22 (d, J = 0.8 Hz, 3H), 1.89 (s, 15H), 1.69 (s, 8H), 1.56 (s, 6H). **^13^C NMR** (101 MHz, C_6_D_6_) δ 139.8, 128.9, 103.6, 90.4, 76.1, 57.3, 54.0, 49.5, 45.6, 44.1, 40.9, 34.0, 26.9. **Elemental analysis** calculated for C_19_H_38_N_3_Na: C, 68.84; H, 11.55; N, 12.68, found: C, 68.11; H, 11.43: N, 12.43.

### 4.3 Stoichiometric Electrocyclization of *β*‐Ocimene

In a glovebox, an oven‐dried 30 mL Schlenk flask equipped with a magnetic stir bar was charged with pentane (8 mL), PMDETA (1.84 mL, 8.82 mmol, 2.0 equiv), and **1‐*E*** (>95% purity, 0.751 mL, 4.41 mmol, 1.0 equiv). AMTMP (4.41 mmol, 1.0 equiv) was added in one portion, and the reaction mixture was stirred at room temperature for 1 h. The reaction was quenched with water, extracted with pentane (3 × 5 mL), washed with 1 M HCl (2 × 5 mL), dried over MgSO_4_, filtered through a short silica pad, and concentrated under reduced pressure (350 mbar, 45°C, 1 h). Under these conditions, LiTMP afforded a 1:1:1 mixture of **4^a–c^** in 84% yield, whereas NaTMP gave a mixture of C_10_H_16_ isomers in 77% yield.

**4^a^: ^1^H NMR** (500 MHz, CDCl_3_) δ 5.86 (dt, J = 11.4, 5.7 Hz, 1H), 5.83 – 5.78 (m, 1H), 5.67 (t, J = 6.4 Hz, 1H), 1.92 (d, J = 6.0 Hz, 2H), 1.85 (d, J = 6.0 Hz, 2H), 1.78 (d, J = 1.4 Hz, 3H), 0.94 (d, J = 1.4 Hz, 6H). **^13^C NMR** (126 MHz, CDCl_3_) δ 134.9, 131.6, 131.4, 127.0, 43.4, 42.3 (2C), 29.2, 24.1. **HRMS (EI)** m/z: [M^•^]^+^ calculated for C_10_H_16_ 136.12465. Found 136.12478.

**4^b^: ^1^H NMR** (500 MHz, CDCl_3_) δ 5.57 – 5.42 (m, 3H), 2.24 (t, J = 5.9 Hz, 2H), 1.82 (s, 3H), 1.60 (t, J = 5.9 Hz, 2H), 1.03 (d, J = 1.1 Hz, 6H). **^13^C NMR** (126 MHz, CDCl_3_) δ 143.5, 141.6, 121.2, 120.4, 38.2, 37.5, 32.1, 30.2, 27.0. **HRMS (EI)** m/z: [M^•^]^+^ calculated for C_10_H_16_ 136.12465. Found 136.12485.

**4^C^: ^1^H NMR** (500 MHz, CDCl_3_) δ 5.49 (t, J = 7.0 Hz, 1H), 5.46 – 5.33 (m, 1H), 5.28 (d, J = 11.8 Hz, 1H), 2.70 (d, J = 5.3 Hz, 1H), 2.14 (d, J = 7.0 Hz, 1H), 1.74 (s, 1H), 0.94 (s, 6H). **^13^C NMR** (126 MHz, CDCl_3_) δ 141.2, 140.1, 123.1, 122.9, 39.3, 35.3, 32.7, 30.1, 25.4. **HRMS (EI)** m/z: [M^•^]^+^ calculated for C_10_H_16_ 136.12465. Found 136.12481.

### 4.4 Catalytic Electrocyclization of β‐Ocimene

For small‐scale catalytic reactions, a J. Young NMR tube equipped with a sealed capillary containing C_6_D_6_ was charged in the glovebox with β‐ocimene **1** (1.5 mmol, either 0.255 mL of pure **1‐*E*** or 0.341 mL of commercial **1‐*Z***, 74.4% purity) and PMDETA (0.0313 mL, 0.15 mmol, 10 mol%). After acquisition of a blank ^1^H NMR spectrum, the tube was returned to the glovebox and charged with AMTMP. After completion, the reaction mixture was quenched with two drops of water, extracted with pentane, dried over MgSO_4_, filtered through a short silica pad, and concentrated under reduced pressure (350 mbar, 45°C, 1 h). Using LiTMP (22.08 mg, 0.15 mmol, 10 mol%) and pure **1‐*E***, a 1:1 mixture of **4a** and **4b** was isolated in 61% yield. Using NaTMP (4.9 mg, 0.03 mmol, 2 mol%) together with PMDETA (15.7 µL, 0.075 mmol, 5 mol%) and pure **1‐*E***, a mixture of C_10_H_16_ isomers was obtained in 76% yield.

For preparative‐scale reactions, *β*‐ocimene **1** (5 mmol, either 0.852 mL of pure **1‐*E*** or 1.15 mL of commercial **1‐*Z***, 74.4% purity) and PMDETA (0.104 mL, 0.5 mmol, 10 mol%) were combined with LiTMP (10 mol%) in an ampoule under argon. After reaction completion, the mixture was quenched with water, washed with 1 M HCl, extracted with pentane, dried over MgSO_4_, filtered through a short silica pad, and concentrated under reduced pressure. With 95% pure **1‐*E***, a 1:1 mixture of **4^a^** ^−^ **^b^** was isolated in 84% yield. On 100 mmol scale from commercial **1‐*Z***, the reaction was exothermic and furnished 18 g of a 1:1 mixture of **4^a^** ^−^ **^b^** alongside unreacted limonene in 98% yield. Subsequent hydrogenation of **4^a^** ^−^ **^b^** with B_2_(OH)_4_ (2.1 equiv) and Pd/C (10 mol%) in DCM/H_2_O at room temperature overnight afforded **3** in 80% yield after filtration through Celite and silica and removal of the solvent.

**3: ^1^H NMR** (400 MHz, CDCl_3_) δ 1.75 (m, 1H), 1.61 – 1.04 (m, 9H), 1.02 – 0.64 (m, 18H). **^13^C NMR** (101 MHz, CDCl_3_) δ 42.4, 40.4, 39.9, 36.9, 33.4, 31.7, 31.2, 30.6, 24.1, 22.3. Data agrees with those previously reported in the literature [43].

### 4.5 Catalytic Electrocyclization of ***7***

A J.Young NMR tube equipped with a flame‐sealed Wilmad capillary filled with C_6_D_6_ was charged with **7** (1.5 mmol, 204 mg, 1 equiv) and PMDETA (0.15 mmol, 0.0313 mL, 10 mol%). NaTMP (24.49 mg, 0.15 mmol, 0.1 equiv) was added in a single portion, the tube was removed from the glovebox and was heated to 40°C for 16 h. The reaction was quenched with water, extracted with pentane and filtered through silica to obtain **8^a^** as a colorless oil (171 mg, 84% yield.) **^1^H NMR** (400 MHz, CDCl_3_) δ 5.80 – 5.71 (m, 1H), 5.71 – 5.62 (m, 2H), 2.03 (d, *J* = 2.0 Hz, 4H), 1.84 (d, *J* = 1.4 Hz, 3H), 0.97 (s, 6H). **^13^C NMR** (101 MHz, CDCl_3_) δ 140.6, 129.7, 126.5, 122.0, 48.8, 43.9, 43.9, 36.8, 29.3, 27.5, 27.5. **HRMS (EI)** m/z: [M^•^]^+^ calculated for C_10_H_16_ 136.12465. Found 136.12478.

### 4.6 Catalytic Electrocyclization of ***9***

A J.Young NMR tube was charged in a glovebox with **9 (**0.5 mmol, 88.2 mg, 1 equiv), PMDETA (0.05 mmol, 0.0313 mL, 10 mol%) and C_6_D_6_ (0.5 mL). NaTMP (8.16 mg, 0.05 mmol, 10 mol%) was added in a single portion and the reaction was quenched after 90 min with water, extracted with pentane and filtered through silica to obtain a mixture of **10^a^** and **10^b^** in a 2:1 ratio as a colorless oil (86.2 mg, 98% yield).

**10^a^**: **^1^H NMR** (400 MHz, CDCl_3_) δ 5.73 – 5.70 (m, 2H), 5.56 (m, 1H), 5.11 (m, 1H), 2.35 – 2.26 (m, 4H), 2.15 – 1.97 (m, 4H), 1.89 – 1.78 (m, 2H), 1.68 (s, 3H), 1.61 (s, 3H). **^13^C NMR** (101 MHz, CDCl_3_) δ 146.5, 132.0, 131.7, 125.1, 124.2, 120.6, 41.0, 34.9, 32.2, 27.2, 25.9, 25.8, 17.8. **HRMS (EI)** m/z: [M^•^]^+^ calculated for C_13_H_20_ 176.15595. Found 176.15620.

**10^b^**: **^1^H NMR** (400 MHz, CDCl_3_) δ 5.83 (dt, J = 10.8, 5.1 Hz, 1H), 5.68 (m, 1H), 5.67 – 5.60 (m, 1H), 5.11 (m, 1H), 2.36 – 2.25 (m, 2H), 2.22 (q, J = 5.8 Hz, 2H), 2.17 – 1.96 (m, 4H), 1.88 – 1.79 (m, 1H), 1.69 (s, 3H), 1.60 (s, 3H). **^13^C NMR** (101 MHz, CDCl_3_) δ 142.5, 136.9, 131.6, 129.1, 128.8, 124.3, 39.8, 31.7, 30.0, 28.4, 27.8, 25.9, 25.8. **HRMS (EI)** m/z: [M^•^]^+^ calculated for C_13_H_20_ 176.15595. Found 176.15614.

## Conflicts of Interest

The authors declare no conflicts of interest.

## Supporting information



Experimental details, copies of the NMR spectra, characterization data and X‐Ray crystallographic details are available as Supporting Information.
CCDC codes 2530007‐2530008‐2530009 contains the crystallographic data for structures reported in this article and can be accessed at: https://www.ccdc.cam.ac.uk.
All experimental data (NMR FID, CIF Files, HRMS & Elemental Analysis) are openly accessible via Zenodo repository: https://doi.org/10.5281/zenodo.18713127
All DFT data underlying the present work, including inputs and outputs are openly accessible via ioChem‐BD repository: https://doi.org/10.19061/iochem‐bd‐6‐630
The authors have cited additional references within the Supporting Information [67, 68, 69, 70, 71, 72, 73, 74, 75, 76, 77, 78, 79, 80, 81, 82, 83, 84, 85, 86].

## Data Availability

The data that support the findings of this study are openly available in Zenodo (NMR FID, CIF Files, HRMS & Elemental Analysis) at https://doi.org/10.5281/zenodo.18713127 and in ioChem‐BD (DFT calculations) at https://doi.org/10.19061/iochem‐bd‐6‐630. Deposition Numbers [CCDC 2530007‐2530008‐2530009] contains the supplementary crystallographic data for this paper, which can be obtained free of charge from The Cambridge Crystallographic Data Centre at www.ccdc.cam.ac.uk
