# Supplementary material for: Alkali‐Metal Base Catalyzed Electrocyclization of Isoprene Derivatives
Source: Chemistry. 2026 May 3;32(27):e71057. doi: 10.1002/chem.71057 (PMC13380371; doi:10.1002/chem.71057)
Supplement: Supplementary file 1 — Experimental details, copies of the NMR spectra, characterization data and X‐Ray crystallographic details are available as Supporting Information. CCDC codes 2530007‐2530008‐2530009 contains the crystallographic data for structures reported in this article and can be accessed at: https://www.ccdc.cam.ac.uk. All experimental data (NMR FID, CIF Files, HRMS & Elemental Analysis) are openly accessible via Zenodo repository: https://doi.org/10.5281/zenodo.18713127 All DFT data underlying the present work, including inputs and outputs are openly accessible via ioChem‐BD repository: https://doi.org/10.19061/iochem‐bd‐6‐630 The authors have cited additional references within the Supporting Information [67, 68, 69, 70, 71, 72, 73, 74, 75, 76, 77, 78, 79, 80, 81, 82, 83, 84, 85, 86]. [file CHEM-32-e71057-s001.pdf]

Alkali-Metal Base Catalyzed Electrocyclization of Isoprene Derivatives

Mikaël Le Roch, Krzysztof Piech, Aurélien Crochet and Andreu Tortajada*.

Department of Chemistry, University of Fribourg. Chemin du Musée 9, 1700 Fribourg, Switzerland.

Table of Contents

General Methods ***4***

Data management ***6***

Purification of commercial ocimene ***7***

Procedure 7
Spectrum 8

Synthesis of Organometallic Complexes ***12***

Synthesis of LiTMP **12**

Synthesis of NaTMP **12**

Synthesis of I **13**

Procedure 13
Characterization spectra 14

Synthesis of II **16**

Procedure 16
Characterization spectra 17

X-Ray Crystallographic Data ***19***

Stoichiometric electrocyclization of β-ocimene ***20***

General procedure **20**

LiTMP electrocyclization **20**

Procedure 20
Characterization spectrum 21
Prep-GC isolation 22

NaTMP electrocyclization **24**

Procedure 24
Spectrum 24

Catalytic electrocyclization of β-ocimene ***25***

General procedure **25**

LiTMP electrocyclization **26**

Procedure 26
Characterization spectrum 26
In-situ monitoring of the reaction with LiTMP 27
In-situ monitoring of the reaction with **I** 28

NaTMP electrocyclization **29**

Procedure 29
Spectrum 29
Derivatization of $C_{10}H_{16}$ isomers from NaTMP catalytic electrocyclization 30

Scale up and hydrogenation **32**

Pure 1-*E* 32
Commercial 1-*Z* 34

Electrocyclization of trienes ***36***

Catalytic electrocyclization **36**

Successful trials 36
Unsuccessful trials 37

Synthesis of starting materials **41**
7 41
9 41
S_4 43
S_5 43
S_8 44
S_{12} 45

DFT calculations ***47***

Computational method ***47***

Stoichiometric deprotonation and cyclization **48**

Catalytic turnover **49**
Deprotonation of **I** at C_3 49
Deprotonation of **I** at C_5 50
Deprotonation of **I** at C_7 50
Implicit solvation 51

NMR spectra of new products ***52***

Starting materials **52**

Cyclized products **61**

General Methods

All procedures were conducted using standard Schlenk line and glove box techniques under an inert atmosphere of Argon. Pentane was degassed, purified and collected via an MBraun SPS 5 and stored over 4 Å molecular sieves for at least 24 hours prior to use. Deuterated solvents (C_6D_6) were purchased from Sigma Aldrich or Eurisotope, dried over NaK alloy for 16 hours and then cycled through three rounds of degassing by employing a freeze-pump-thaw method. The deuterated solvents were then collected via vacuum transfer and stored under argon atmosphere over 4 Å molecular sieves.

All substrates employed in this study were degassed by freeze-pump-thaw and stored over molecular sieves. The polydentate amines used in this study were dried over calcium hydride, distilled under reduced pressure and stored over molecular sieves prior to their use.

NMR spectra (^{1}H, ^{13}C) were acquired at room temperature using either Bruker spectrometers operating at 300 MHz, 400 MHz or 500 MHz. Chemical shifts are reported in parts per million (ppm), with the residual non-deuterated $CDCl_3$ or C_6D_6 signals serving as the reference (δ ^{1}H = 7.26 ppm; δ ^{13}C = 77.16 ppm / δ ^{1}H = 7.16 ppm; δ ^{13}C = 128.05 ppm respectively). The following abbreviations were used to describe the observed signal multiplicities: s (singlet), d (doublet), t (triplet), q (quartet), p (quintet), hept (heptet), m (multiplet), and br (broad) when the peak is broad and the precise multiplicity cannot be determined. NMR Yields are determined using 1,3,5-trimethoxybenzene as internal standards.

Diffusion Ordered Spectroscopy (DOSY) experiments were conducted by NMR using the External Calibration Curve (ECC) method in C_6D_6 as described by Stalke.[1] Data was accumulated by linearly varying the diffusion encoding gradients over a range of 2% to 95% for 24 gradient increment values. The signal decay dimension on the pseudo-2D data was generated by Fourier transformation of the time-domain data. The diffusion profile and coefficients were ascertained by use of the DOSY processing features of TopSpin software.

Elemental analyses (C, H and N) were conducted with a Flash 2000 Organic Elemental Analyser (Thermo Scientific). Samples were prepared in the glovebox under argon atmosphere and sealed in an air-tight container prior to analyses. All results were obtained by the Analytical Research and Services Schürch Group of the University of Bern. Samples were weighed on a Mettler Toledo balance with +2 µg resolution and sample weights from 1-3 mg were used. The presented values are the average of determinations in triplicate to ensure consistency (if m(sample)>1 mg).

High-resolution mass spectra were recorded by gas chromatography–mass spectrometry using a Trace GC Ultra (Thermo Scientific) coupled to a DFS double-focusing magnetic sector mass spectrometer. All results were obtained by the Analytical Services Group of the University of Zurich. Analyses were performed under electron

---

[1] R. Neufeld, D. Stalke. *Chem. Sci.*, **2015**, *6*, 3354–3364.

ionization (EI, 70 eV), electron spray ionization (ESI) or chemical ionization (CI, isobutane as reagent gas) using helium as the carrier gas. For mixtures of isomers, the HRMS reported are according to the prior separation by GC.

GC–MS analyses were carried out using a Clarus 560S mass spectrometer coupled to a Clarus 500 gas chromatograph (PerkinElmer) equipped with an autosampler and an Elite-5MS capillary column (30 m × 0.25 mm i.d., 0.5 μm film thickness). Helium was used as the carrier gas. Samples (0.5 μL in dichloromethane) were injected in split mode (1:20). The injector temperature was held at 180 °C for 1 min and then increased to 250 °C for the remainder of the run. The oven temperature program was as follows: 50 °C for 2 min, ramped to 110 °C at 10 °C min^{-1} and held for 10 min, then increased to 310 °C at 25 °C min^{-1} and held for 4 min. Data acquisition was performed after a 2.6 min solvent delay.

Semi-preparative GC isolation was carried out using a Clarus 580 gas chromatograph (PerkinElmer) equipped with an autosampler, a packed column (6 ft × 1/8 in stainless steel, 10% OV-101 on W-NAW 80/100; PerkinElmer, product no. N6101406) and coupled with a TCD detector. 5 microliters of non-diluted compounds were injected in a splitless mode with the injector temperature set to 200 °C. The oven temperature program was as follows: 50 °C for 5 min, ramped to 100 °C at 3 °C min^{-1} and held for 5 min, then increased to 200 °C at 30 °C min^{-1} and held for 5 min. Collection was achieved by connecting the detector outlet to a 15 cm Teflon tubing attached to a Pasteur pipette inserted into an NMR tube cooled in an ice–water bath at 0 °C.[2]

Thin-layer chromatography (TLC) was carried out on Merck silica gel 60 F-254 plates, and chromatograms were visualized under UV light (λ = 254 nm) and/or after staining with suitable color reagents ($KMnO_4$), followed by heating.

Flash column chromatography was performed with Fisher Davisil SI 60 silica gel (35–70 μm) using the indicated mobile phases.

Single-crystal X-ray diffraction data were collected on a STOE (Darmstadt, Germany) STADIVARI diffractometer using Cu Kα1 radiation (λ = 1.54186 Å). Suitable crystals were selected, mounted on an oil-coated loop, and maintained at 250(2) K during data collection. Data reduction and absorption corrections were performed using the STOE program package.[3] Using Olex2,[4] structures were solved with a SHELXT [5] structure solution program using Intrinsic Phasing and refined with the SHELXL[6] refinement package using least square minimization. H-atoms were assigned in geometrically calculated positions and refined using a riding model where each H-atom was assigned a fixed isotropic displacement parameter with a value equal to 1.2Ueq of its parent atom (1.5Ueq for methyl groups). In both structures, disorder model was included for parts of

[2] A.R. Bressette. *J. Chem. Educ.* **2001**, *78*, 3, 366.
[3] Stoe (2016). *X-AREA* and *X-RED32*. Stoe & Cie, Darmstadt, Germany.
[4] O.V. Dolomanov, L.J. Bourhis, R.J. Gildea, J.A.K. Howard, H. Puschmann. *J. Appl. Crystallogr.* **2009**, *42*, 339–341.
[5] G.M. Sheldrick. *Acta Crystallogr. A Found. Adv.* **2015**, *71*, 3–8.
[6] G.M. Sheldrick. *Acta Crystallogr. C Struct. Chem.* **2015**, *71*, 3–8.

the structure where the occupancies of each disorder component were refined using a free variable.

Data management

All experimental data (NMR FID, CIF Files, HRMS & Elemental Analysis) are openly accessible via Zenodo repository : https://doi.org/10.5281/zenodo.18713127

All DFT data underlying the present work, including inputs and outputs are openly accessible via ioChem-BD repository : https://doi.org/10.19061/iochem-bd-6-630

Purification of commercial ocimene

Procedure

Commercial *Z*-*β*-ocimene (**1-*Z***) was purchased from several suppliers (Sigma-Aldrich, BLD, Apollo) and had a reported purity of 74.4%, with limonene as the main byproduct. This commercial material was used for crystallization and reaction optimization studies. For reactions requiring isolated yields, *β*-ocimene was separated from limonene using the following procedure:

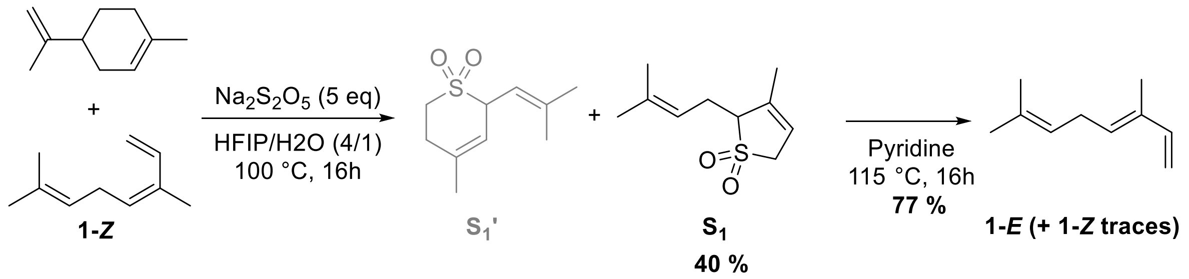

STEP 1

According to a reported procedure [7], to a 100 mL pressure tube equipped with a stirrer was added 40 mL of HFIP (1,1,1,3,3,3-hexafluoro-2-propanol) and 10 mL of deionized water. The pressure tube was closed with a septum and the mixture degassed 5 minutes by bubbling Argon. After degassing, commercial ocimene (74,4 % purity, 4.35 mL, 20 mmol, 1 eq) and $Na_2S_2O_5$ (19.01g, 100 mmol, 5 eq) were added. The pressure tube was then closed and the mixture stirred for 16 h at 100 °C. Then, the reaction mixture was cooled down to room temperature, the mixture filtrated through a filter to remove excess $Na_2S_2O_5$ and the solvent removed by evaporation. The biphasic mixture was subsequently extracted with EtOAc (3 × 30 mL), and the combined organic layers were concentrated under reduced pressure to remove the unreacted limonene and solvent. The crude residue was then purified by column chromatography (Et_2O/pentane 2:3, Rf = 0.41), enabling the separation of the target product **S_1** (translucent oil, 1.6 g, 40% yield) from a rearranged side-product **S_1’**.

S_1 : ^{1}H NMR (400 MHz, $CDCl_3$) δ 5.70 – 5.63 (m, 1H), 5.20 (dddq, *J* = 8.6, 5.9, 3.0, 1.5 Hz, 1H), 3.75 – 3.56 (m, 2H), 3.56 – 3.43 (m, 1H), 2.61 – 2.47 (m, 2H), 1.84 (dp, *J* = 3.9, 1.2 Hz, 3H), 1.71 (dt, *J* = 3.1, 1.4 Hz, 3H), 1.68 – 1.62 (m, 3H). **^{13}C NMR** (101 MHz, $CDCl_3$) δ 138.9, 135.6,118.5, 117.3, 67.5, 55.8, 26.6, 25.9, 18.4, 18.1.
Data agrees with those previously reported in the literature.[8]

S_1’ : ^{1}H NMR (400 MHz, $CDCl_3$) δ 5.20 (dq, *J* = 3.2, 1.5 Hz, 1H), 5.06 (dp, *J* = 9.8, 1.5 Hz, 1H), 4.37 (dt, *J* = 9.8, 2.7 Hz, 1H), 3.07 (dd, *J* = 7.1, 5.6 Hz, 2H), 2.71 (dt, *J* = 15.5, 7.3 Hz, 1H), 2.57 (dt, *J* = 18.1, 5.7 Hz, 1H), 1.83 (d, *J* = 1.4 Hz, 3H), 1.81 – 1.75 (m, 6H). **^{13}C NMR** (101 MHz, $CDCl_3$) δ 142.8, 133.9, 120.5, 114.6, 58.4, 46.3, 30.5, 26.3, 24.10, 18.9.
Data agrees with those previously reported in the literature. [9]

---

[7] H. T. Dang, V. T. Nguyen, V. D. Nguyen, H. D. Arman, O. V. Larionov. *Org. Biomol. Chem.* **2018**, *16* (19), 3605–3609.
[8] L. A. Pasfield, L. de la Cruz, J. Ho, M. L. Coote, G. Otting, M. D. McLeod. *Asian J. Org. Chem.* **2013**, *2* (1), 60–63.
[9] W.-R. Abraham, H.-A. Arfmann. *Tetrahedron* **1992**, *48* (32), 6681–6688.

STEP 2

In a two-neck round-bottom flask equipped with a stirrer and a condenser, **S_1** (3.16 g, 15.8 mmol, 1 equiv) was dissolved in 30 mL of distilled pyridine. The mixture was then refluxed at 115 °C for 16 hours. After cooling to room temperature, 30 mL of 1 M HCl was added, and the mixture was extracted with pentane (3 × 20 mL). The combined organic layers were filtered through a silica pad and concentrated under reduced pressure (volatile product, 350 mbar for 1 h at 45 °C) to afford >95 % pure **1-*E*** (+ **1-*Z*** traces) (translucent liquid, 1.65g, 77% yield)

1-*E* : **^{1}H NMR** (400 MHz, $CDCl_3$) δ 6.37 (ddd, *J* = 17.4, 10.7, 0.7 Hz, 1H), 5.46 (t, *J* = 7.21 Hz, 1H), 5.17 – 5.04 (m, 2H), 4.93 (d, *J* = 10.7 Hz, 1H), 2.83 (t, *J* = 7.4 Hz, 2H), 1.77 (q, *J* = 1.0 Hz, 3H), 1.70 (t, *J* = 1.4 Hz, 3H), 1.65 (d, *J* = 1.4 Hz, 3H). **^{13}C NMR** (101 MHz, $CDCl_3$) δ 141.7, 133.9, 132.3, 131.9, 122.3, 110.7, 27.5, 25.8, 17.9, 11.8.
Data agrees with those previously reported in the literature. [10]

Spectrum

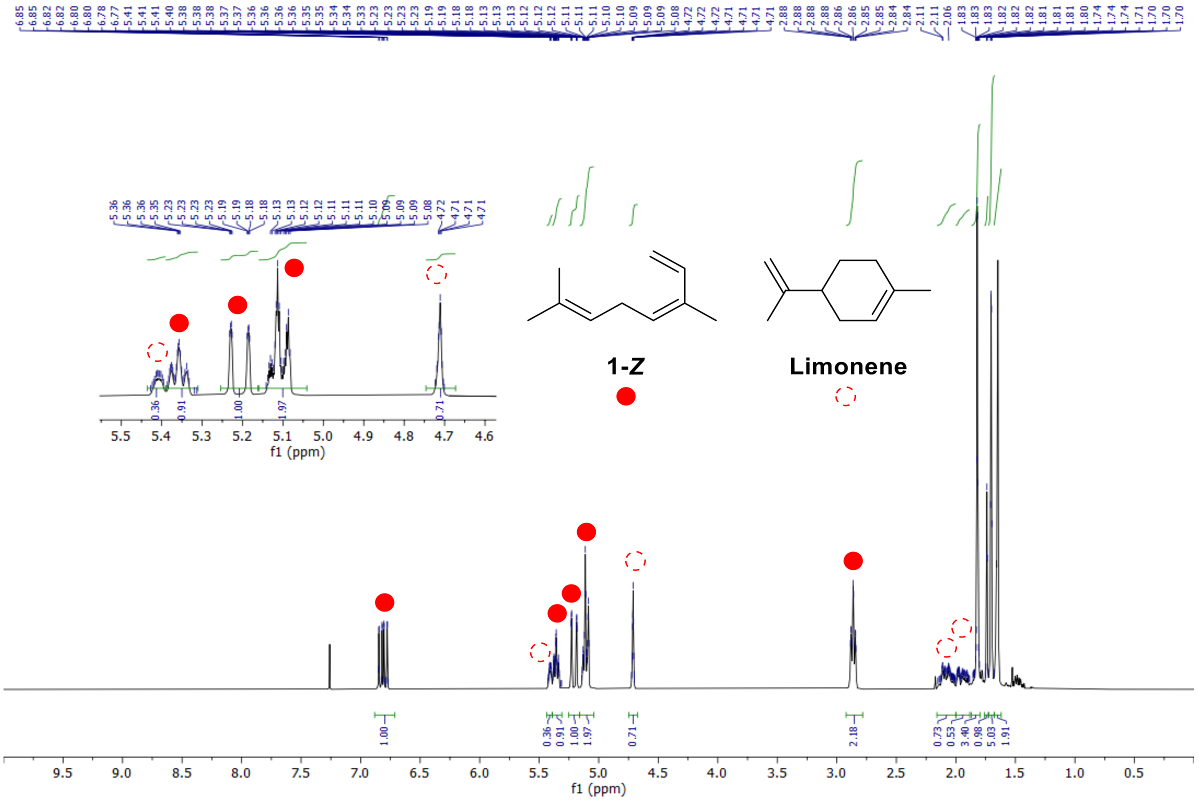

*Figure S1 : ^{1}H NMR spectrum of commercial **1-Z** ($CDCl_3$).*

[10] S. Yildizhan, S. Schulz. *Synlett* **2011**, *2011* (19), 2831–2833.

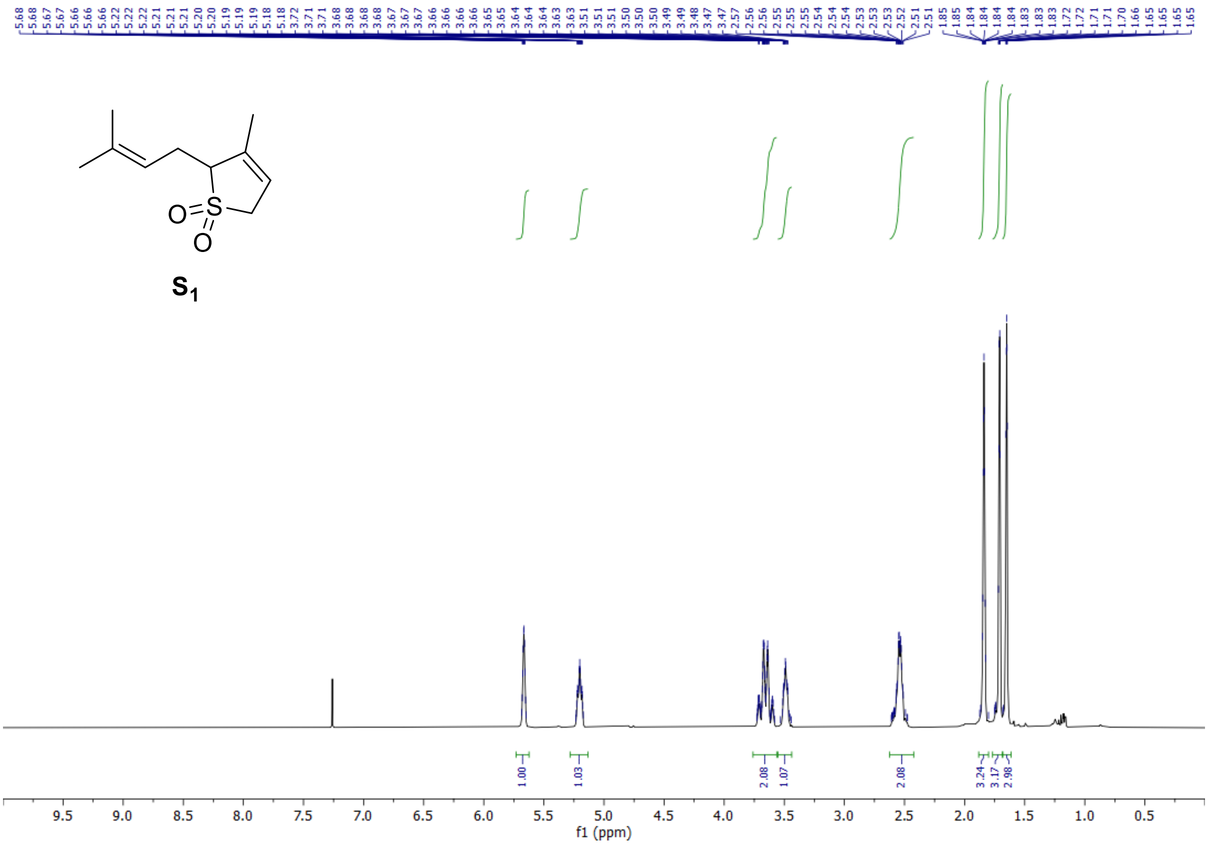

Figure S2 : ^{1}H NMR spectrum of S_1 ($CDCl_3$).

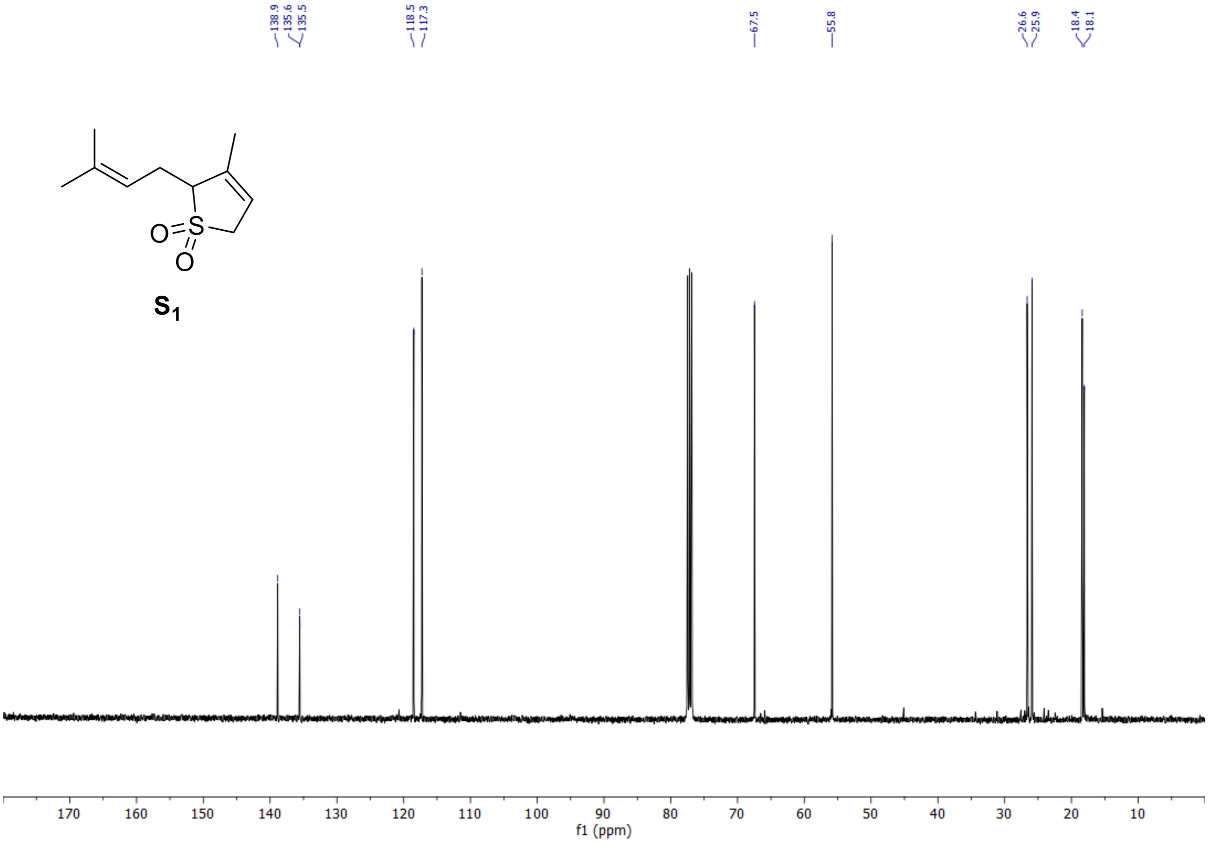

Figure S3 : ^{13}C NMR spectrum of S_1 ($CDCl_3$).

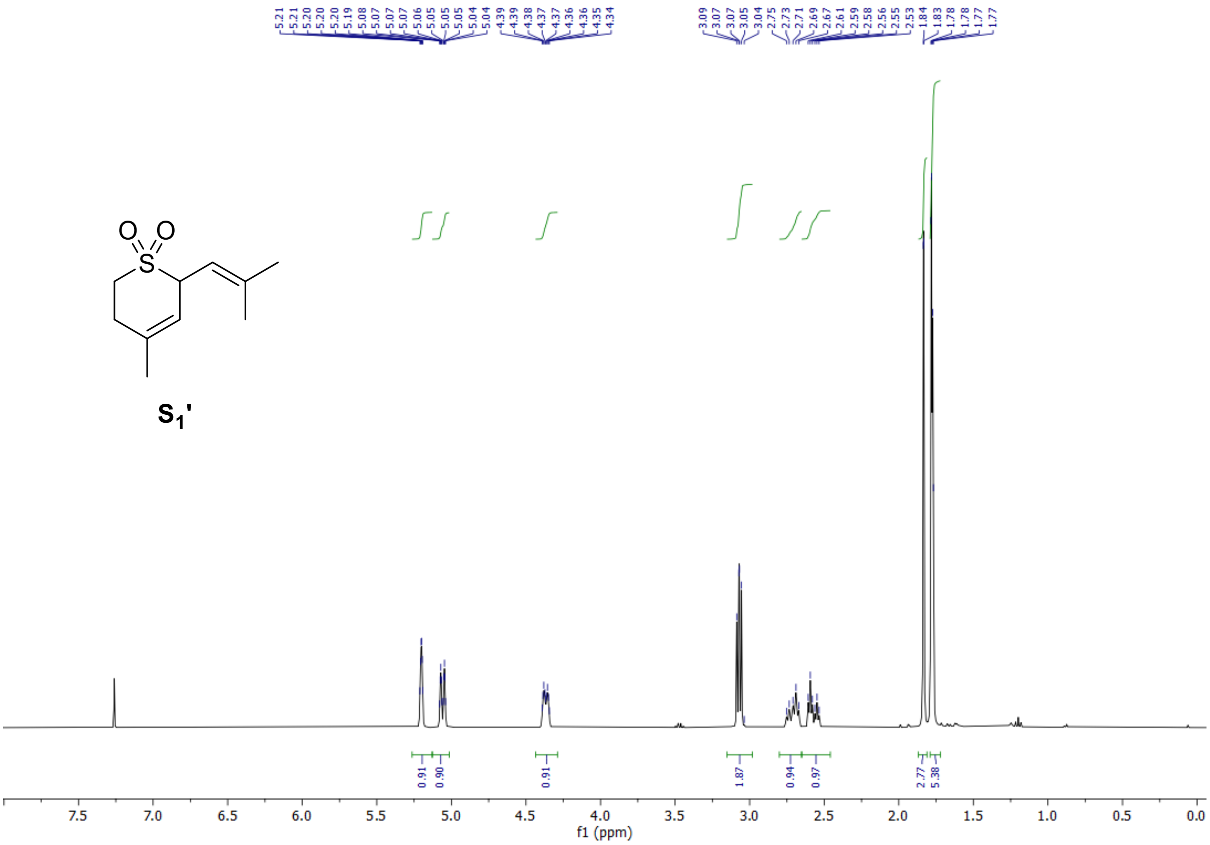

Figure S4 : ¹H NMR spectrum of S_1' ($CDCl_3$).

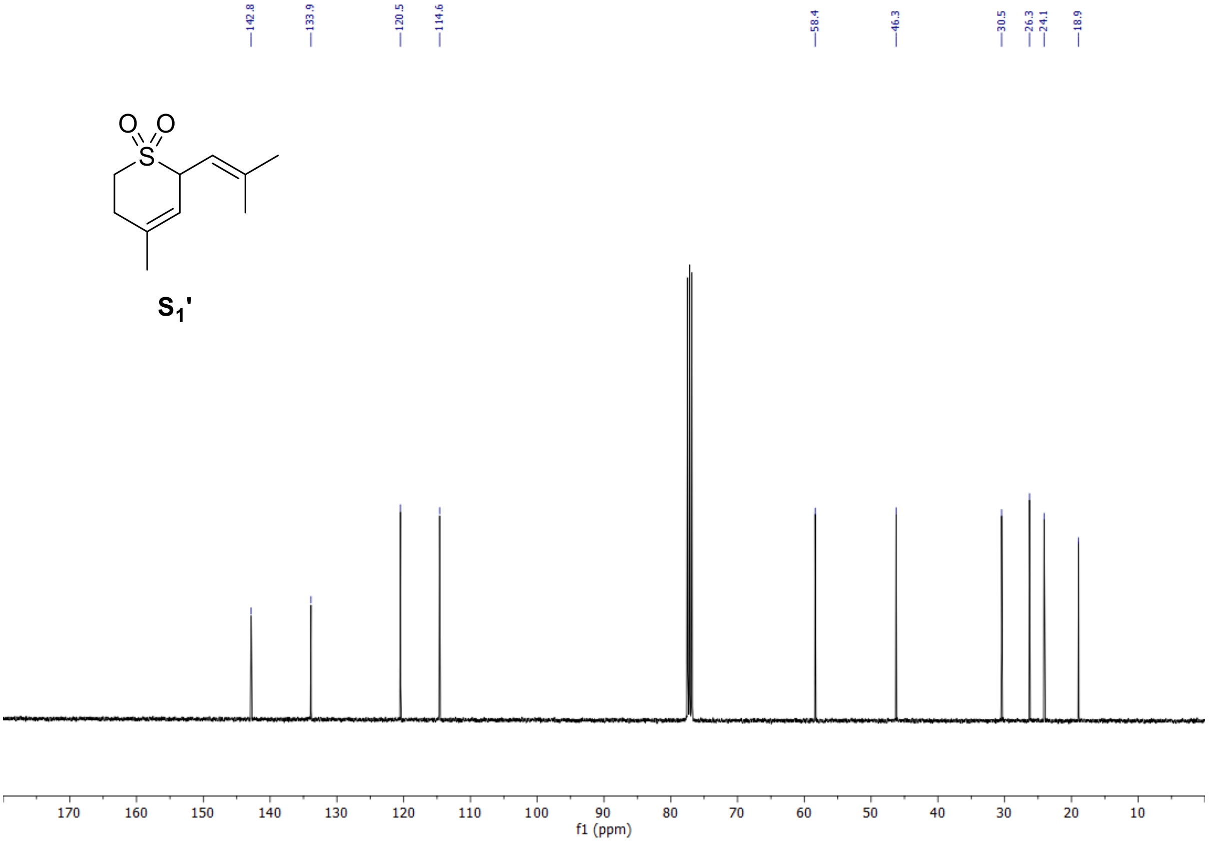

Figure S5 : ¹³C NMR spectrum of S_1' ($CDCl_3$).

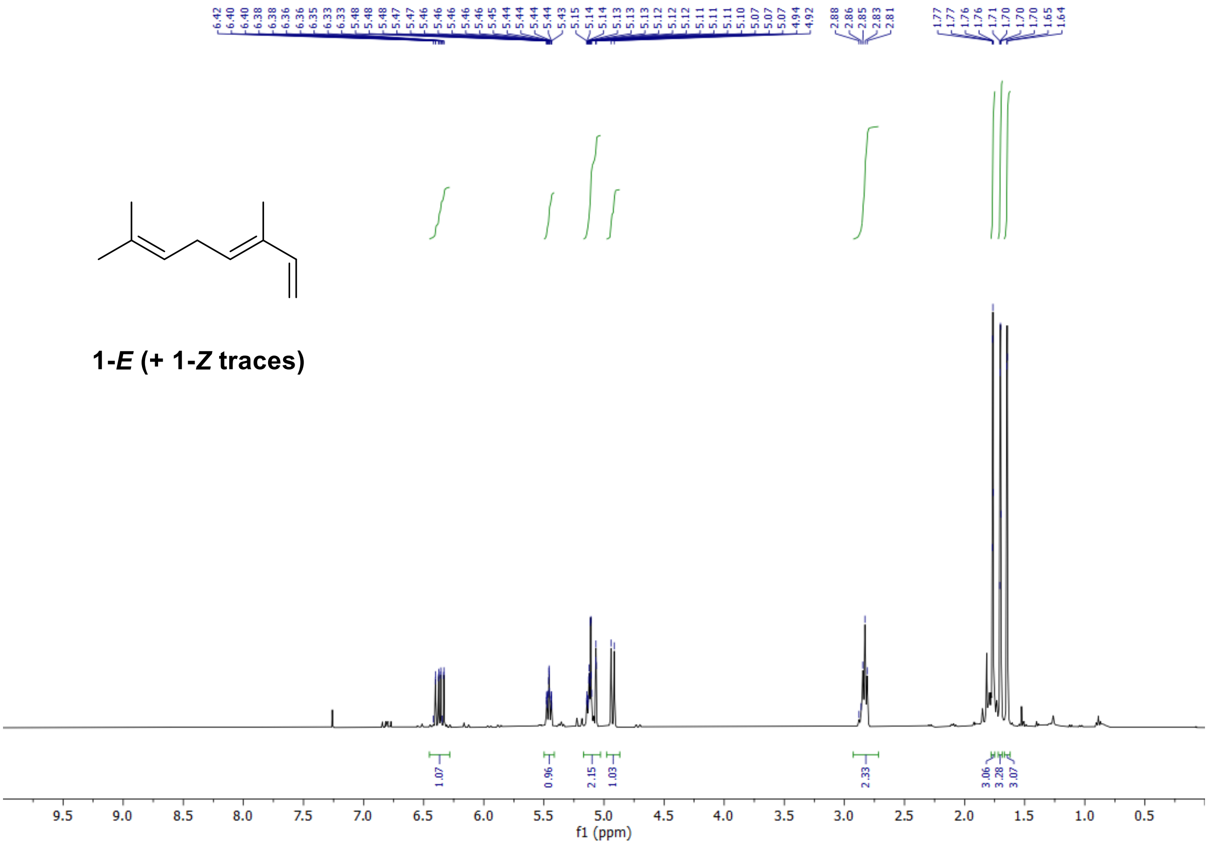

Figure S6 : ^{1}H NMR spectrum of 1-E (CDCl₃).

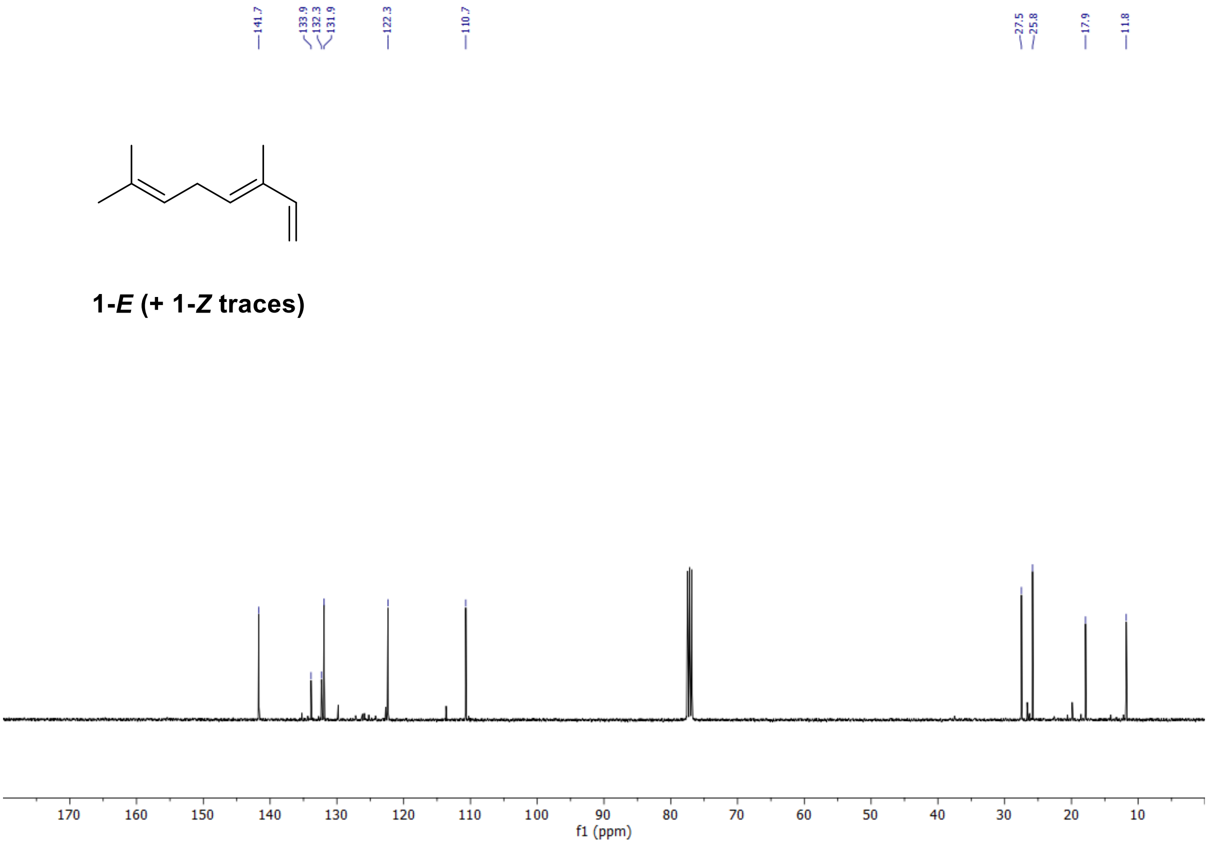

Figure S7 : ^{13}C NMR spectrum of 1-E (CDCl₃).

Synthesis of Organometallic Complexes

Synthesis of LiTMP

In an argon-filled Schlenk flask, 25 mL of dry pentane were added 5.1 mL (30 mmol) of TMP(H), and the mixture was cooled to 0 °C. This was followed by the dropwise addition of 13.2 mL (33 mmol) of 2.5 M *n*BuLi in hexanes which rapidly afforded a thick, white suspension. The suspension was warmed to ambient temperature and stirred overnight. Isolation of the white precipitate (LiTMP) was achieved by gravity filtration using a glass-tapped filter frit. The filter cake was washed with 3 x 15 mL aliquots of fresh pentane until the liquors ran completely clear. The solid was dried under vacuum and stored in the glovebox for further use. Typical yield = 88 %, 3.88 g.

Synthesis of NaTMP

In an argon-filled Schlenk flask, 50.0 mL of dry pentane were added to 3.84 g (40 mmol) of NaOtBu affording a fine, white suspension which was cooled to 0 °C. This was followed by the dropwise addition of 25.2 mL (40 mmol) of 2.5 M *n*BuLi in hexanes which rapidly afforded a thick, white suspension. A further 20 mL of pentane was added at this stage to ensure sufficient mobility in the mixture. The suspension was warmed to ambient temperature and stirred overnight. Isolation of the white precipitate (*n*BuNa) was achieved by gravity filtration under argon using a glass-tapped filter frit. The filter cake was washed with 3 x 15 mL aliquots of fresh pentane until the liquors ran completely clear. The solid was dried under vacuum and stored in the glovebox for further use. Typical yield = 90 %, 2.9 g.

2.88 g (36 mmol) of *n*BuNa was transferred into a clean Schlenk inside the glovebox and then suspended in 40 mL of dry pentane on a Schlenk line. The suspension was cooled to 0 °C with continuous, vigorous stirring. 6.12 mL (36 mmol) of TMP(H) were added dropwise, affording a pale-yellow suspension which was stirred at ambient temperature overnight. NaTMP was isolated the following day via gravity filtration and washed with 3 x 10 ml aliquots of fresh pentane. The resultant off-white solid was dried under vacuum and stored inside an argon-filled glovebox. Typical yield = 80 %, 4.7 g.

Synthesis of I

Procedure

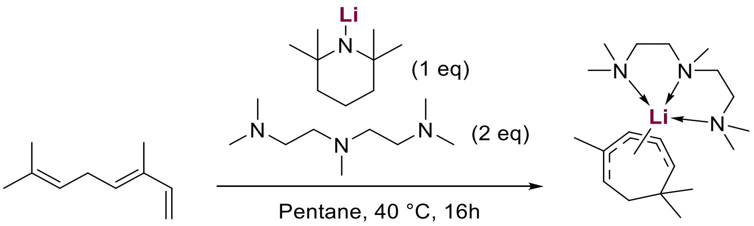

1-*E* **I** 20 %

In an oven-dried ampoule equipped with a magnetic stirrer and placed in a glovebox, pentane (5 mL), PMDETA (1.0 mmol, 0.209 mL, 2 equiv), and commercial ocimene (74.4% purity, 0.5 mmol, 0.114 mL, 1 equiv) were added. LiTMP (0.5 mmol, 73.6 mg, 1 equiv) was then added in one portion, the ampoule was closed, removed from the glovebox, and heated at 40 °C for 1h. The resulting mixture was filtered via cannula transfer into an oven-dried Schlenk flask and stored at −40 °C. After one night, the supernatant was removed via cannula filtration and the solid was washed 3 times with cold pentane to afford yellow crystals (32 mg, 20% yield).

Scalable powder isolation can be performed on a 2.5 mmol scale by removing the supernatant after the reaction at 40°C in 1h and washing the resulting yellow/brown solid 3x with cold pentane to afford **I** as a yellow powder (374 mg, 48 % yield.).

I : **^{1}H NMR** (400 MHz, C_6D_6) δ 5.98 (dd, *J* = 10.3, 7.6 Hz, 1H), 4.75 (tdd, *J* = 6.4, 2.2, 1.0 Hz, 1H), 4.56 (d, *J* = 10.3 Hz, 1H), 3.46 (dd, *J* = 7.7, 2.3 Hz, 1H), 2.45 (d, *J* = 6.5 Hz, 2H), 2.17 (d, *J* = 1.0 Hz, 3H), 2.01 (s, 4H), 1.91 (s, 11H), 1.82 – 1.68 (m, 8H), 1.58 (s, 6H). **^{13}C NMR** (101 MHz, C_6D_6) δ 141.6, 128.5, 107.1, 94.1, 71.3, 57.4, 54.1, 48.2, 45.7, 40.2, 34.5, 27.0. **Elemental analysis** calculated for $C_{19}H_{38}N_3Li$: C, 72.34; H, 12.14: N, 13.32, found : C, 72.7; H, 12.31: N, 13.34.

^{1}H NMR DOSY : In a J. Young NMR tube, approx. 4 mg of compound **I** was dissolved in C_6D_6 forming of a light-yellow clear solution. The NMR tube was shaken until complete dissolution of the base. Using TMS as an internal reference standard, we have been able to calculate a molecular weight (MWdet) of 327 g/mol with DSE model (4% error).

Characterization spectra

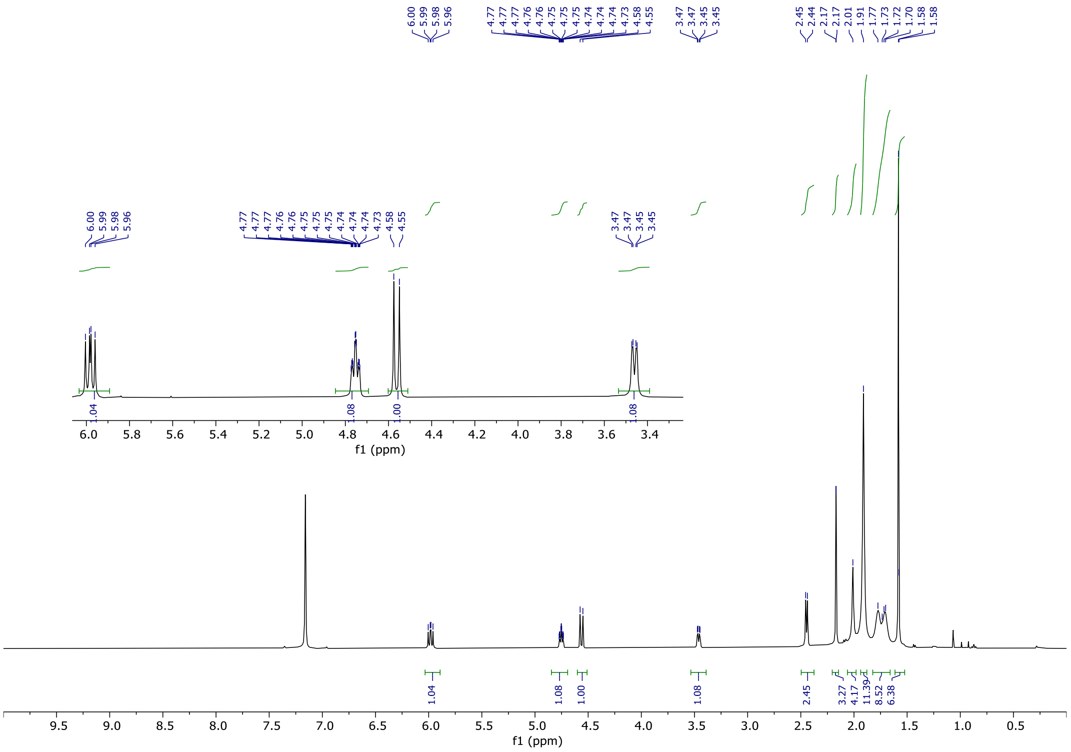

Figure S8 : ^{1}H NMR spectrum of **I** (C_6D_6).

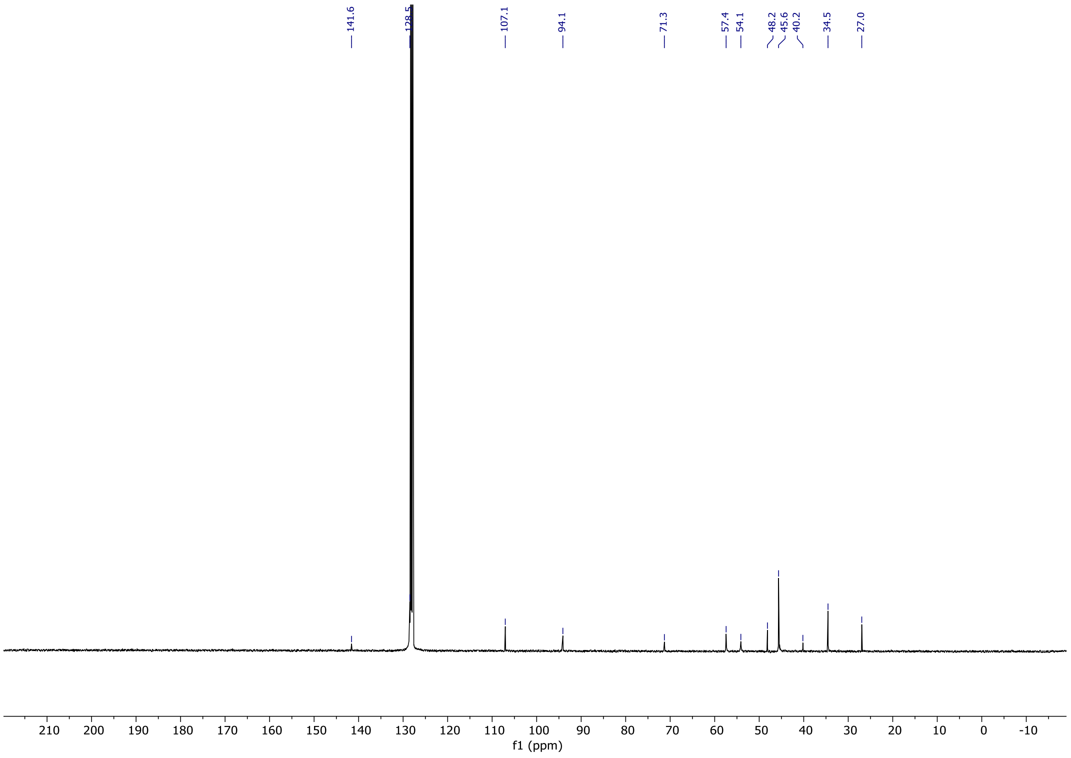

Figure S9 : ^{13}C NMR spectrum of **I** (C_6D_6).

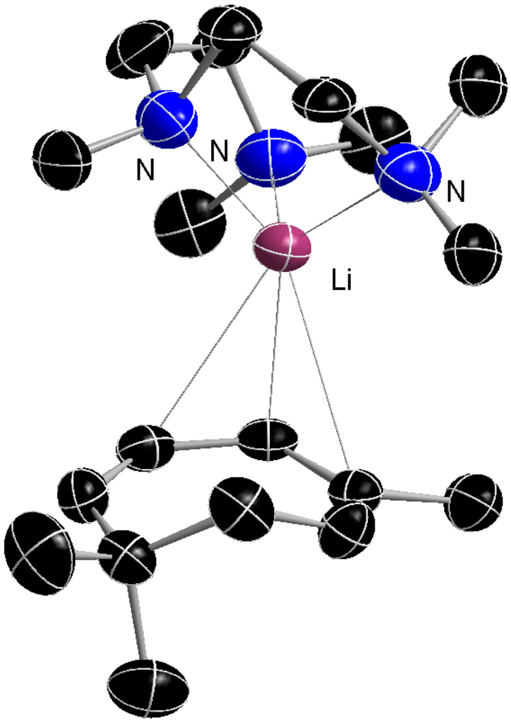

*Figure S10 : XRD structure of **I**, ellipsoids are displayed at 30 % probability and H/disorder has been omitted for clarity.*

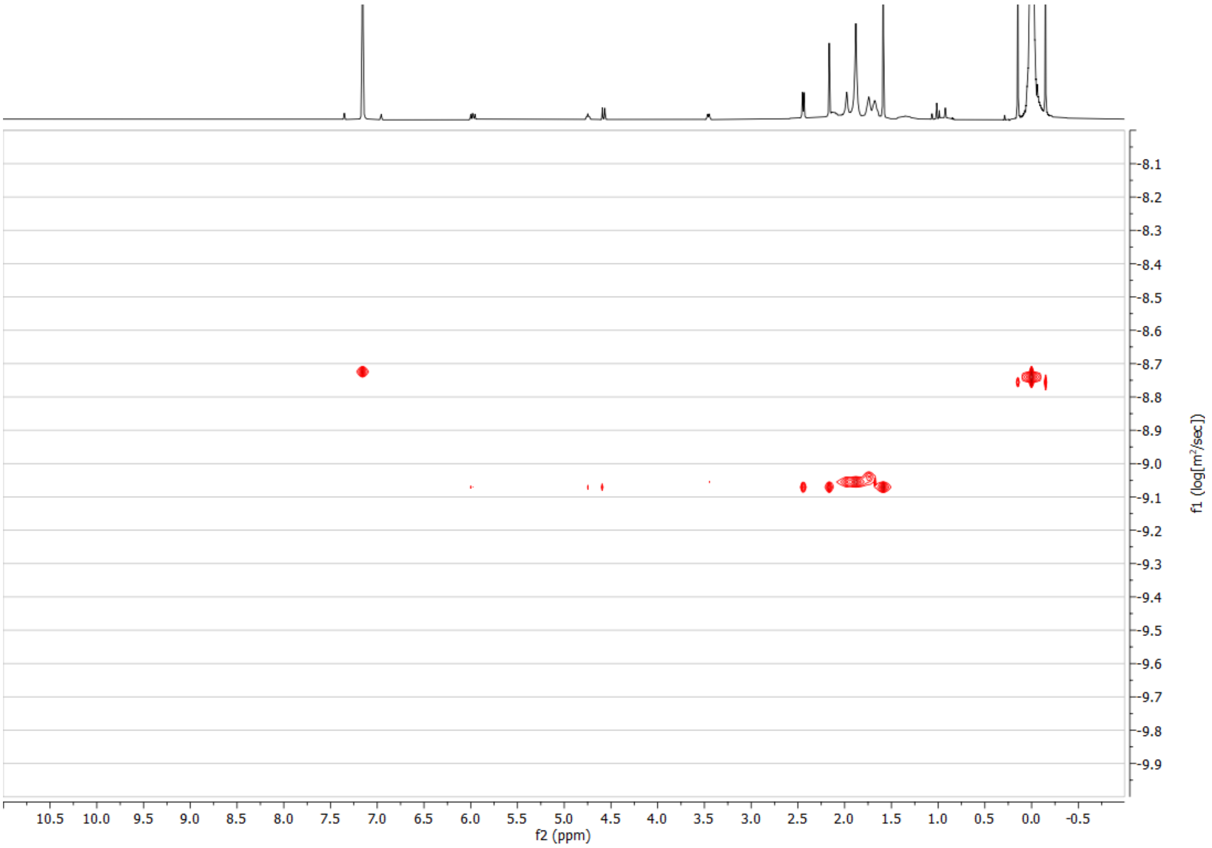

*Figure S11 : ¹H DOSY NMR of **I** (C_6D_6). Diffusion coefficient for TMS: 1.808 E^{-9} m^2/s, I : 8.669 E^{-10} m^2/s. MW estimated = 327 g·mol^{-1}.*

Synthesis of II

Procedure

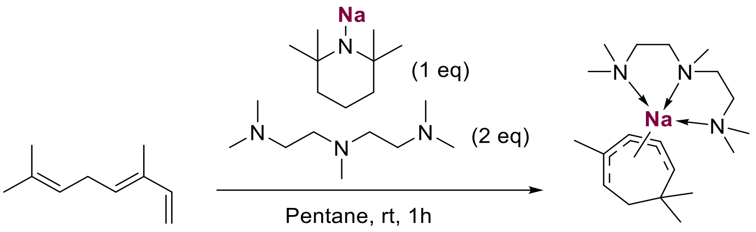

II 22 %

In an oven-dried ampoule equipped with a magnetic stirrer and placed in a glovebox, pentane (5 mL), PMDETA (1.0 mmol, 0.209 mL, 2 equiv), and commercial ocimene (74.4% purity, 0.5 mmol, 0.114 mL, 1 equiv) were added. NaTMP (0.5 mmol, 81.6 mg, 1 equiv) was then added in one portion, the ampoule was closed, removed from the glovebox, and stirred for 1h. The resulting mixture was filtered via cannula transfer into an oven-dried Schlenk flask and stored at −40 °C. After one night, the supernatant was removed via cannula filtration and the solid was washed 3 times with cold pentane to afford black crystals (30 mg, 22% yield).

II : ^{1}**H NMR** (400 MHz, C_6D_6) δ 6.30 (dd, J = 10.3, 8.0 Hz, 1H), 4.51 (tdd, J = 6.3, 2.1, 1.0 Hz, 1H), 4.39 (dd, J = 10.3, 1.1 Hz, 1H), 3.92 (ddd, J = 8.1, 2.2, 1.1 Hz, 1H), 2.47 (d, J = 6.3 Hz, 2H), 2.22 (d, J = 0.8 Hz, 3H), 1.89 (s, 15H), 1.69 (s, 8H), 1.56 (s, 6H). ^{13}**C NMR** (101 MHz, C_6D_6) δ 139.8, 128.9, 103.6, 90.4, 76.1, 57.3, 54.0, 49.5, 45.6, 44.1, 40.9, 34.0, 26.9. **Elemental analysis** calculated for $C_{19}H_{38}N_3Na$: C, 68.84; H, 11.55; N, 12.68, found : C, 68.11; H, 11.43: N, 12.43.

^{1}**H NMR DOSY :** In a J. Young NMR tube, approx. 4 mg of compound **II** was dissolved in C_6D_6 forming of a light-yellow clear solution. The NMR tube was shaken until complete dissolution of the base. Using TMS as an internal reference standard, we have been able to calculate a molecular weight (MWdet) of 316 g/mol with DSE model (5% error).

Characterization spectra

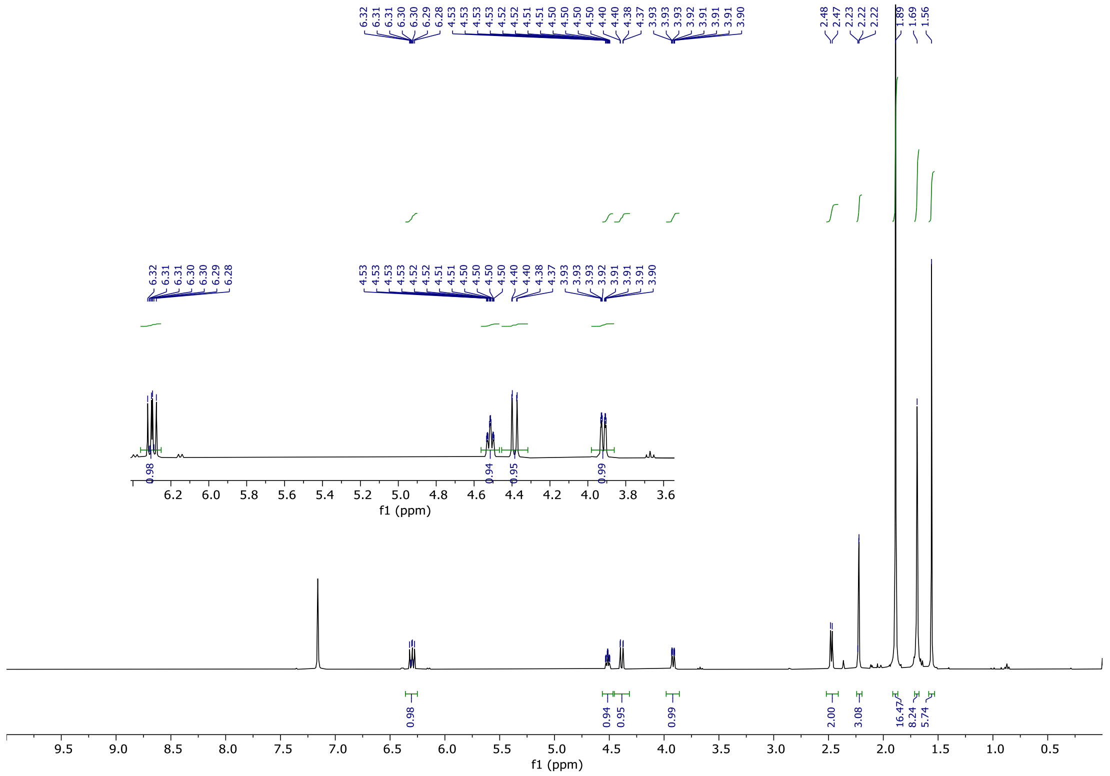

*Figure S12 : ^{1}H NMR spectrum of **II** (C_6D_6).*

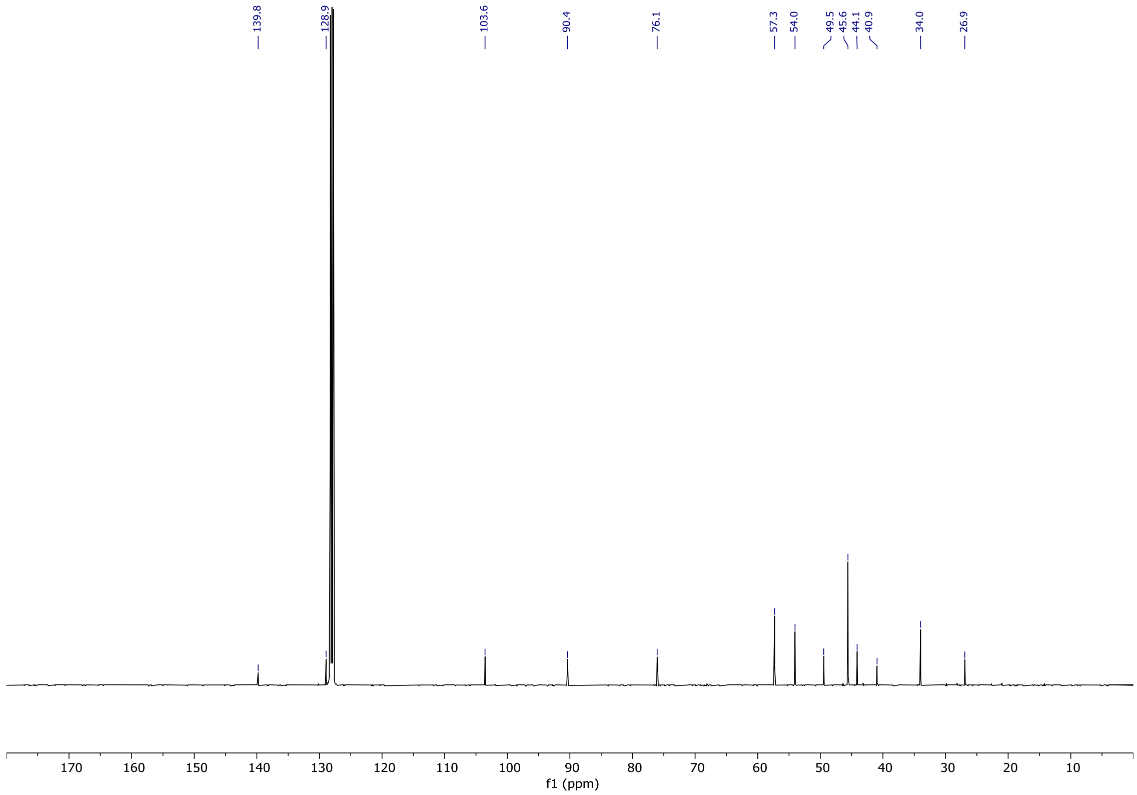

*Figure S13 : ^{13}C NMR spectrum of **II** (C_6D_6).*

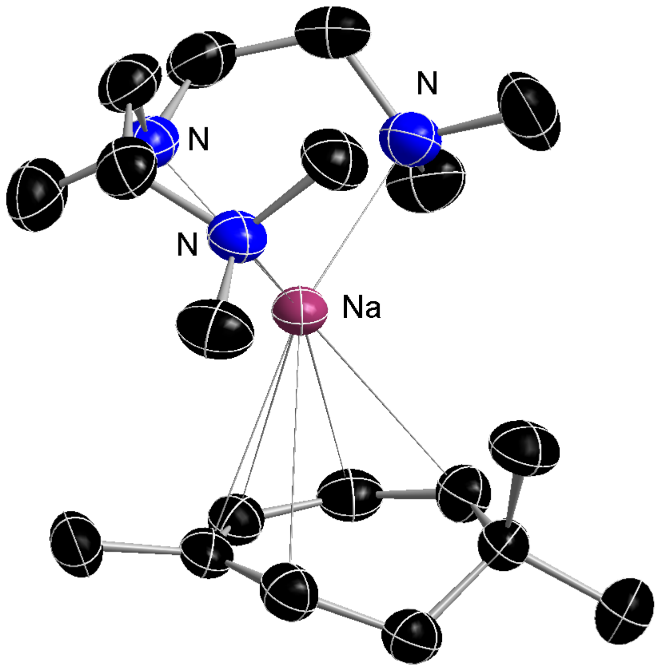

*Figure S14 : XRD structure of **II**, ellipsoids are displayed at 30 % probability and H omitted for clarity.*

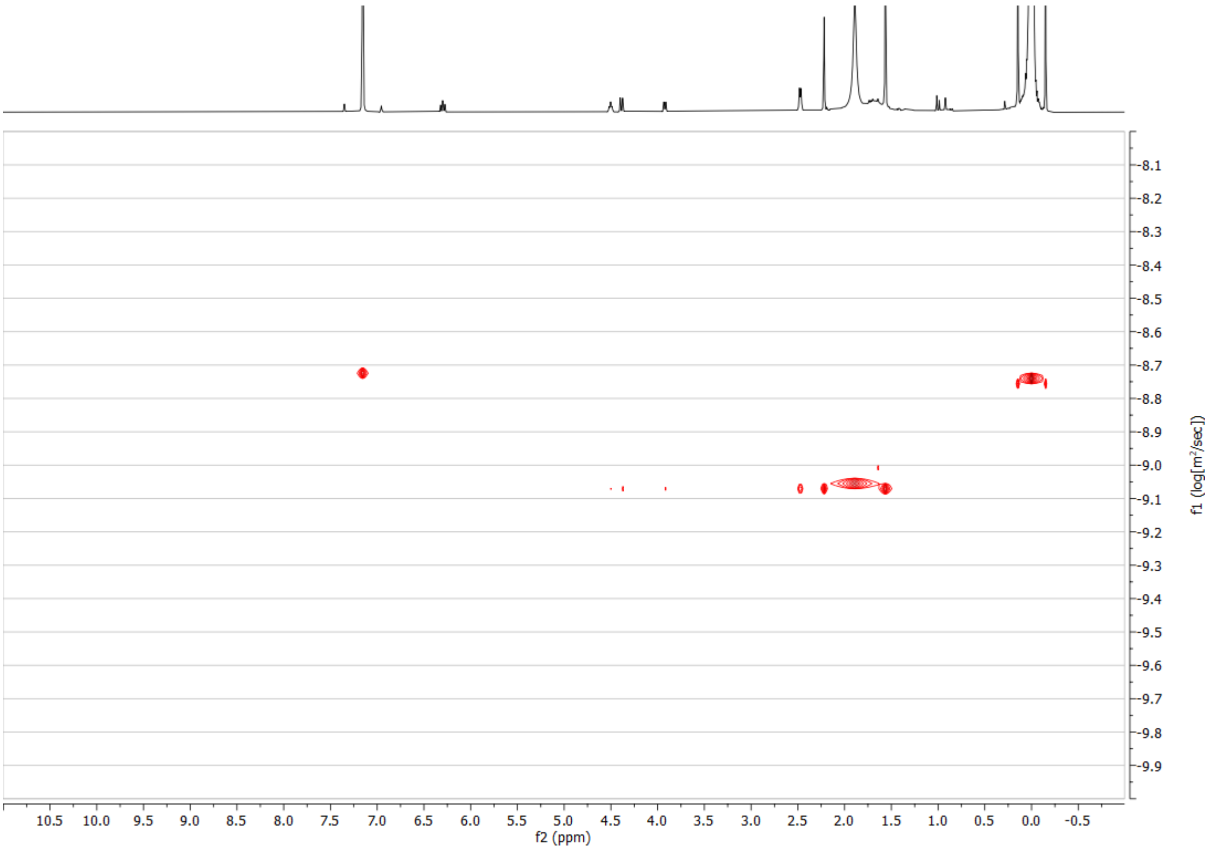

*Figure S15 : ^{1}H DOSY NMR of **II** (C_6D_6). Diffusion coefficient for TMS: 1.787 E^{-9} m^2/s, II : 8.754 E^{-10} m^2/s. MW estimated = 316 g·mol⁻¹.*

X-Ray Crystallographic Data

Compound	I	II	6
CCDC Number	2530007	2530008	2530009
Empirical formula	$C_{19}H_{38}N_3Li$	$C_{19}H_{38}N_3Na$	$C_{16}H_{16}N_4$
Mol. Mass	315.46	331.51	264.33
Temperature/K	250	250	250
Crystal system	Orthorhombic	Triclinic	Triclinic
Space group	$Pca2_1$	P-1	P-1
a/Å	10.9099(5)	8.6868(4)	8.3761(5)
b/Å	13.3024(4)	16.5361(9)	9.0920(5)
c/Å	14.9021(5)	16.6089(9)	9.5598(6)
α/°	90	112.201(4)	83.313(5)
β/°	90	95.207(4)	86.770(5)
γ/°	90	92.235(4)	80.195(5)
$V/Å^3$	2162.71(14)	2192.8(2)	712.02(7)
Z	4	4	2
l/Å	1.54186	1.54186	1.54186
2Θ range for data collection/°	6.644 to 134.818	5.79 to 134.536	9.322 to 133.618
$\rho_{calc}g/cm^3$	0.969	1.004	1.233
μ/mm^{-1}	0.418	0.619	0.601
F(000)	704.0	736.0	280.0
Crystal size/mm^3	0.3 x 0.22 x 0.09	0.21 x 0.12 x 0.04	0.77 x 0.417 x 0.07
Reflection collected	19373	43332	12811
Unique reflections	3786	7583	2425
*R*int	0.0390	0.0730	0.0233
Goof	0.948	1.011	1.085
Final R indexes [I>=2σ (I)]	R_1=0.0934, wR_2=0.2129	R_1=0.0998, wR_2=0.2364	R_1=0.0477, wR_2=0.1275
Final R indexes [all data]	R_1=0.1049, wR_2=0.2220	R_1 = 0.1531, wR_2=0.2779	R_1=0.0498, wR_2=0.1294
Largest diff. peak/hole / e $Å^{-3}$	0.23/-0.17	0.33/-0.19	0.24/-0.27

Stoichiometric electrocyclization of β-ocimene

General procedure

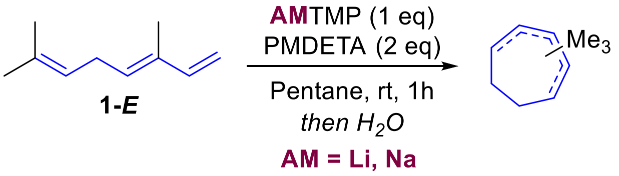

GPI : In an oven-dried 30 mL Schlenk flask equipped with a magnetic stirrer and placed in a glovebox, pentane (8 mL), PMDETA (1.84 mL, 8.82 mmol, 2 equiv), and **1-*E*** (>95 % pure, 0.751 mL, 4.41 mmol, 1 equiv) were added. AMTMP (1 equiv) was then added in one portion, the schlenk was closed, removed from the glovebox, and stirred for 1h at room temperature. The resulting mixture was then quenched with H_2O, then extracted with 3x pentane (3x5mL) and washed with HCl (2x5mL). The combined organic layer was filtrated on a silica pad, dried over $MgSO_4$ and slowly evaporated under vacuum (volatile product, 350 mbar for 1 h at 45 °C).

LiTMP electrocyclization

Procedure

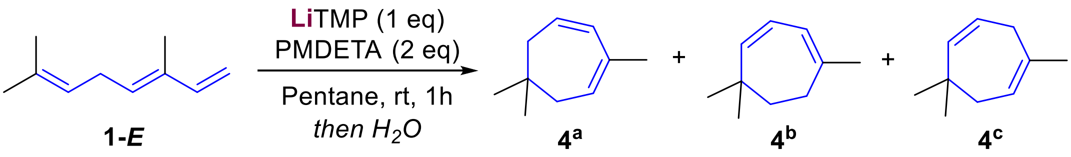

According to **GPI** using LiTMP (648 mg, 4.41 mmol, 1 equiv) in pentane. Reaction afforded a 1:1:1 mixture of **4**$^{a\text{-}c}$ isomers (504 mg, 3.70 mmol) as a colourless oil in 84 % yield.

2,6,6-trimethylcyclohepta-1,3-diene (4^{a}): ^{1}H NMR (500 MHz, $CDCl_3$) δ 5.86 (dt, J = 11.4, 5.7 Hz, 1H), 5.83 – 5.78 (m, 1H), 5.67 (t, J = 6.4 Hz, 1H), 1.92 (d, J = 6.0 Hz, 2H), 1.85 (d, J = 6.0 Hz, 2H), 1.78 (d, J = 1.4 Hz, 3H), 0.94 (d, J = 1.4 Hz, 6H). **^{13}C NMR** (126 MHz, $CDCl_3$) δ 134.9, 131.6, 131.4, 127.0, 43.4, 42.3 (2C), 29.2, 24.1. **HRMS (EI)** m/z: $[M^{\bullet}]^+$ Calcd for $C_{10}H_{16}$ 136.12465. Found 136.12478.

1,5,5-trimethylcyclohepta-1,3-diene (4^{b}): ^{1}H NMR (500 MHz, $CDCl_3$) δ 5.57 – 5.42 (m, 3H), 2.24 (t, J=5.9 Hz, 2H), 1.82 (s, 3H), 1.60 (t, J=5.9 Hz, 2H), 1.03 (d, J = 1.1 Hz, 6H). **^{13}C NMR** (126 MHz, $CDCl_3$) δ 143.5, 141.6, 121.2, 120.4, 38.2, 37.5, 32.1, 30.2, 27.0. **HRMS (EI)** m/z: $[M^{\bullet}]^+$ Calcd for $C_{10}H_{16}$ 136.12465. Found 136.12485.

2,6,6-trimethylcyclohepta-1,4-diene (4^{c}) : ^{1}H NMR (500 MHz, $CDCl_3$) δ 5.49 (t, J = 7.0 Hz, 1H), 5.46 – 5.33 (m, 1H), 5.28 (d, J = 11.8 Hz, 1H), 2.70 (d, J = 5.3 Hz, 1H), 2.14 (d, J = 7.0 Hz, 1H), 1.74 (s, 1H), 0.94 (s, 6H). **^{13}C NMR** (126 MHz, $CDCl_3$) δ 141.2, 140.1, 123.1, 122.9, 39.3, 35.3, 32.7, 30.1, 25.4. **HRMS (EI)** m/z: $[M^{\bullet}]^+$ Calcd for $C_{10}H_{16}$ 136.12465. Found 136.12481.

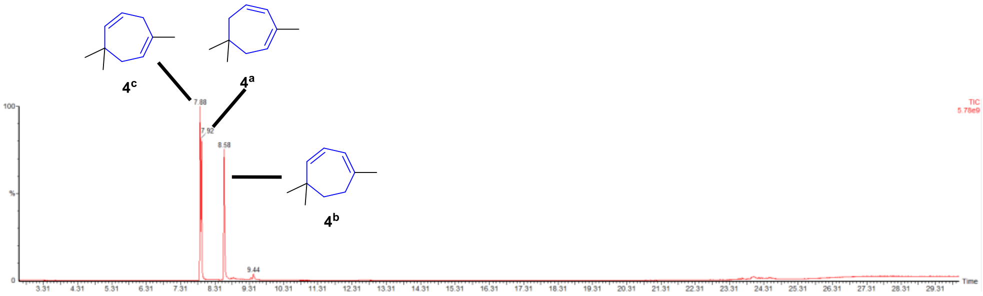

*Figure S16 : GC-MS chromatogram of **4**[a-c] mixture.*

Characterization spectrum

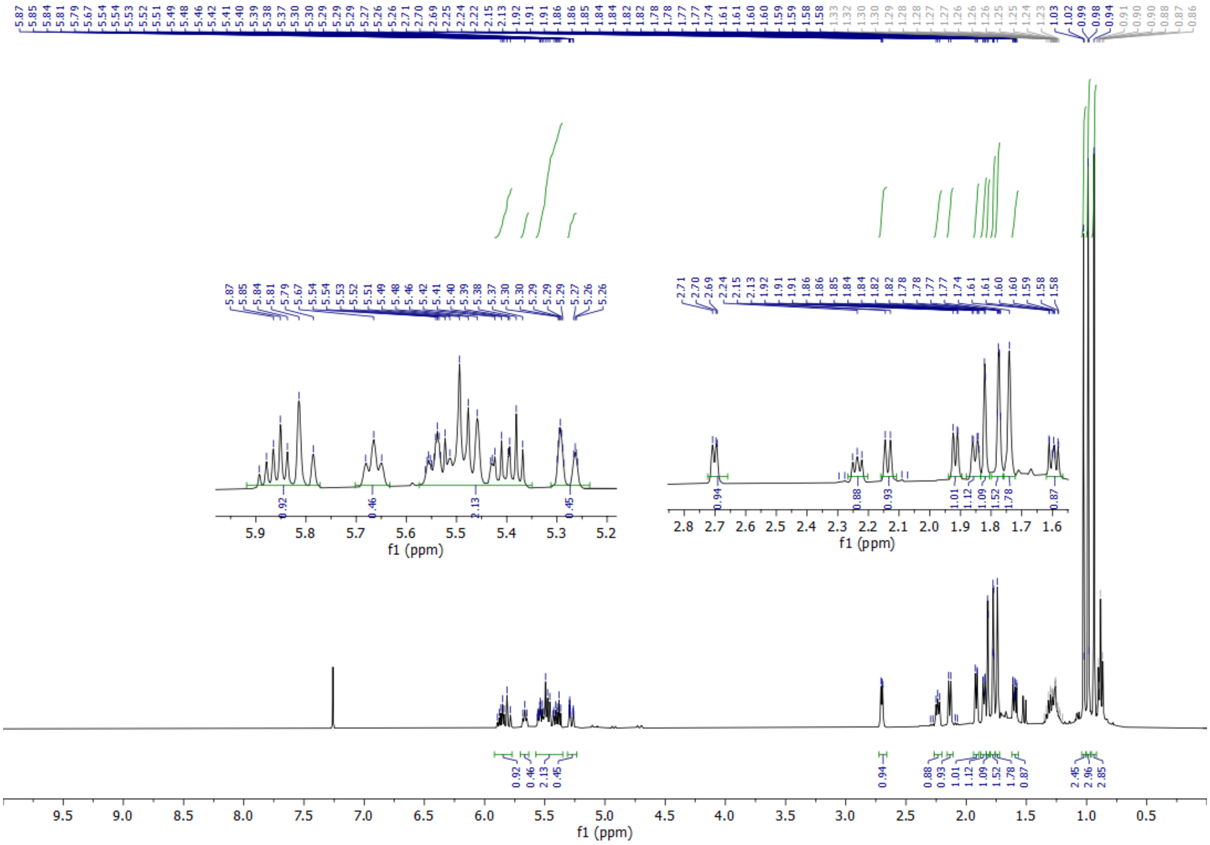

*Figure S17 : ^{1}H NMR spectrum of **4**[a-c] mixture ($CDCl_3$).*

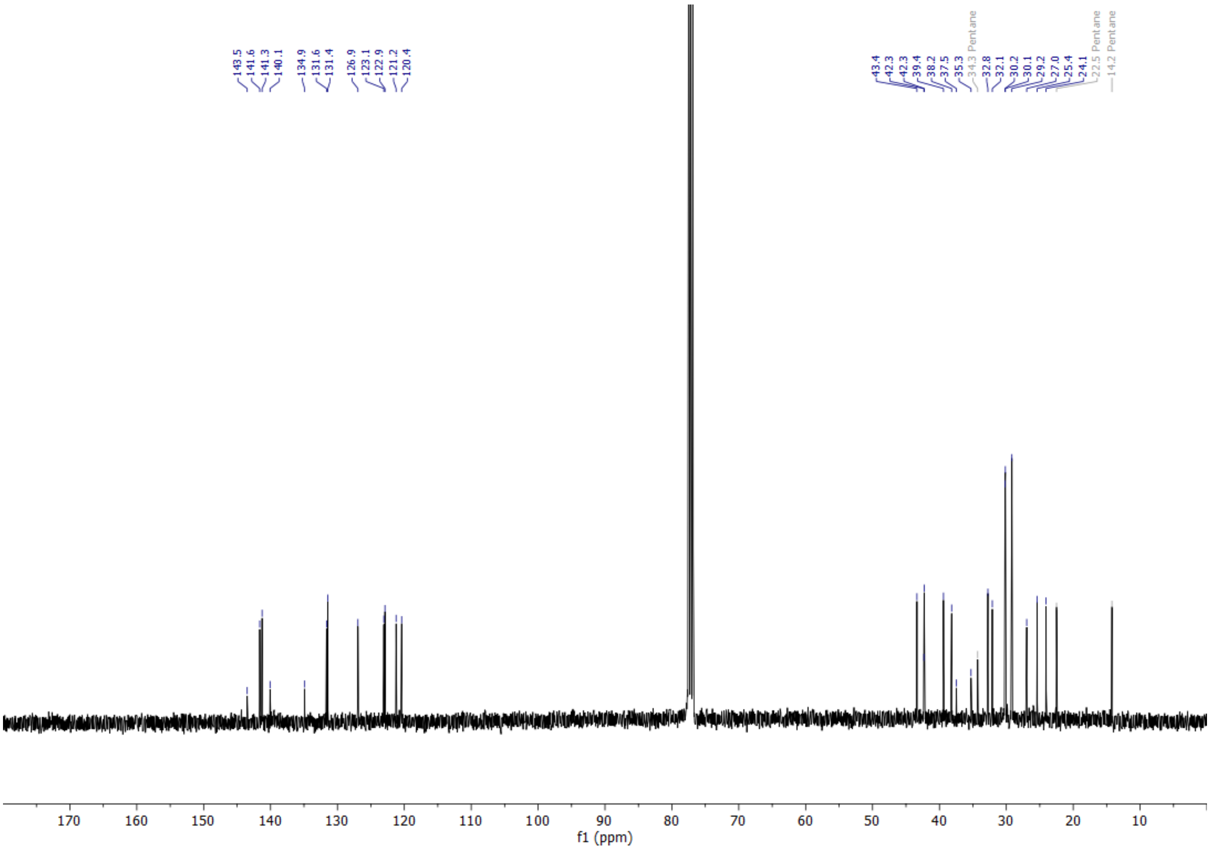

*Figure S18 : ^{13}C NMR spectrum of **4**$^{a\text{-}c}$ mixture (CDCl$_3$).*

Prep-GC isolation

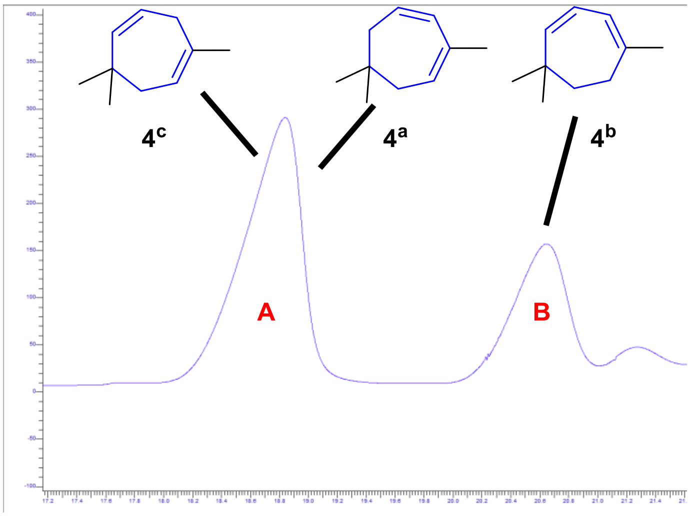

*Figure S19 : GC-TCD chromatogram of **4**$^{a\text{-}c}$ mixture (packed column).*

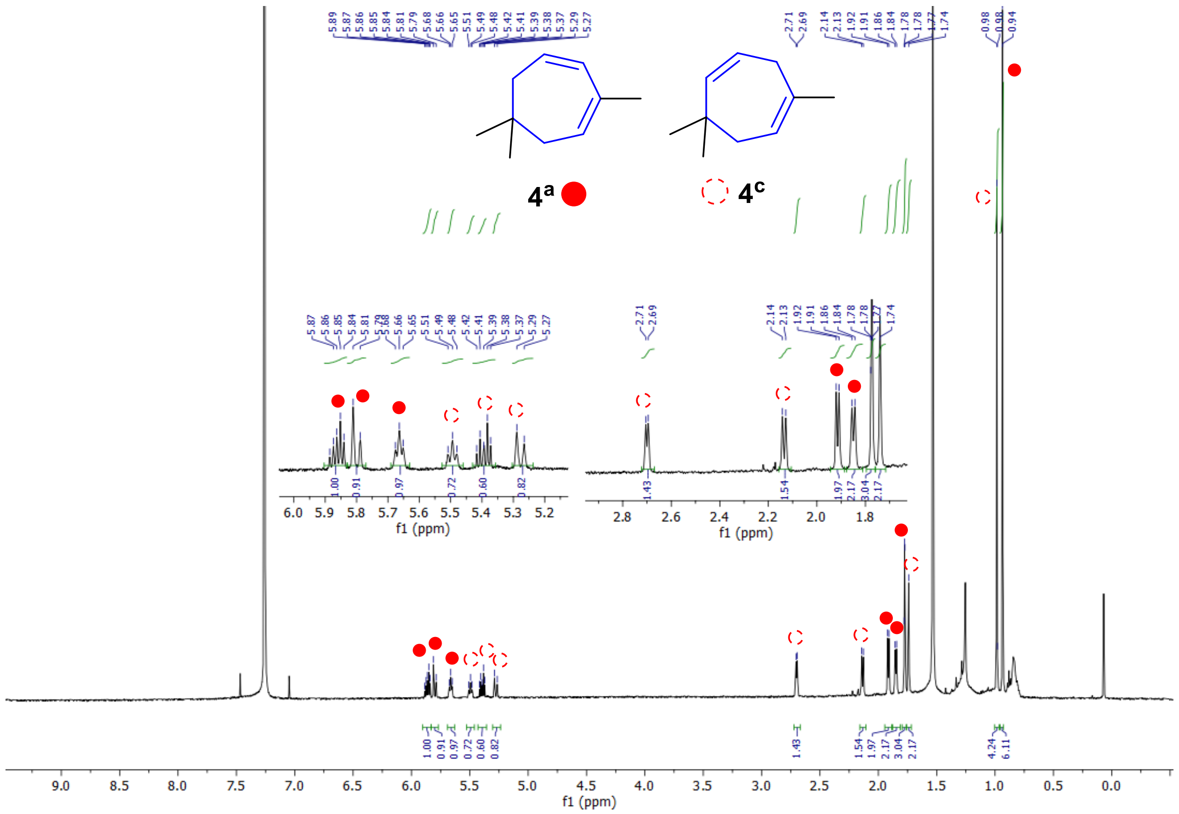

Figure S20 : ^{1}H NMR spectrum of semi prep-GC isolated peak A ($CDCl_3$).

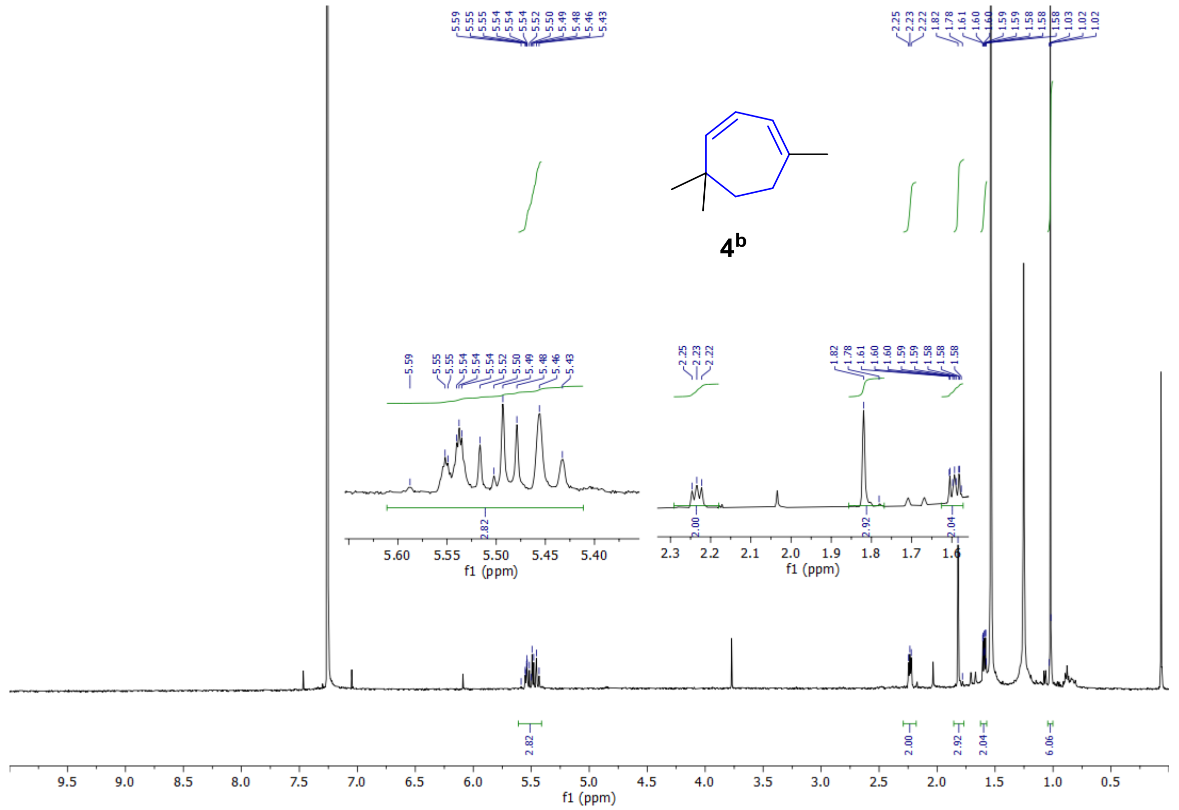

Figure S21 : ^{1}H NMR spectrum of semi prep-GC isolated peak B ($CDCl_3$).

NaTMP electrocyclization

Procedure

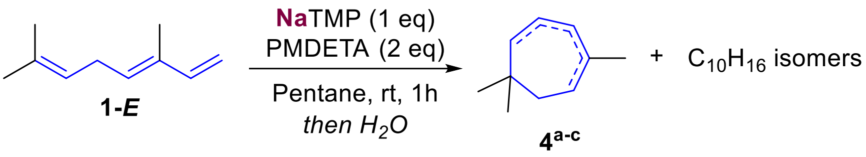

According to **GPI** using NaTMP (831 mg, 5 mmol, 1 equiv) in pentane. Reaction afforded a mixture of $C_{10}H_{16}$ isomers (527 mg, 3.87 mmol) as a colourless oil in 77 % yield.

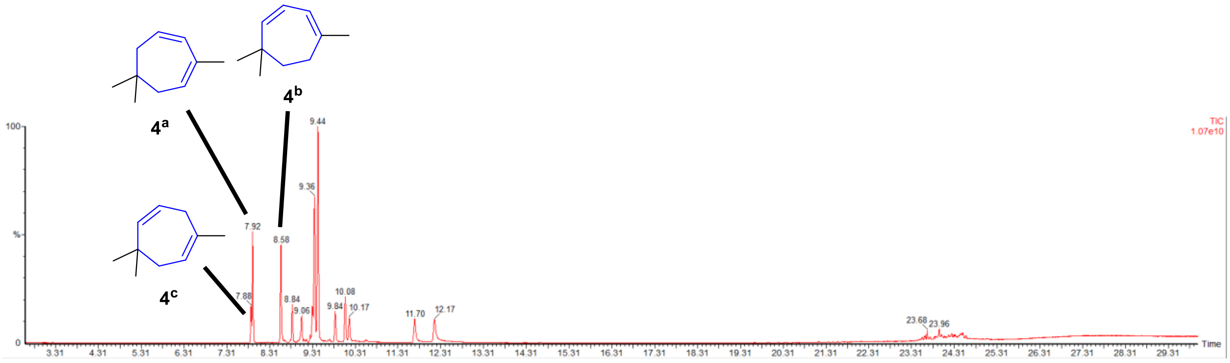

Figure S22 : GC-MS chromatogram of $C_{10}H_{16}$ mixture obtained from NaTMP cyclization (all peaks between 7.31 to 13 min have the same m/z ratio corresponding to a 136 g/mol MW).

Spectrum

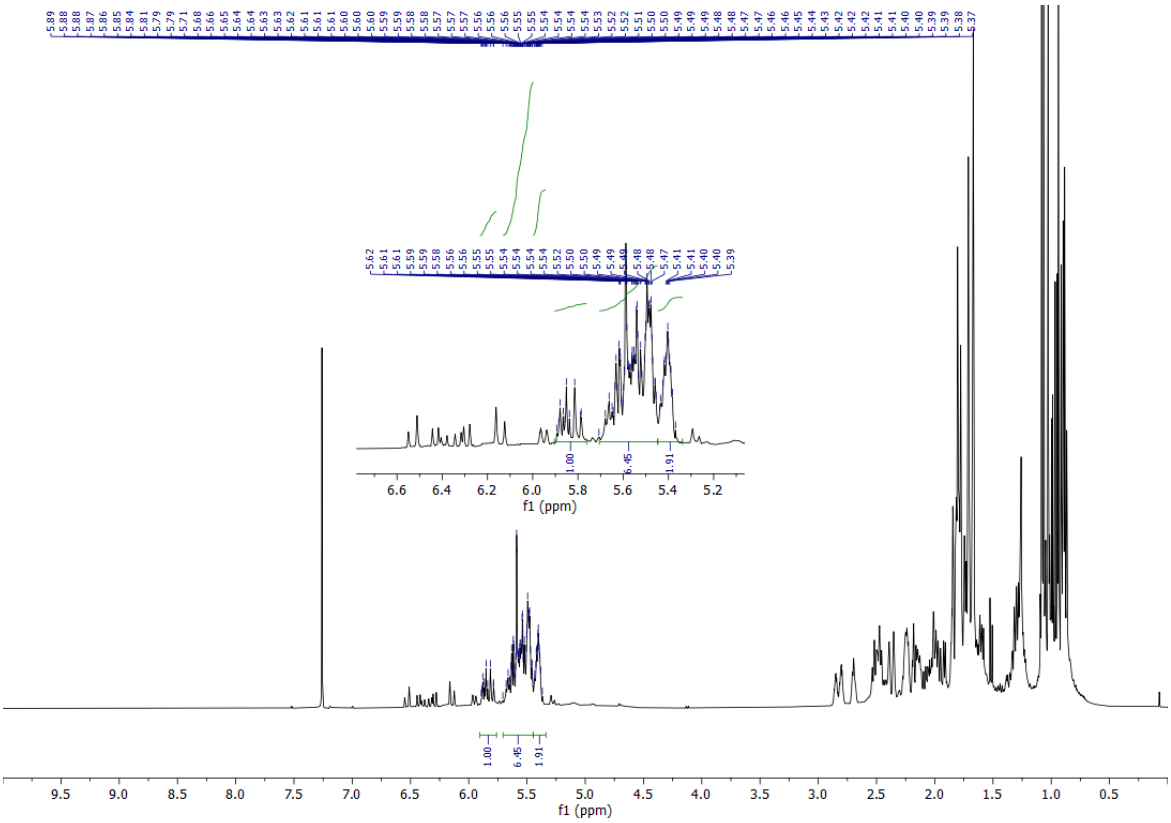

Figure S23 : ^1H NMR spectrum of $C_{10}H_{16}$ mixture ($CDCl_3$).

Catalytic electrocyclization of β-ocimene

General procedure

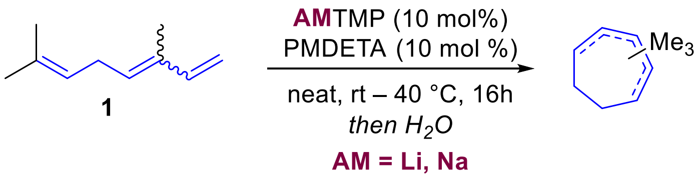

GPII (small scale): In the glovebox, in an J. Young NMR tube equipped with a closed Wilmad capillary filled with C_6D_6 was added **1** (1.5 mmol, 0.341 mL of commercial ocimene **1-*Z*** (74.4 % pure) or 0.255 mL of pure ocimene **1-*E***, 1 equiv) and PMDETA (0.15 mmol, 0.0313 mL, 10 mol%). The tube was removed from the glovebox, and a blank ^{1}H NMR spectrum was acquired. The tube was then returned to the glovebox, and AMTMP (10 mol%) was added in a single portion. The sample was subsequently removed from the glovebox, maintained at the specified temperature, and the reaction progress was monitored by ^{1}H NMR spectroscopy. After completion of the reaction, the NMR tube was opened and quenched with two drops of deionized water. The tube was then extracted with pentane (3 ×), dried over $MgSO_4$, and filtered through a short silica pad. The solvent was slowly removed under reduced pressure (350 mbar, 1 h, 45 °C) to afford the seven-membered cycles.

GPIII (big scale): In the glovebox, in an ampoule equipped with a magnetic stirrer was added β-ocimene (5 mmol, 1.15 mL of commercial ocimene **1-*Z*** (74.4 % pure) or 0.852 mL of pure ocimene **1-*E***, 1 equiv) and PMDETA (0.5 mmol, 0.104 mL, 10 mol%). AMTMP (10 mol%) was added in a single portion. The ampoule was subsequently removed from the glovebox, maintained at the specified temperature, and the reaction progress was monitored by ^{1}H and NMR spectroscopy. After completion of the reaction, the ampoule was opened and quenched with 2 mL of deionized water. The mixture tube was washed with 1M HCl (2x10 mL) then extracted with pentane (3 × 15 mL). The combined organic layers were dried over $MgSO_4$ and filtered through a short silica pad. The solvent was slowly removed under reduced pressure (350 mbar, 1 h, 45 °C) to afford the seven-membered cycles.

LiTMP electrocyclization

Procedure

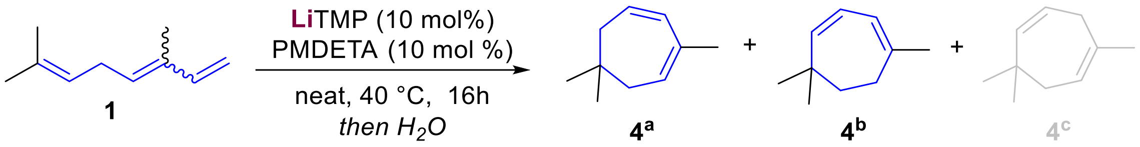

According to **GPII** using LiTMP (22.08 mg, 0.15 mmol, 10 mol%). Optimization trials were done with commercial ocimene **1-*Z***, and an isolation attempt (Table 1, entry 7) was performed with pure ocimene **1-*E***. The latter reaction afforded a 1:1 mixture of **4**[a] and **4**[b] isomers (124 mg, 0.91 mmol) as a colourless oil in 61 % yield. NMR data of products are reported in **page 20**.

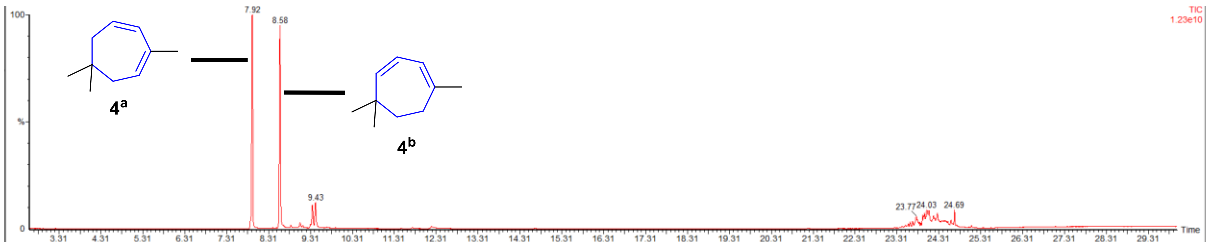

*Figure S24 : GC-MS chromatogram of **4**[a] and **4**[b] mixture.*

Characterization spectrum

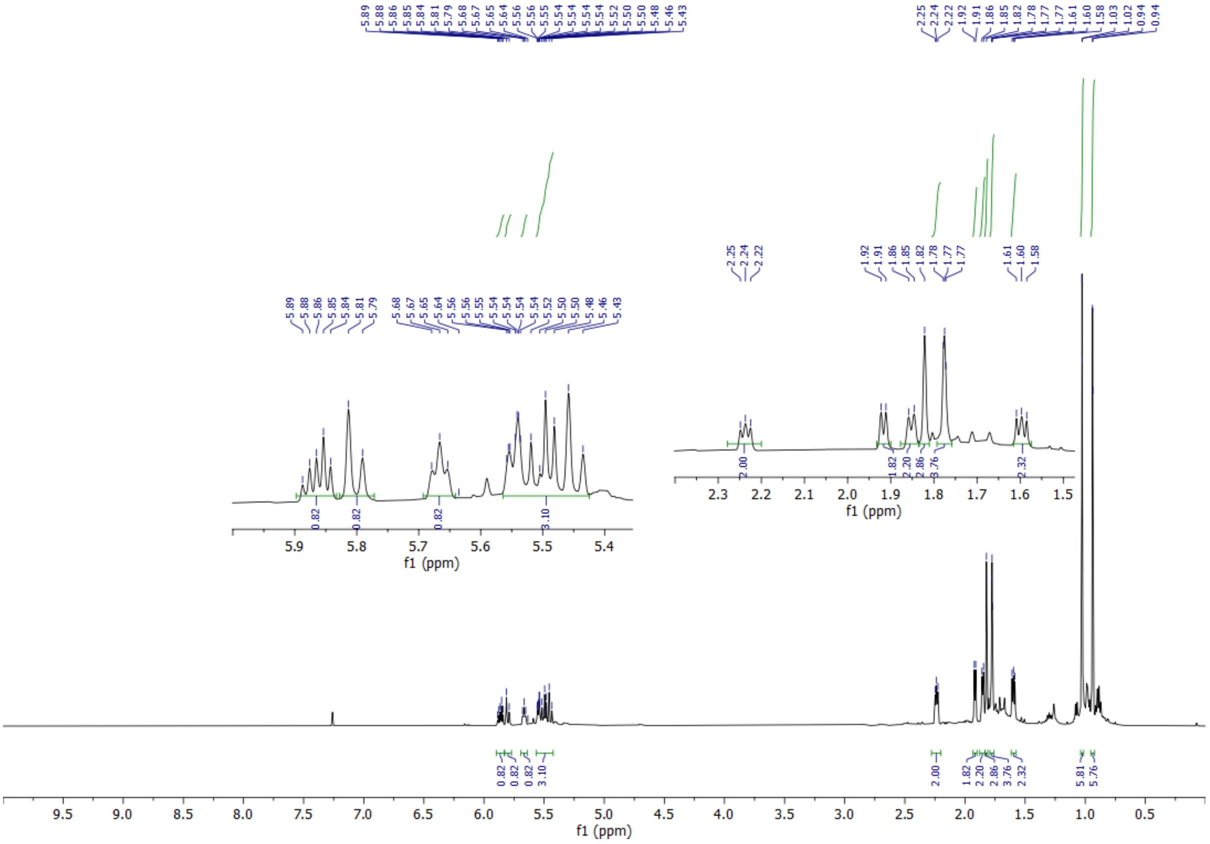

*Figure S25 : ^{1}H NMR spectrum of **4**[a] **/ 4**[b] mixture ($CDCl_3$).*

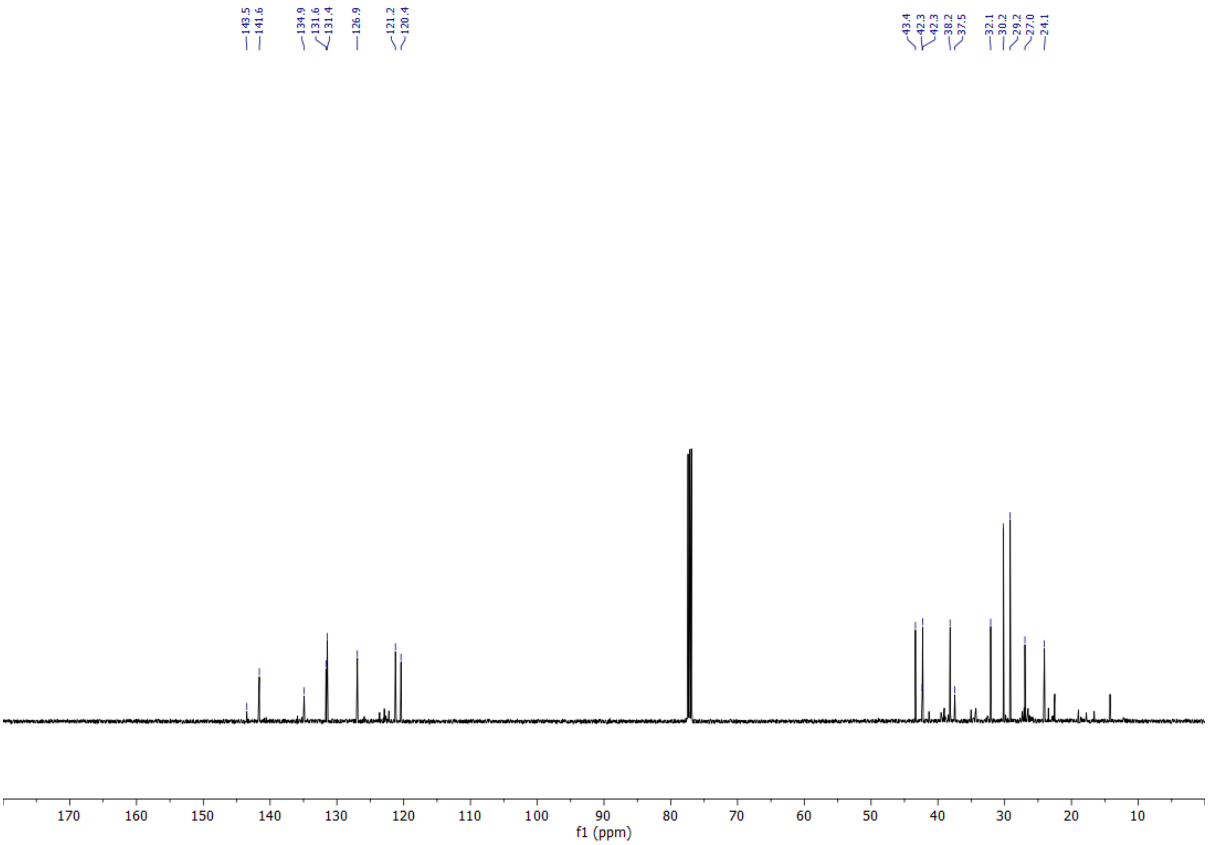

*Figure S26 : ^{13}C NMR spectrum of **4a / 4b** mixture (CDCl$_3$).*

In-situ monitoring of the reaction with LiTMP

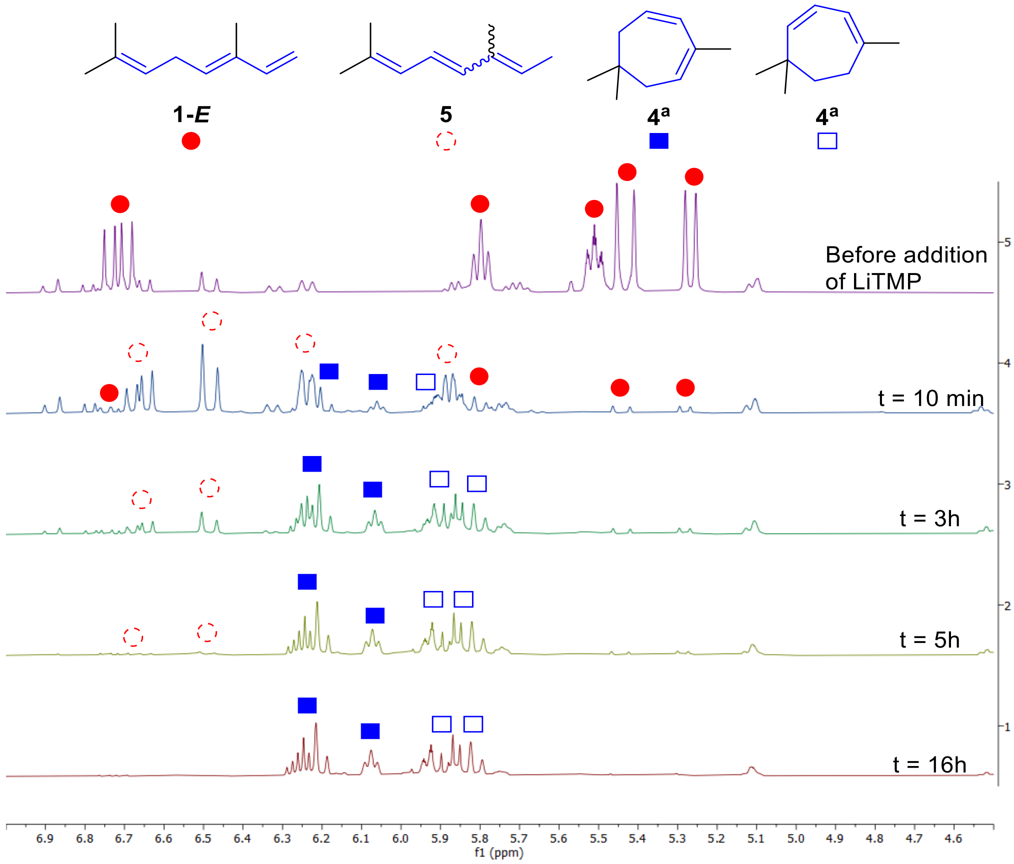

Figure S27 : ^{1}H NMR spectrum of in-situ catalytic electrocyclization with LiTMP and pure 1-E (C$_6$D$_6$).

In-situ monitoring of the reaction with I

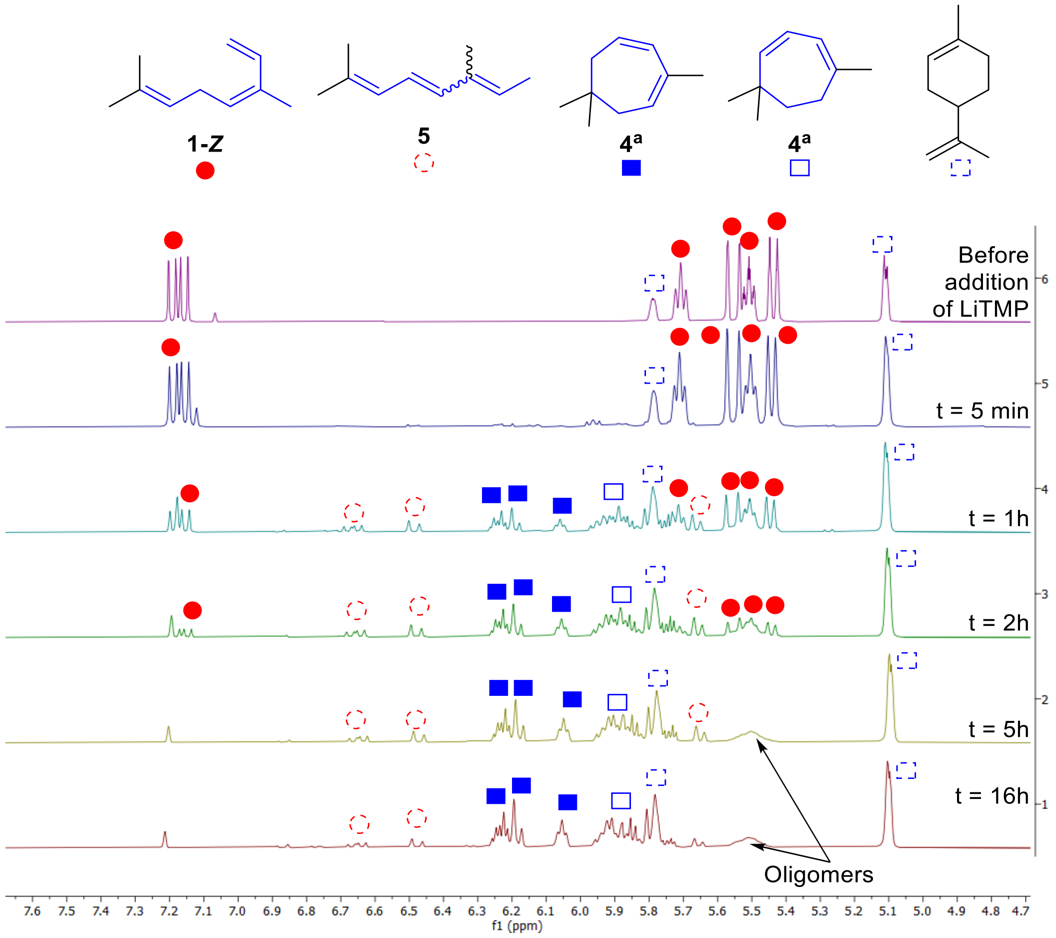

Figure S28 : ^{1}H NMR spectrum of in-situ catalytic electrocyclization with I and commercial 1-Z (C_6D_6).

NaTMP electrocyclization

Procedure

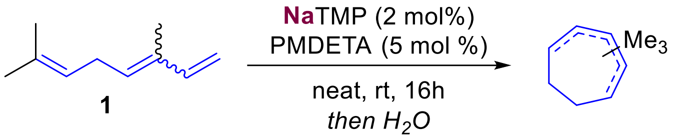

According to **GPII** using NaTMP (4.9 mg, 0.03 mmol, 2 mol%) and PMDETA (15.7 µL, 0.075 mmol, 5 mol%) with commercial **1-*Z*** or pure **1-*E***. The latter reaction afforded a mixture of $C_{10}H_{16}$ isomers (155 mg, 1.14 mmol) as a colourless oil in 76 % yield. GC-MS chromatogram and NMR spectrum of the crude are reported below.

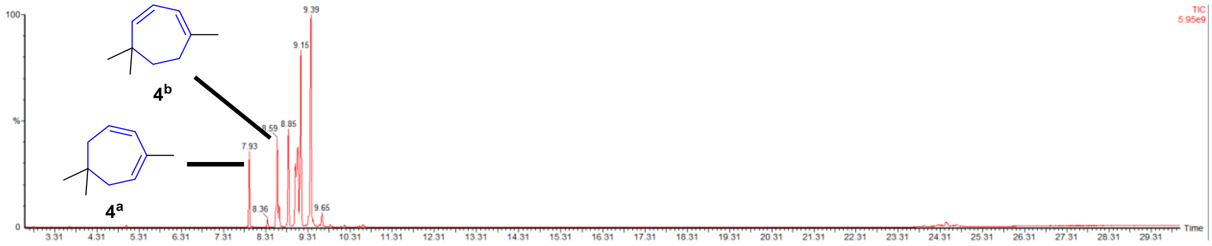

*Figure S29 : GC-MS chromatogram of **$C_{10}H_{16}$** mixture.*

Spectrum

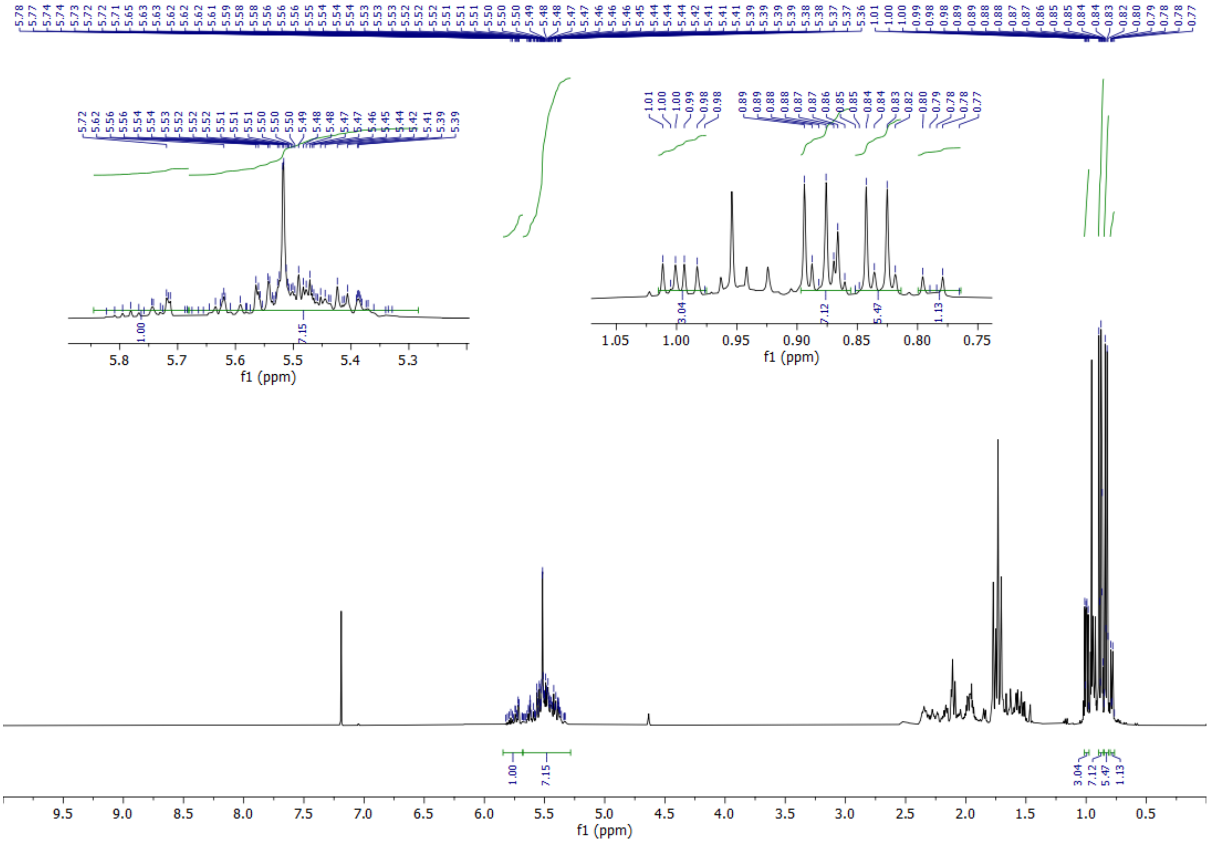

Figure S30 : ^{1}H NMR spectrum of $C_{10}H_{16}$ mixture ($CDCl_3$).

Derivatization of $C_{10}H_{16}$ isomers from NaTMP catalytic electrocyclization

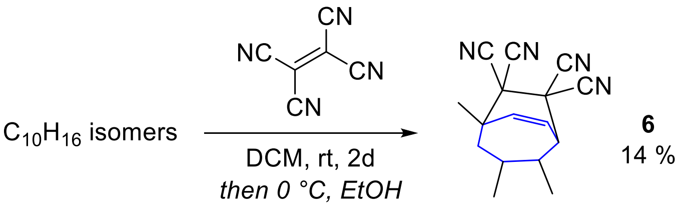

According to a reported procedure [11], in a 100 mL flask equipped with a stirrer under Ar atmosphere was added 37.5 mL of dry DCM and $C_{10}H_{16}$ isomer mixture (272 mg, 2.0 mmol overall, 1 eq). The crude was then degassed via freeze-pump-thaw technique 3 times and TCNE (256 mg, 2.0 mmol, 1 equiv) was then quickly added to the flask. The reaction was allowed to stir at room temperature for 2 days. Then solvent was removed *in vacuo* and crude dissolved in the minimum amount of hot EtOH for dissolution and then stored in the fridge at 4°C. After two days, the solids have been filtered and washed with cold EtOH to afford **6** as pure white crystalline solids (72 mg, 0.27 mmol) in 14 % yield.

^{1}H NMR (400 MHz, $CDCl_3$) δ 6.33 (dd, J = 9.2, 7.7 Hz, 1H), 6.17 (d, J = 9.2 Hz, 1H), 3.43 (t, J = 7.2 Hz, 1H), 2.50 (dqd, J = 12.8, 6.4, 5.0 Hz, 1H), 2.34 (h, J = 6.8 Hz, 1H), 1.78 – 1.68 (m, 1H), 1.65 (s, 3H), 1.61 – 1.49 (m, 1H), 0.97 (d, J = 6.4 Hz, 3H), 0.89 (d, J = 7.1 Hz, 3H).

^{13}C NMR (101 MHz, $CDCl_3$) δ 138.1, 130.1, 113.5, 112.3, 111.2, 111.1, 49.5, 48.1, 44.6, 44.3, 38.6, 33.4, 31.7, 28.1, 19.7, 11.9. **HRMS (APCI)** m/z: $[M+MeOH+H]^+$ Calcd for $C_{17}H_{21}ON_4$ 297.17099. Found 297.17156.

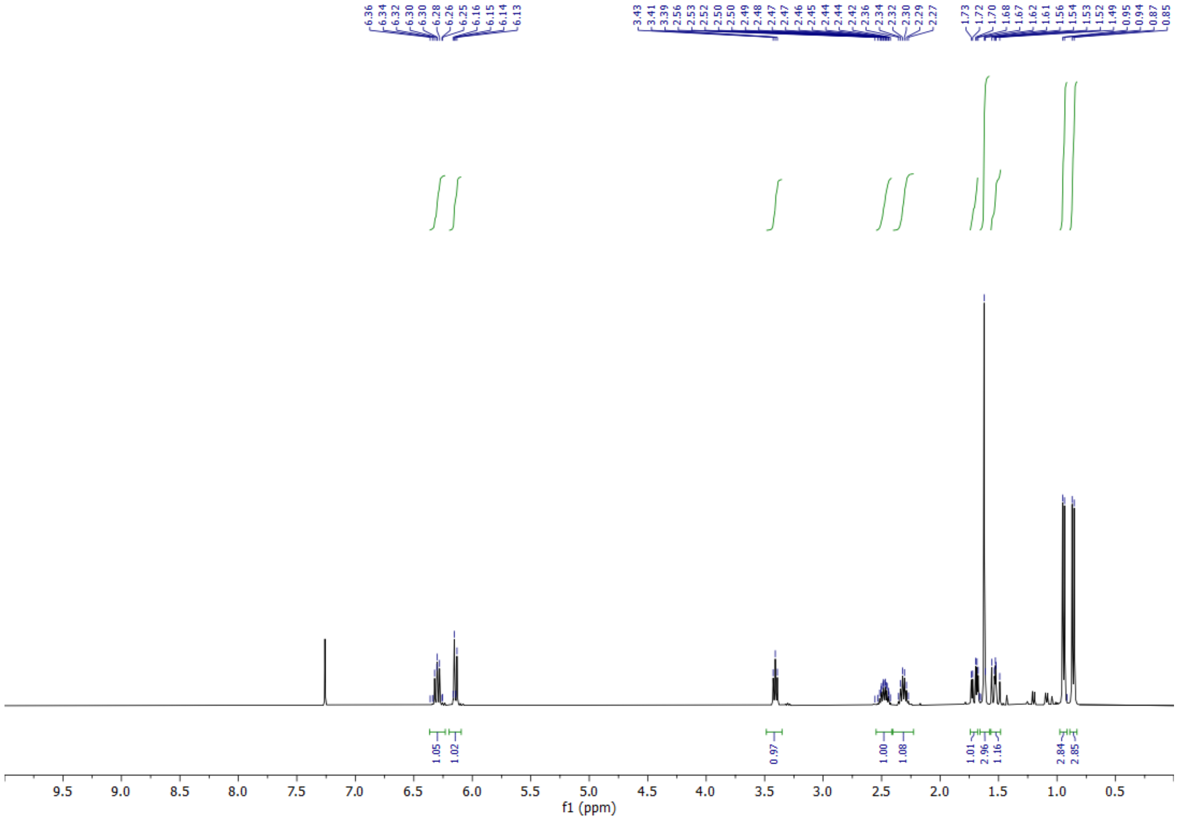

*Figure S31 : ^{1}H NMR spectrum of **6** ($CDCl_3$).*

[11] C. Rücker, D. Lang, J. Sauer, H. Friege, R. Sustmann. *Chem. Ber.* **1980**, *113* (5), 1663–1690.

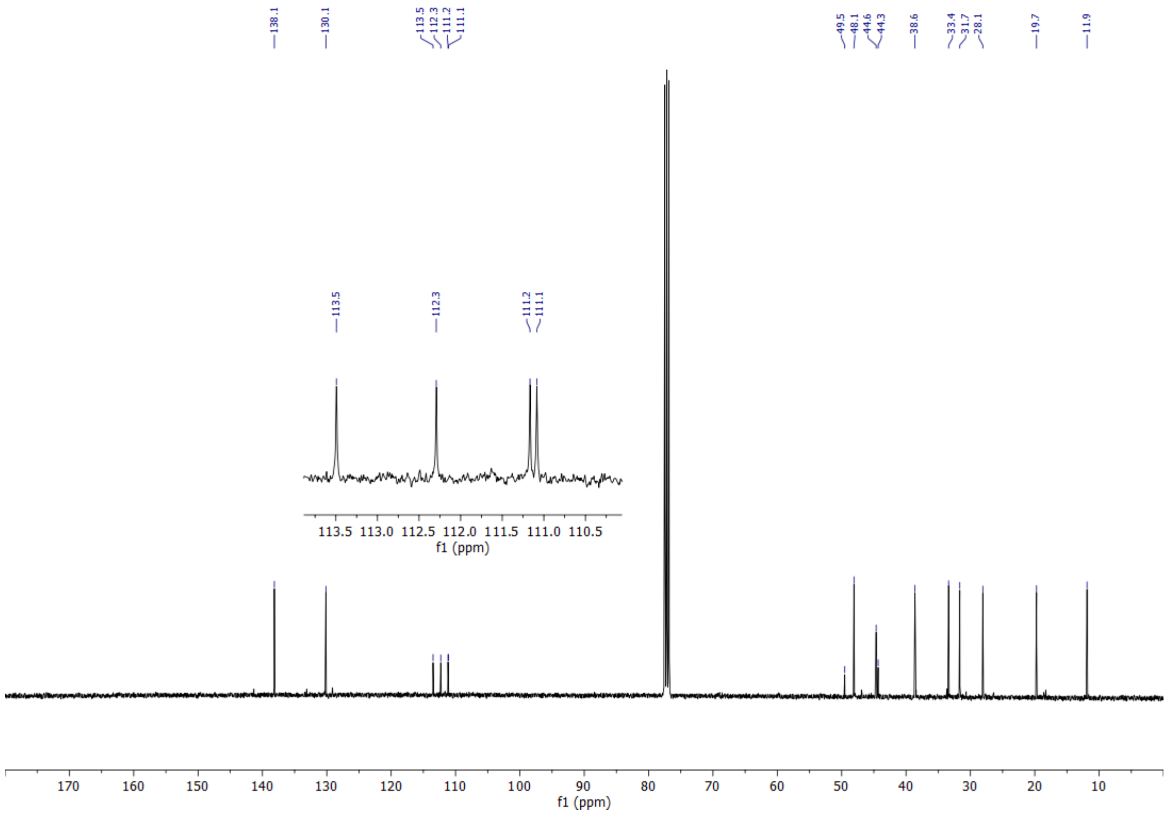

*Figure S32 : ^{13}C NMR spectrum of **6** ($CDCl_3$).*

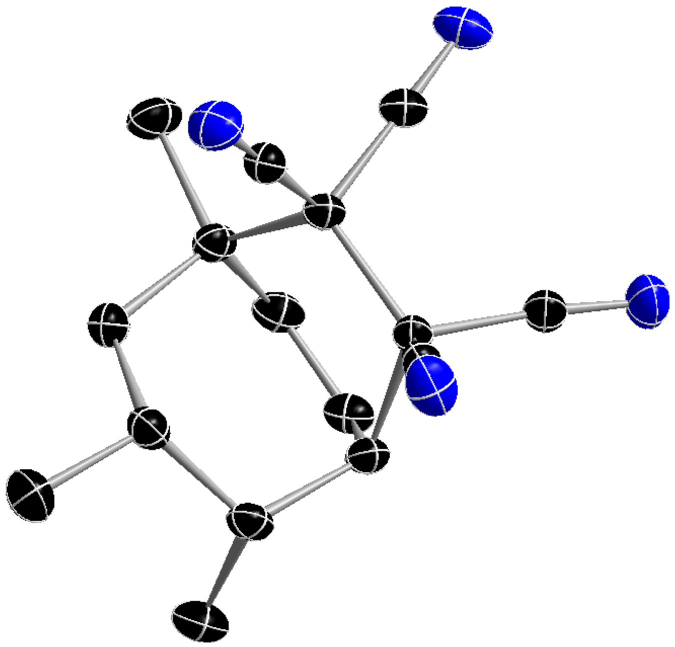

*Figure S33 : XRD structure of **6**, ellipsoids are displayed at 50 % probability and H has been omitted for clarity.*

Scale up and hydrogenation

Pure 1-*E*

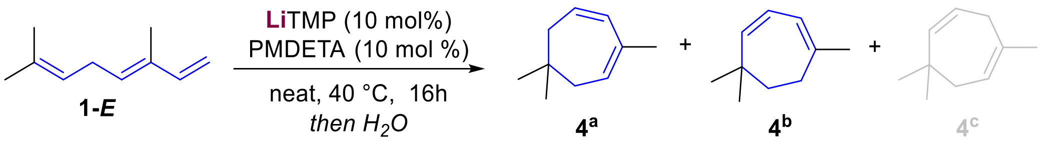

According to **GPIII** using LiTMP (73.6 mg, 0.5 mmol, 10 mol%) along with 95 % pure **1-*E***. The latter reaction afforded a 1:1 mixture of **4**^{a} and **4**^{b} isomers (571 mg, 4.2 mmol, 84 % yield) as a colourless oil. NMR data of products are reported in **page 20**.

Hydrogenation procedure

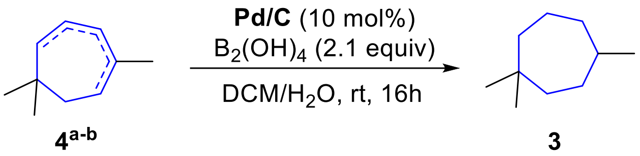

According to a reported procedure,[12] in a round-bottom flask equipped with a stirrer was introduced $B_2(OH)_4$ (753 mg, 8.4 mmol, 2.1 equiv) and Pd/C (40.0 mg, 0.038 mmol, 10 mol%). The round bottom flask was then purged 3 times with argon. Dry DCM (10 mL) was then added followed by **4**$^{a\text{-}b}$ (545 mg, 4.0 mmol, 1 equiv). Then, 0.5 mL of deionized water was added. The reaction was stirred overnight at room temperature. After completion of the reaction (monitored by GC-MS), the mixture was filtrated through one celite pad. The solvent was then removed under vacuum and the mixture was then filtrated with pentane on one silica pad and evaporated to afford **3** as a colourless liquid (448 mg, 3.2 mmol, 80 % yield).

^{1}H NMR (400 MHz, $CDCl_3$) δ 1.75 (m, 1H), 1.61 – 1.04 (m, 9H), 1.02 – 0.64 (m, 18H). **^{13}C NMR** (101 MHz, $CDCl_3$) δ 42.4, 40.4, 39.9, 36.9, 33.4, 31.7, 31.2, 30.6, 24.1, 22.3.
Data agrees with those previously reported in the literature.[13]

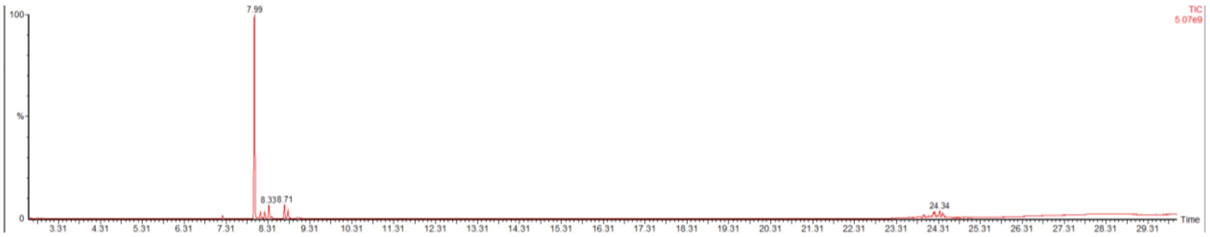

*Figure S34 : GC-MS chromatogram of **3**.*

[12] S. P. Cummings, T.-N. Le, G. E. Fernandez, L. G. Quiambao, B. J. Stokes. *J. Am. Chem. Soc.* **2016**, *138* (19), 6107–6110.
[13] J.-D. Woodroffe, B. G. Harvey. *Energy Technol.* **2021**, *9* (8), 2100221.

Characterization spectrum

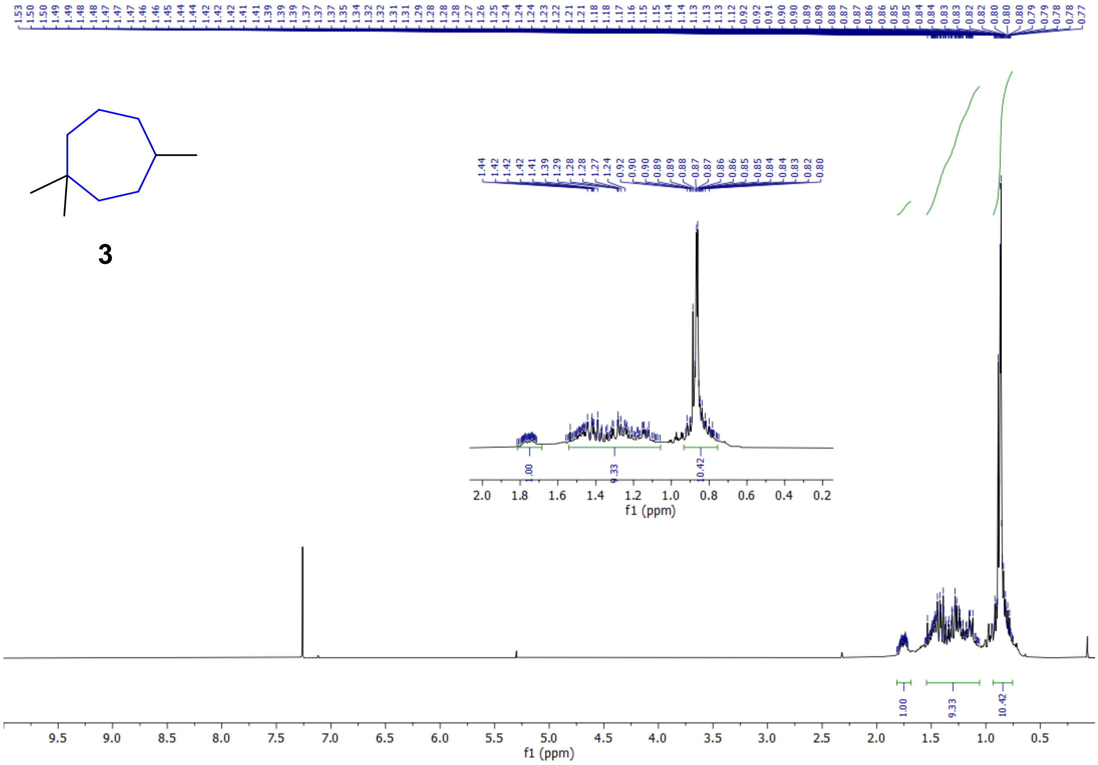

*Figure S35 : ^{1}H NMR spectrum of **3** ($CDCl_3$).*

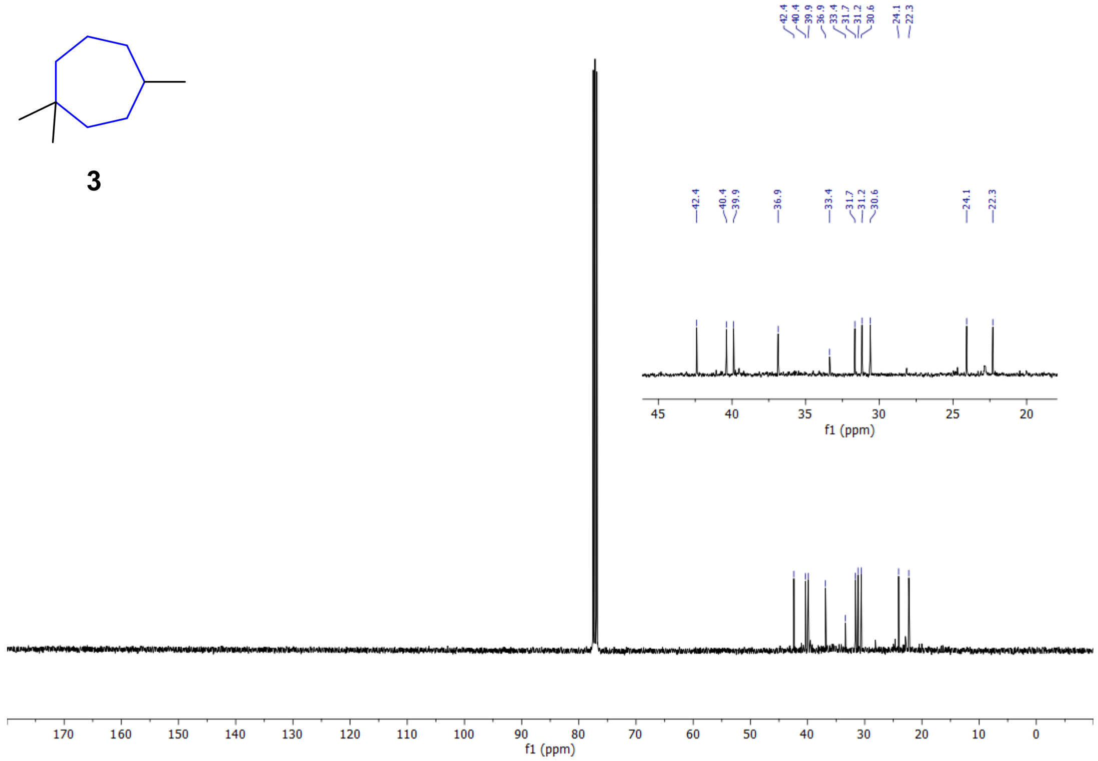

*Figure S36 : ^{13}C NMR spectrum of **3** ($CDCl_3$).*

Commercial 1-*Z*

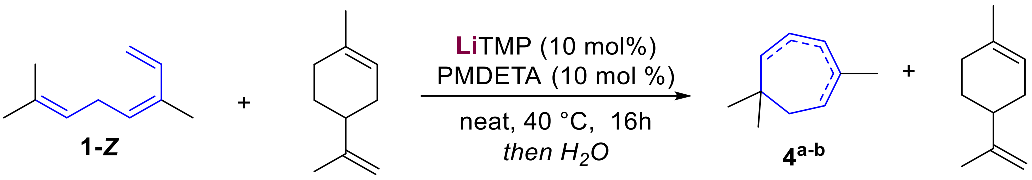

According to **GPIII** using LiTMP (1.47 g, 10 mmol, 10 mol%) along with commercial **1-*Z*** (22 mL, 18.3 g, 100 mmol of **1-*Z***) (highly exothermic reaction). The latter reaction afforded a 1:1 mixture of $\mathbf{4^a}$ and $\mathbf{4^b}$ isomers (18 g, 98 mmol of $\mathbf{4^{a\text{-}b}}$, 98 % yield) as a colourless oil.

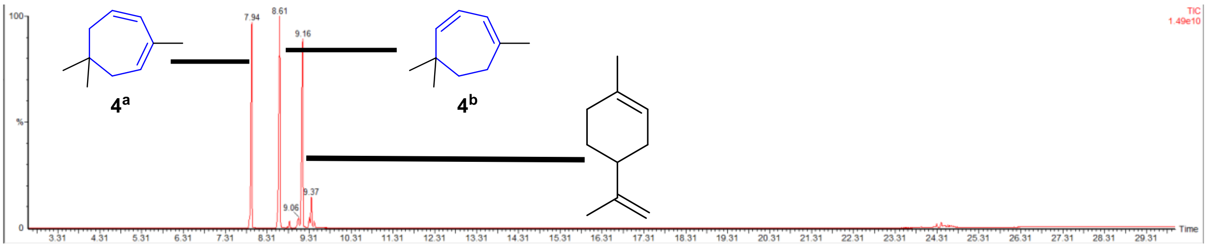

*Figure S37 : GC-MS chromatogram of $\mathbf{4^{a\text{-}b}}$ + limonene from commercial **1-Z**.*

Hydrogenation procedure

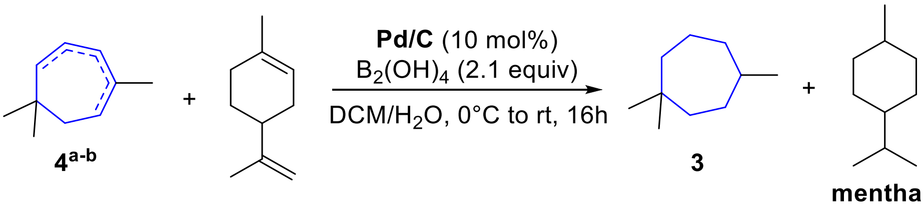

According to a reported procedure,[12] in a 500 mL round-bottom flask in an ice bath equipped with a stirrer was introduced $B_2(OH)_4$ (24.9 g, 277 mmol, 2.1 equiv) and Pd/C (1.16 g, 1.09 mmol, 10 mol%). The round bottom flask was then purged 3 times with argon. Dry DCM (280 mL) was then added followed by $\mathbf{4^{a\text{-}b}}$ + limonene (18 g, 98 mmol of $\mathbf{4^{a\text{-}b}}$ & 34 mmol of limonene, 1 equiv). Then, 14 mL of deionized water was added. The reaction was stirred overnight at room temperature. After completion of the reaction (monitored by GC-MS), the mixture was filtrated through one celite pad. The solvent was then removed under vacuum, and the mixture was then filtrated with pentane on one silica pad and evaporated to afford a mixture of **3 + menthane** (with slight vinylic unreduced product impurity) as a colourless liquid (14.75 g, 105 mmol in total, 80 % yield).

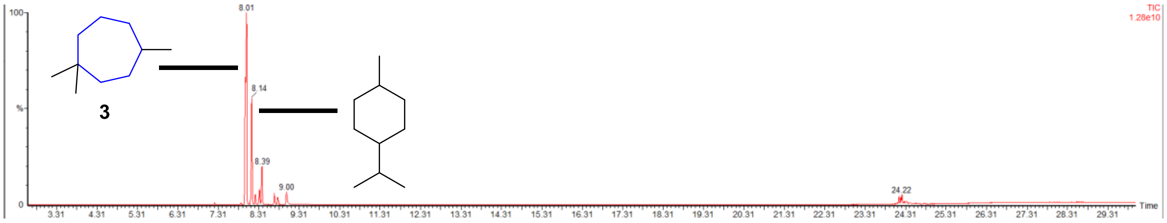

*Figure S38 : GC-MS chromatogram of **3** + menthane.*

Spectrum

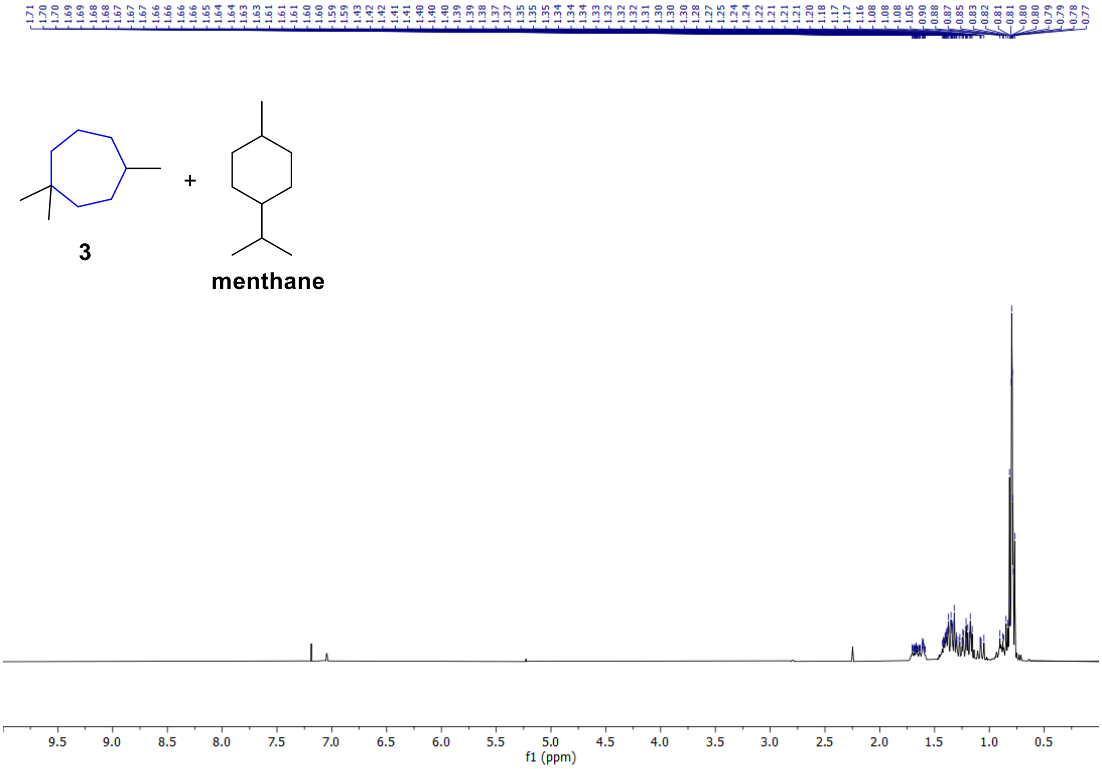

Figure S39 : ^{1}H spectrum of **3** + menthane mixture ($CDCl_3$).

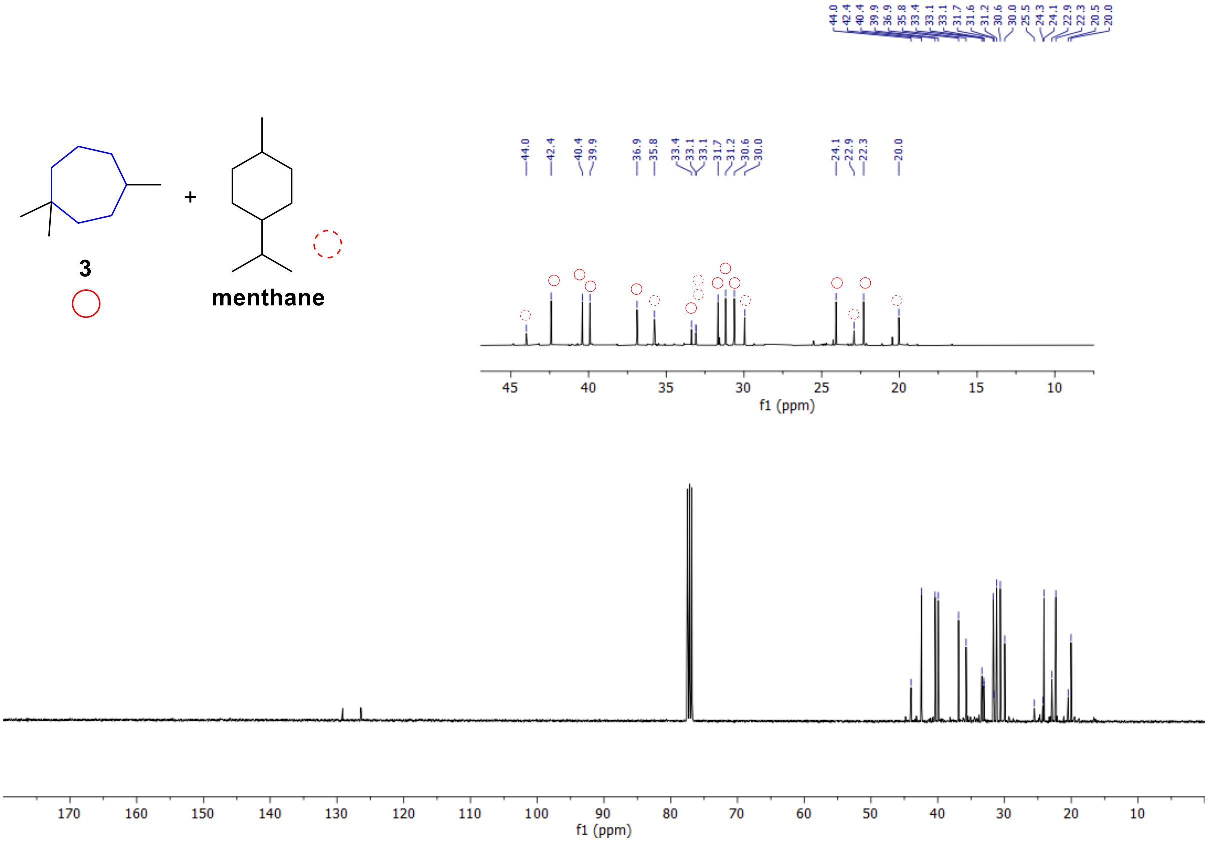

Figure S40 : ^{13}C spectrum of **3** + menthane ($CDCl_3$).

Electrocyclization of trienes

Catalytic electrocyclization

Successful trials

7

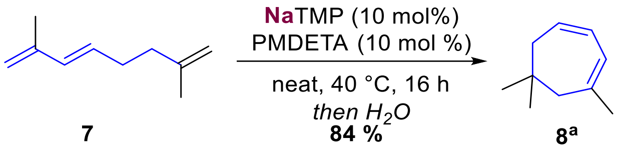

In the glovebox, in a J. Young NMR tube equipped with a flame-sealed Wilmad capillary filled with C_6D_6 was added **7** (1.5 mmol, 204 mg, 1 equiv) and PMDETA (0.15 mmol, 0.0313 mL, 10 mol%). The tube was removed from the glovebox, and a blank ^{1}H NMR spectrum was acquired. The tube was then returned to the glovebox, and NaTMP (24.49 mg, 0.15 mmol, 0.1 equiv) was added in a single portion. The sample was subsequently removed from the glovebox, maintained at 40°C, and the reaction progress was monitored by ^{1}H NMR spectroscopy. After completion of the reaction, the NMR tube was opened and quenched with two drops of deionized water. The tube was then extracted with pentane (3 ×), dried over $MgSO_4$, and filtered through a short silica pad. The solvent was slowly removed under reduced pressure (300 mbar, 1 h, 45 °C) to afford **8**[a] as a colourless oil (171 mg, 84 % yield.)

^{1}H NMR (400 MHz, $CDCl_3$) δ 5.80 – 5.71 (m, 1H), 5.71 – 5.62 (m, 2H), 2.03 (d, *J* = 2.0 Hz, 4H), 1.84 (d, *J* = 1.4 Hz, 3H), 0.97 (s, 6H). **^{13}C NMR** (101 MHz, $CDCl_3$) δ 140.6, 129.7, 126.5, 122.0, 48.8, 43.9, 43.9, 36.8, 29.3, 27.5, 27.5. **HRMS (EI)** m/z: $[M^•]^+$ Calcd for $C_{10}H_{16}$ 136.12465. Found 136.12478.

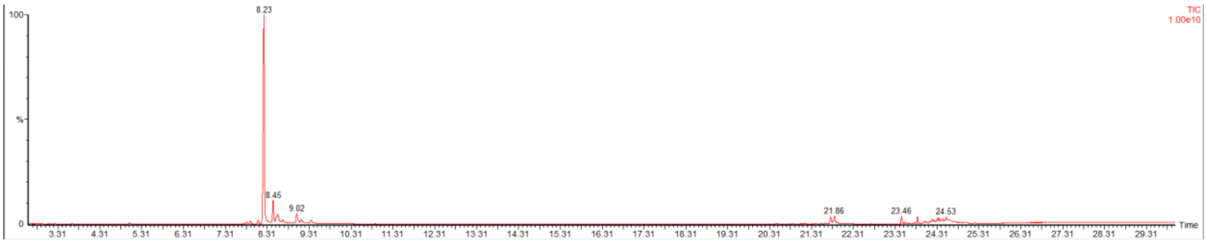

*Figure S41 : GC-MS chromatogram of **8**[a].*

9

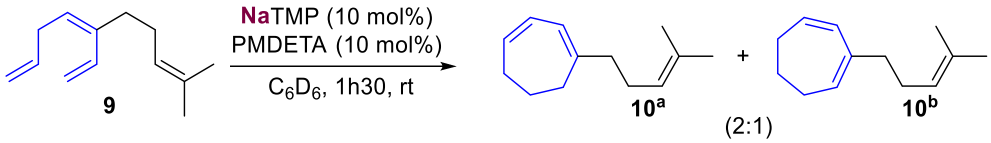

In the glovebox, in an J. Young NMR tube equipped was added **9** (0.5 mmol, 88.2 mg, 1 equiv), PMDETA (0.05 mmol, 0.0313 mL, 10 mol%) and C_6D_6 (0.5 mL). The tube was removed from the glovebox, and a blank ^{1}H NMR spectrum was acquired. The tube was then returned to the glovebox, and NaTMP (8.16 mg, 0.05 mmol, 10 mol%) was added in a single portion. The sample was subsequently removed from the glovebox, maintained

at room temperature, and the reaction progress was monitored by ^{1}H NMR spectroscopy. After completion of the reaction, the NMR tube was opened and quenched with two drops of deionized water. The tube was then extracted with pentane (3 ×), dried over $MgSO_4$, and filtered through a short silica pad. The solvent was slowly removed under reduced pressure to afford a mixture **10^a** and **10^b** in a 2:1 ratio as a colourless oil (86.2 mg, 98 % yield).

10^a: **^{1}H NMR** (400 MHz, $CDCl_3$) δ 5.73 – 5.70 (m, 2H), 5.56 (m, 1H), 5.11 (m, 1H), 2.35 – 2.26 (m, 4H), 2.15 – 1.97 (m, 4H), 1.89 – 1.78 (m, 2H), 1.68 (s, 3H), 1.61 (s, 3H). **^{13}C NMR** (101 MHz, $CDCl_3$) δ 146.5, 132.0, 131.7, 125.1, 124.2, 120.6, 41.0, 34.9, 32.2, 27.2, 25.9, 25.8, 17.8. **HRMS (EI)** m/z: $[M^•]^+$ Calcd for $C_{13}H_{20}$ 176.15595. Found 176.15620.

10^b : **^{1}H NMR** (400 MHz, $CDCl_3$) δ 5.83 (dt, J = 10.8, 5.1 Hz, 1H), 5.68 (m, 1H), 5.67 – 5.60 (m, 1H), 5.11 (m, 1H), 2.36 – 2.25 (m, 2H), 2.22 (q, J = 5.8 Hz, 2H), 2.17 – 1.96 (m, 4H), 1.88 – 1.79 (m, 1H), 1.69 (s, 3H), 1.60 (s, 3H). **^{13}C NMR** (101 MHz, $CDCl_3$) δ 142.5, 136.9, 131.6, 129.1, 128.8, 124.3, 39.8, 31.7, 30.0, 28.4, 27.8, 25.9, 25.8. **HRMS (EI)** m/z: $[M^•]^+$ Calcd for $C_{13}H_{20}$ 176.15595. Found 176.15614.

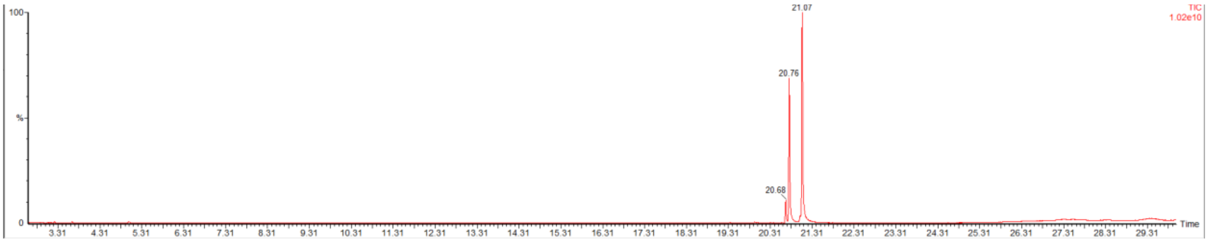

*Figure S42 : GC-MS chromatogram of **10^{a-b}** mixture.*

Unsuccessful trials

All subsequent experiments were carried out under the same conditions as those described for the catalytic cyclization of **9**, using either Na- or Li-based catalysis, and systematically afforded identical results, differing only in reaction time. The spectra presented herein were obtained from *in situ* analyses performed with NaTMP.

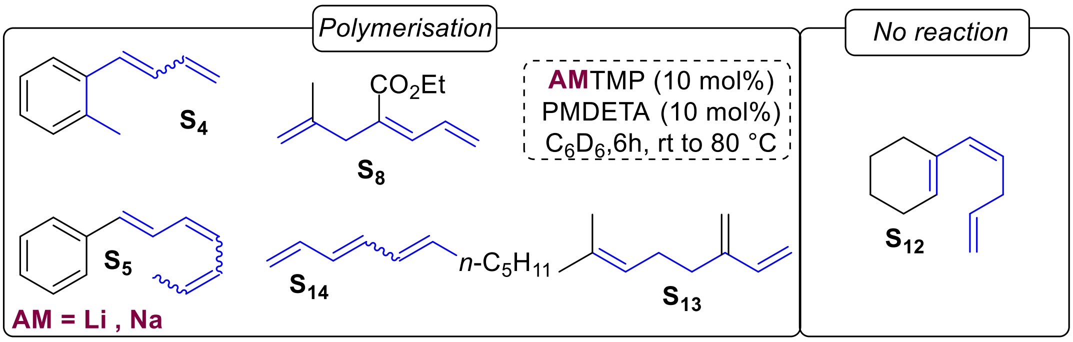

S_4

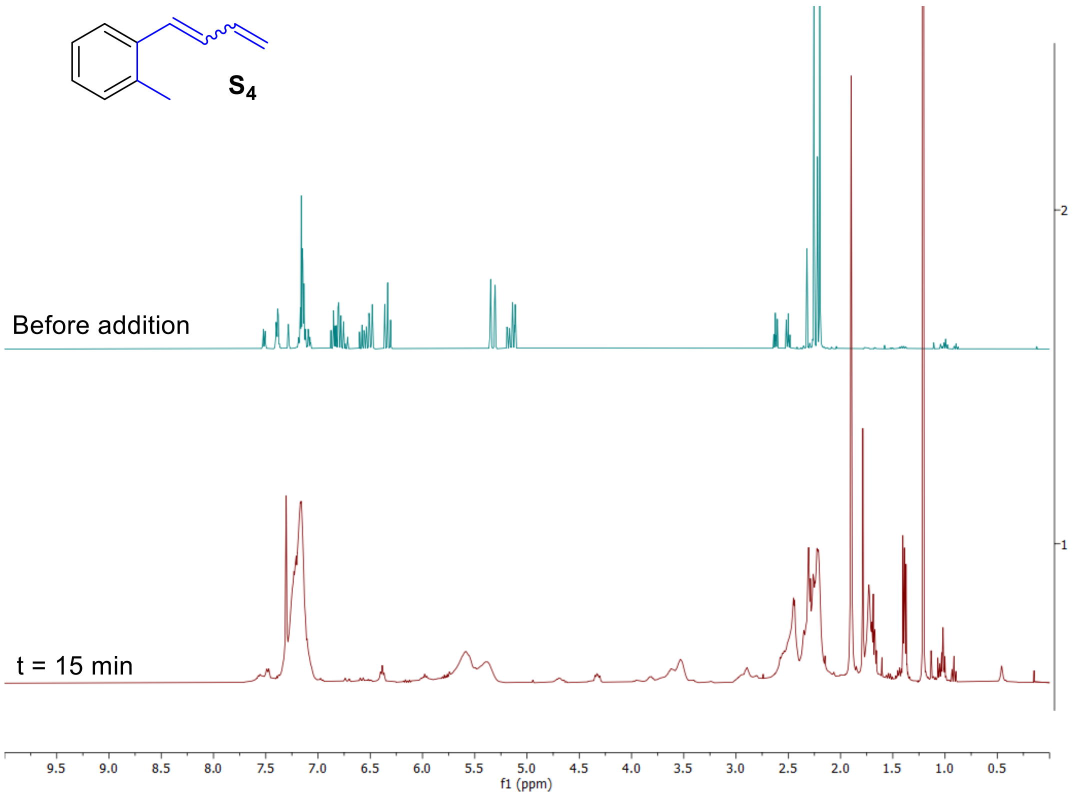

*Figure S43 : ^{1}H NMR spectrum of in-situ reaction with **S_4** (C_6D_6).*

S_5

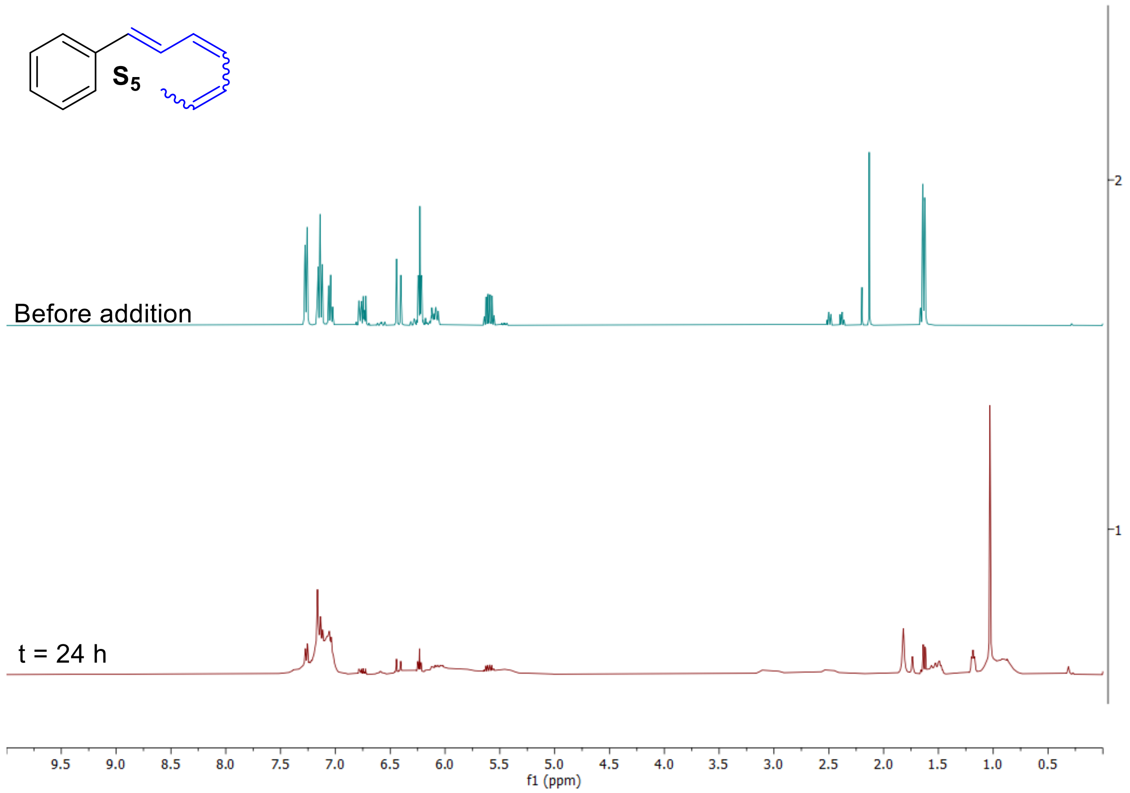

*Figure S44 : ^{1}H NMR spectrum of in-situ reaction with **S_5** (C_6D_6).*

S_8

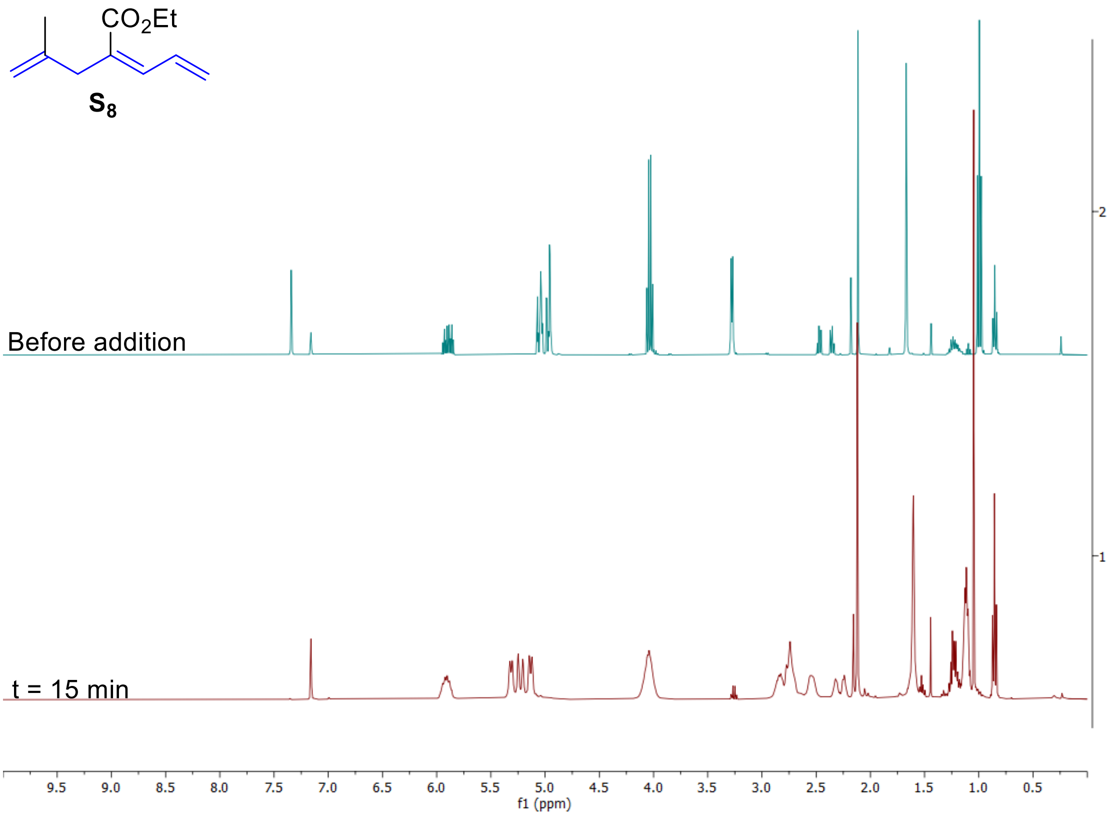

*Figure S45 : ¹H NMR spectrum of in-situ reaction with **S_8** (C_6D_6).*

S_{12}

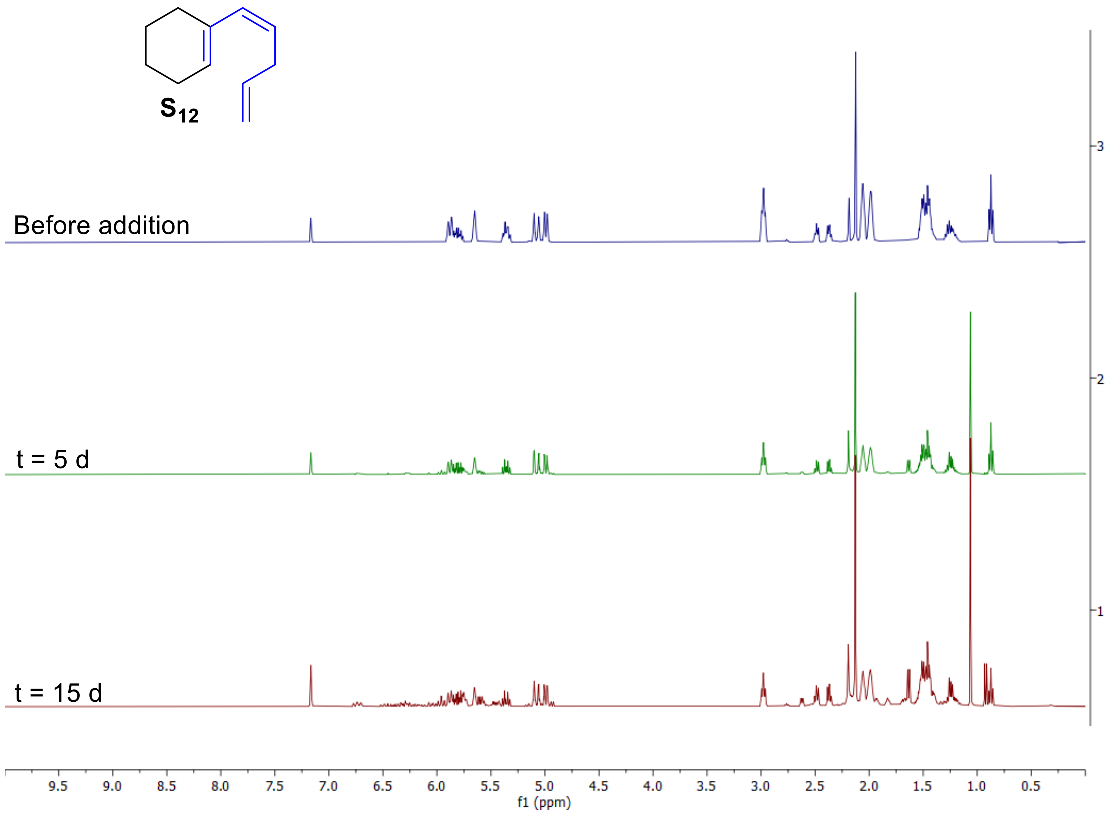

*Figure S46 : ¹H NMR spectrum of in-situ reaction with **S_{12}** (C_6D_6).*

S_{13}

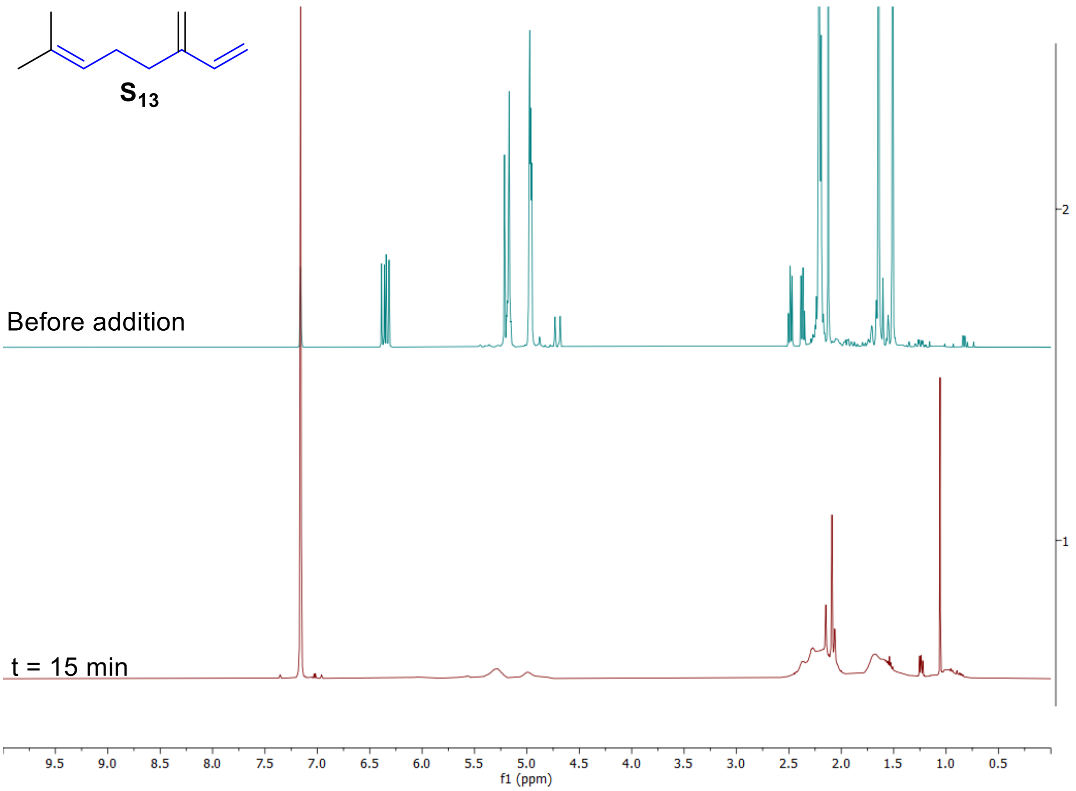

Figure S47 : ^{1}H NMR spectrum of in-situ reaction with ***S₁₃*** *(C_6D_6).*

S_{14}

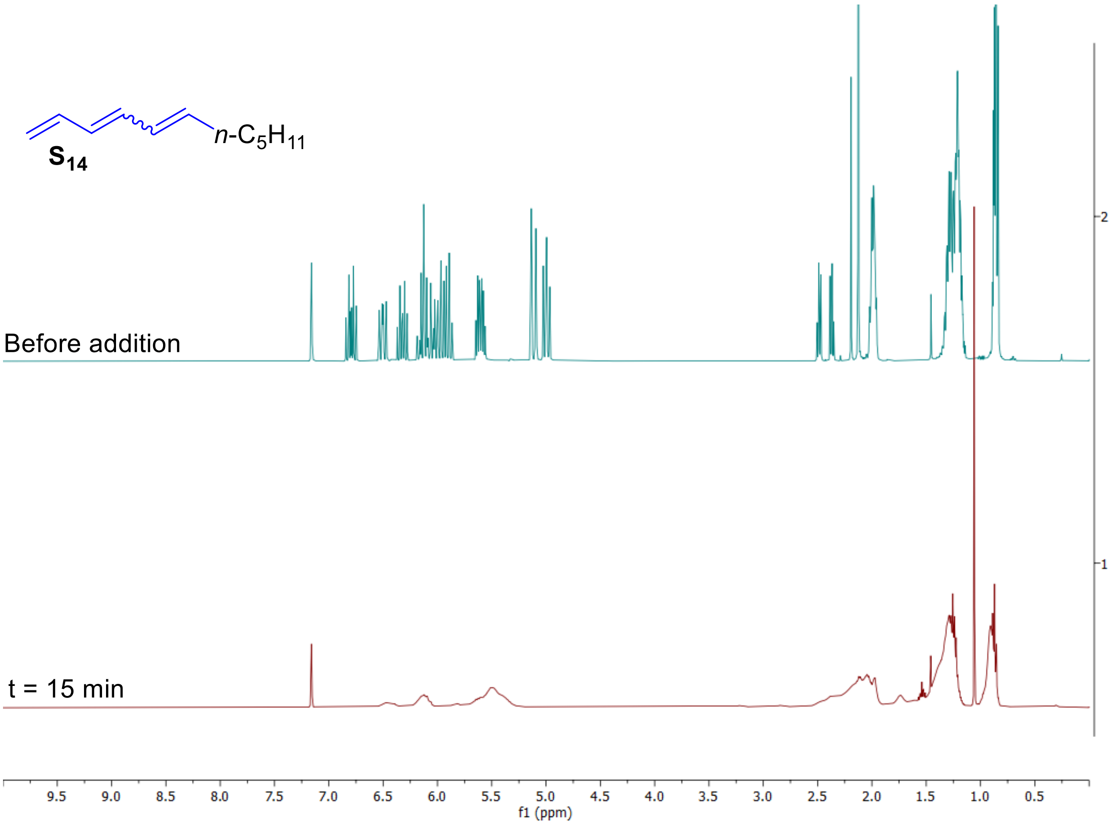

Figure S48 : ^{1}H NMR spectrum of in-situ reaction with ***S_{14}*** *(C_6D_6).*

Synthesis of starting materials

Compounds **S13** (CAS : 123-35-3) and **S14** (16356-11-9) were bought from commercial supplier Sigma-Aldrich.

7

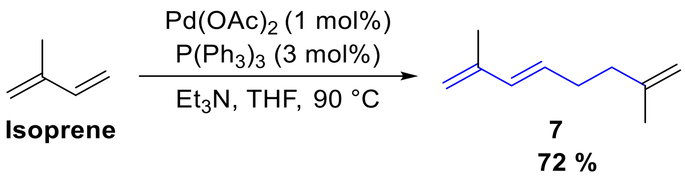

According to a reported procedure,[14] in the glovebox, to a 100 mL ampoule equipped with a magnetic stirrer was added $Pd(OAc)_2$ (112 mg, 0.5 mmol, 1 mol%), Ph_3P (393 mg, 1.5 mmol, 3 mol%) and isoprene (10 mL, 100 mmol, 2 equiv) in THF (50 mL). Then, Et3N (0.690 mL, 5 mmol, 10 mol%) was added. The ampoule was closed, removed from the glovebox and stirred at 90 °C in an oil bath for 48 hours. The mixture was then washed with 1M HCl and extracted 3x with pentane. The combined organic layer were concentrated under reduced pressure and filtrated on a silica pad to afford **7** (4,9 g, 36 mmol, 72% yield) as a colourless oil.

^{1}H NMR (400 MHz, $CDCl_3$) δ 6.17 (dt, *J* = 15.5, 1.6 Hz, 1H), 5.67 (dt, *J* = 15.7, 6.8 Hz, 1H), 4.91 – 4.85 (m, 2H), 4.88 (s, 2H), 2.33 – 2.19 (m, 2H), 1.83 (d, *J* = 1.1 Hz, 3H), 1.74 (d, *J* = 1.2 Hz, 3H). **^{13}C NMR** (101 MHz, $CDCl_3$) δ 145.5, 142.3, 133.1, 130.4, 114.5, 110.3, 37.7, 31.1, 22.5, 18.8.

Data agrees with those previously reported in the literature.[14]

9

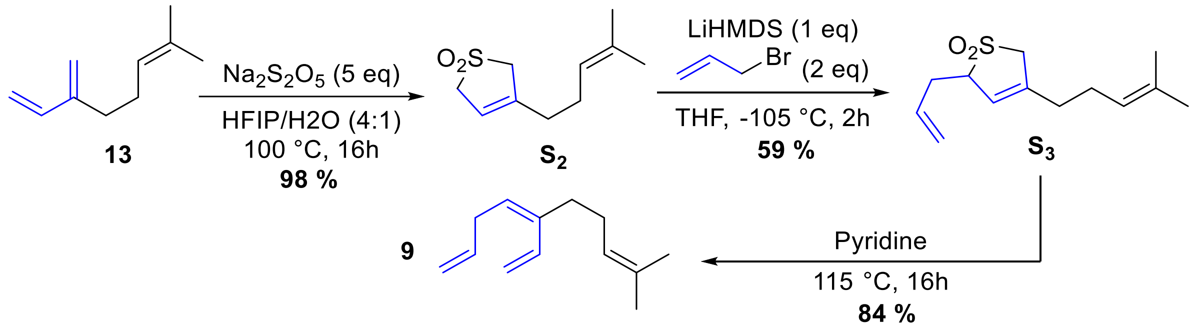

STEP 1 : According to a reported procedure,[7] to a 100 mL pressure tube equipped with a stirrer was added 40 mL of HFIP and 10 mL of deionized water. The pressure tube was closed with a septum, and the mixture degassed for 5 minutes by bubbling Argon. After degassing, **13** (5.15 mL, 30 mmol, 1 eq) and $Na_2S_2O_5$ (19.01g, 100 mmol, 5 eq) were added. The pressure tube was then closed and the mixture stirred for 16 h at 100 °C. Then, the reaction mixture was cooled down to room temperature, filtrated through a cotton pad to remove excess $Na_2S_2O_5$ and the solvent removed by evaporation. The biphasic mixture was subsequently extracted with EtOAc (3 × 30 mL), dried over $MgSO_4$ and concentrated under reduced pressure to afford the **S_2** product as a pale-yellow oil (5.90g, 29.5 mmol, 98 % yield).

[14] D. Kellner, M. Weger, A. Gini, O. G. Mancheño. *Beilstein J. Org. Chem.* **2017**, *13*, 1807–1815.

^{1}**H NMR** (400 MHz, CDCl3) δ 5.65 – 5.58 (m, 1H), 4.99 (ddd, J = 8.6, 4.3, 2.6 Hz, 1H), 3.72 (s, 2H), 3.61 (s, 2H), 2.19 – 2.04 (m, 4H), 1.63 (s, 3H), 1.54 (s, 3H). ^{13}**C NMR** (101 MHz, CDCl3) δ 138.48, 133.23, 122.41, 117.08, 57.86, 57.05, 33.06, 25.66, 25.45, 17.77.
Data agrees with those previously reported in the literature.[7]

STEP 2 : According to a literature procedure[15], to a 100 mL Schlenk flask equipped with a magnetic stirrer and placed under an argon atmosphere was added $\mathbf{S_2}$ (1.0 g, 5 mmol, 1 equiv) and freshly distilled allyl bromide (0.86 mL, 10 mmol, 2 equiv) in THF (10 mL). The reaction mixture was then cooled to −105 °C using a cyclohexene bath and allowed to equilibrate at this temperature. Subsequently, a solution of LiHMDS in THF (837 mg, 5 mmol, 1 equiv in 3 mL THF) was added dropwise over 10 minutes. After the addition was complete, the reaction mixture was allowed to warm gradually to 0 °C and was then quenched with a saturated NH_4Cl solution. The solvent was removed under reduced pressure and the resulting residue was extracted with EtOAc (3 × 15 mL) and washed with water (2 × 10 mL). Purification by column chromatography on silica gel using EtOAc/heptane (20:80, Rf = 0.27) afforded $\mathbf{S_3}$ as a pale-yellow oil (711 mg, 2.96 mmol, 59% yield).
^{1}**H NMR** (400 MHz, $CDCl_3$) δ 5.87 (ddt, *J* = 17.1, 10.3, 6.9 Hz, 1H), 5.71 – 5.66 (m, 1H), 5.22 (d, *J* = 17.1 Hz, 1H), 5.16 (d, *J* = 10.2 Hz, 1H), 5.09 – 5.04 (m, 1H), 3.77 – 3.57 (m, 3H), 2.70 – 2.53 (m, 2H), 2.26 – 2.08 (m, 4H), 1.69 (s, 3H), 1.61 (d, *J* = 1.3 Hz, 3H). ^{13}**C NMR** (101 MHz, $CDCl_3$) δ 142.4, 133.3, 132.7, 122.7, 119.0, 116.5, 66.1, 55.8, 32.2, 31.8, 25.8, 25.3, 17.9. **HRMS (APCI)** m/z: $[M+H]^+$ Calcd for $C_{13}H_{21}O_2S$ 241.12568. Found 241.12551.

STEP 3 : In a two-neck round-bottom flask equipped with a stirrer and a condenser, $\mathbf{S_3}$ (1.205 g, 5.0 mmol, 1 equiv) was dissolved in 10 mL of distilled pyridine. The mixture was then refluxed at 115 °C for 16 hours. After cooling to room temperature, 10 mL of 1 M HCl was added, and the mixture was extracted with pentane (3 × 20 mL). The combined organic layers were filtered through a silica pad and concentrated under reduced pressure to afford **9** as a translucent oil (741 mg, 4.20 mmol, 84% yield)
^{1}**H NMR** (400 MHz, CDCl3) δ 6.29 (dd, J = 17.5, 10.8 Hz, 1H), 5.82 (ddt, J = 17.1, 10.1, 6.3 Hz, 1H), 5.48 (t, J = 7.5 Hz, 1H), 5.22 – 5.11 (m, 2H), 5.10 – 4.90 (m, 3H), 2.89 (ddt, J = 7.8, 6.2, 1.6 Hz, 2H), 2.26 (d, J = 10.0 Hz, 1H), 2.24 (d, J = 8.5 Hz, 1H) 2.08 (q, J = 7.7 Hz, 2H), 1.69 (q, J = 1.3 Hz, 3H), 1.60 (d, J = 1.3 Hz, 3H). ^{13}**C NMR** (101 MHz, CDCl3) δ 140.2, 139.3, 136.8, 132.0, 130.2, 124.3, 115.2, 111.0, 32.7, 27.6, 26.5, 25.9, 17.8. **HRMS (APCI)** m/z: $[M+H]^+$ Calcd for $C_{13}H_{21}$ 177.16378. Found 177.16419.

[15] S. R. Desai, V. K. Gore, T. Mayelvaganan, R. Padmakumar, S. V. Bhat. *Tetrahedron* **1992**, *48* (3), 481–490.

S_4

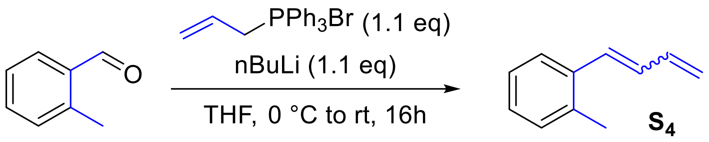

To a 100 mL Schlenk flask equipped with a magnetic stirrer and placed under an argon atmosphere was added allylbromotriphenyl-l5-phosphane (4.2 g, 11 mmol, 1.1 equiv) and dry THF (50 mL). The solution was cooled down to 0°C and *n*BuLi (6.9 mL of a 1.6 M solution in hexanes) was slowly added. The mixture was stirred for 30 mins then 2-methylbenzaldehyde (1.2 g, 10 mmol, 1 equiv) was added in one portion and the mixture stirred overnight. After complete consumption, the mixture was quenched with sat. NH_4Cl and extracted with pentane (3x15 mL). The combined organic layer was dried with Na_2SO_4 and concentrated under reduced pressure. The mixture was then filtered twice on long silica pad to afford **S_4** as a colourless oil as a 1:2 (Z/E) mixture of diastereoisomers (711 mg, 4.9 mmol, 49 % yield).

^{1}H NMR (400 MHz, $CDCl_3$) δ 7.63 – 7.54 (m, 1H, *E* isomer), 7.35 – 7.29 (m, 1H, *Z* isomer), 7.29 – 7.19 (m, 3H, *Z*+*E* isomer), 6.90 (d, *J* = 15.4 Hz, 1H, *E* isomer), 6.83 – 6.53 (m, 2H for *E* isomer, 3H for *Z* isomer), 6.39 (tt, *J* = 11.2, 0.8 Hz, 1H for *Z* isomer), 5.47 – 5.37 (m, 1H, *Z*+*E* isomers), 5.29 – 5.20 (m, 1H, *Z*+*E* isomers), 2.44 (s, 3H, *E* isomer), 2.35 (s, 3H, *Z* isomer). **^{13}C NMR** (101 MHz, $CDCl_3$) δ 137.7 (*E* isomer), 136.5 (*Z* isomer), 136.2 (*Z* isomer), 135.8 (*Z* isomer), 133.6 (*E* isomer), 131.0 (*Z* isomer), 130.9 (*E* isomer), 130.6 *(E* isomer), 130.5 *(E* isomer), 130.0 (*Z* isomer), 129.9 (*Z* isomer) 129.7 (*Z* isomer), 127.7 (*E* isomer), 127.4 (*Z* isomer), 126.2 (*E* isomer), 125.5 (*Z* isomer) , 125.3 (*E* isomer), 119.1 (*Z* isomer), 117.6 (*E* isomer), 20.0 (*Z* isomer), 19.9 (*E* isomer).
Data agrees with those previously reported in the literature.[16]

S_5

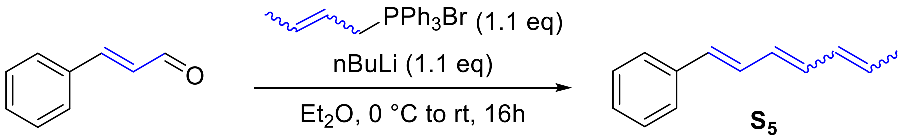

To a 100 mL Schlenk flask equipped with a magnetic stirrer and placed under an argon atmosphere was added bromo(but-2-en-1-yl)triphenyl-l5-phosphane (5.7 g, 14 mmol, 1. equiv) and dry Et_2O (50 mL). The solution was cooled down to 0°C and *n*BuLi (5.7 mL of a 2.5 M solution in hexanes) was slowly added. The mixture was stirred for 30 mins then 2-cinnamaldehyde (1.9 mL, 15 mmol, 1 equiv) was added in one portion and the mixture stirred overnight. After complete consumption, the mixture was quenched with sat. NH_4Cl and extracted with Et_2O (3x15 mL). The combined organic layer were dried with Na_2SO_4 and concentrated under reduced pressure. The mixture was then filtered twice on long silica pad to afford **S_5** as a waxy solid as a mixture of diastereoisomers that isomerize to only one diastereoisomer (major) over time (1.6 g, 9.4 mmol, 66 % yield).

---

[16] F. A. Khan, B. M. Budanur. *Tetrahedron* **2015**, *71* (40), 7600–7607.

Assignation of the peaks has been performed using HSQC multinuclear NMR. Only the two major stereoisomers are described.

^{1}H NMR (400 MHz, $CDCl_3$) δ 7.38 – 7.31 (*min,* d, *J* = 7.4 Hz, 2H), 7.31 – 7.26 (*maj,* d, *J* = 7.4 Hz, 2H), 7.28 – 7.07 (*maj+min*, m, 3H), 6.71 (*maj*, dd, *J* = 15.6, 9.5 Hz, 1H), 6.62 – 6.49 (*min*, m, 2H), 6.43 (*maj+min*, m, 1H), 6.29 – 6.13 (*maj*, m, 2H), 6.06 (*maj*, m, 1H), 6.00 – 5.91 (*min,* m, 2H), 5.70 (*maj + min*, ddt, *J* = 21.9, 13.9, 6.8 Hz, 1H), 1.77 (*min*, dd, *J* = 6.9, 1.6 Hz, 3H), 1.72 (*maj,* dd, *J* = 6.8, 1.6 Hz, 3H). **^{13}C NMR** (101 MHz, $CDCl_3$) δ 137.6 (*maj),* 137.5 *(min)*, 133.7 *(maj)*, 132.4 *(min)*, 131.9 *(maj)*, 131.5 *(maj)*, 131.3 *(maj)*, 130.6 *(maj)*, 130.4 *(maj)*, 130.3 *(min)*, 129.4 *(maj)*, 128.6 *(min)*, 128.6 *(maj)*, 127.5 *(min)*, 127.3 *(min)*, 127.3 *(maj)*, 126.4 *(min)*, 126.2 *(maj)*, 124.4 *(min*), 18.6 *(min)*, 18.4 *(maj)*.

S_8

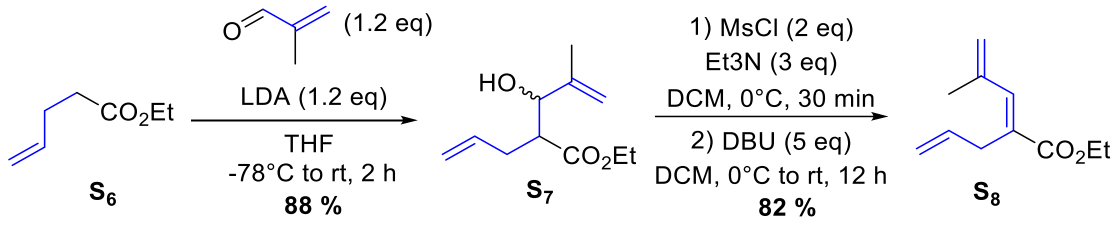

STEP 1 : According to a litterature procedure[17], In a 100 mL Schlenk flask equipped with a magnetic stir bar and maintained under an argon atmosphere, *i*Pr_2NH (1.6 mL, 12 mmol, 1.2 equiv) was dissolved in THF (6.7 mL). The solution was cooled to −78 °C, and n-BuLi (7.5 mL, 1.6 M in hexanes) was added dropwise. After complete addition, the reaction mixture was allowed to warm to 0 °C and stirred for 30 min. The mixture was then cooled again to −78 °C, and ethyl pent-4-enoate **S_6** (1.72 mL, 12 mmol, 1.2 equiv) was added. Stirring was continued at −78 °C for 30 min, after which methacrolein (0.828 mL, 10 mmol, 1.0 equiv) was added slowly. The reaction mixture was then allowed to warm to room temperature and stirred for 2 h, until complete consumption of the starting material. The reaction was quenched with saturated aqueous NH_4Cl, and the mixture was extracted with Et_2O (3 × 15 mL). The combined organic layers were dried over Na_2SO_4, filtered, and concentrated under reduced pressure. Purification by column chromatography on silica gel using EtOAc/Heptane (1:10, Rf = 0.29) afforded the two diastereoisomers **S_7** as an pale-yellow oil (1.75 g, 8.8 mmol, 88% yield).

***Syn*-S_5** : **^{1}H NMR** (400 MHz, $CDCl_3$) δ 5.80 (ddt, J = 17.1, 10.1, 7.0 Hz, 1H), 5.12 – 4.90 (m, 4H), 4.35 – 4.28 (m, 1H), 4.14 (q, J = 7.1 Hz, 2H), 2.67 (td, J = 7.2, 5.7 Hz, 1H), 2.49 – 2.37 (m, 3H), 1.74 (s, 3H), 1.25 (t, J = 7.1 Hz, 3H). **^{13}C NMR** (101 MHz, $CDCl_3$) δ 174.4, 144.2, 135.9, 116.8, 113.1, 75.6, 60.8, 48.9, 31.4, 18.4, 14.3. **HRMS (ESI)** m/z: $[M+Na]^+$ Calcd for $C_{11}H_{18}O_3Na$ 221.11454. Found 221.11443.

***Anti*-S_5 :** **^{1}H NMR** (400 MHz, $CDCl_3$) δ 5.74 (ddt, J = 17.1, 10.1, 7.0 Hz, 1H), 5.15 – 4.87 (m, 4H), 4.24 – 4.09 (m, 3H), 2.74 – 2.62 (m, 2H), 2.44 – 2.23 (m, 2H), 1.74 (s, 3H), 1.25 (t, J = 7.1 Hz, 3H). **^{13}C NMR** (101 MHz, $CDCl_3$) δ 174.7, 144.8, 134.7, 117.4, 113.5, 76.2, 60.8, 48.6, 34.0, 17.8, 14.4. **HRMS (ESI)** m/z: $[M+Na]^+$ Calcd for $C_{11}H_{18}O_3Na$ 221.11454. Found 221.11470.

[17] R. Kato, H. Saito, S. Uda, D. Domon, K. Ikeuchi, R. Suzuki, K. Tanino. *Org. Lett.* **2021**, *23* (22), 8878–8882.

STEP 2 : According to a literature procedure[17], in a 100 mL round-bottom flask equipped with a stirrer was added DCM (40 mL) and **S_7** (1.68 g, 8.5 mmol, 1 equiv). The mixture was cooled to 0°C and Et_3N (3.55 mL, 25.5 mmol, 3 equiv) & MsCl (1.32 mL, 17.0 mmol, 2 equiv) were added successively. After 30 mins of stirring at 0°C, DBU (6.3 mL, 42.5 mmol, 5 equiv) was added. The reaction was allowed to warmup at room temperature and stirred overnight. After complete conversion of the starting material, the reaction was quenched with sat. $NaHCO_3$ and extracted with Et_2O (3x15 mL). The resulting residue was dried over Na_2SO_4 and concentrated under reduced pressure (volatile product, 350 mbar for 30 min, 45°C). Purification by column chromatography on silica gel using Et_2O/Pentane (1:12, Rf = 0.7) afforded **S_8** as a colourless oil (1.25 g, 6.9 mmol, 82% yield). **^{1}H NMR** (400 MHz, $CDCl_3$) δ 7.20 (s, 1H), 5.88 (ddt, J = 16.5, 10.7, 5.8 Hz, 1H), 5.15 (ddt, J = 16.4, 2.1, 1.3 Hz, 2H), 5.03 (t, J = 1.8 Hz, 1H), 4.99 (dq, J = 8.2, 1.7 Hz, 1H), 4.21 (q, J = 7.1 Hz, 2H), 3.24 (dtd, J = 5.8, 1.7, 0.6 Hz, 2H), 1.93 (dd, J = 1.6, 0.8 Hz, 3H), 1.30 (t, J = 7.1 Hz, 3H). **^{13}C NMR** (101 MHz, $CDCl_3$) δ 168.3, 141.9, 140.7, 136.5, 129.7, 119.5, 115.4, 60.9, 31.7, 22.5, 14.2. **HRMS (EI)** m/z: $[M^{\bullet}]^+$ Calcd for $C_{11}H_{16}O_2$ 180.11448. Found 180.11456.

S_{12}

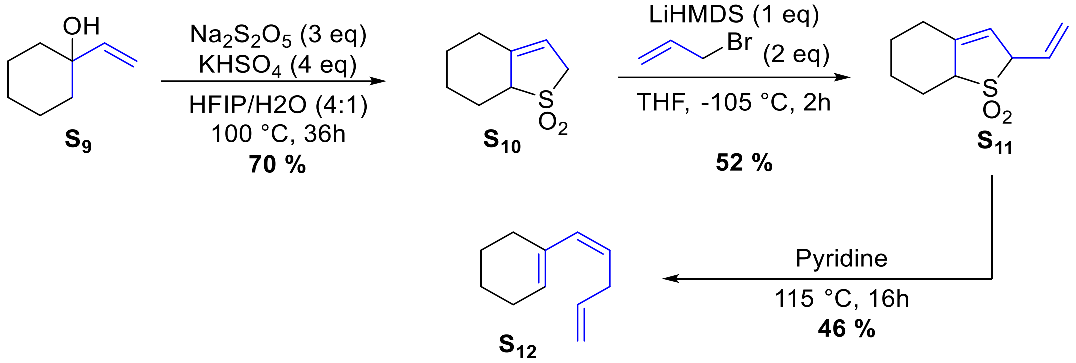

STEP 1 : According to a reported procedure,[7] to a 100 mL pressure tube equipped with a stirrer was added 30 mL of HFIP and 10 mL of deionized water. The pressure tube was closed with a septum and the mixture degassed 5 minutes by bubbling Argon. After degassing, **S_9** (2 mL, 15 mmol, 1 eq), $Na_2S_2O_5$ (8.6g, 45 mmol, 3 eq) and $KHSO_4$ (8.1g, 60 mmol, 4 eq) were added. The pressure tube was then closed and the mixture stirred for 15 min at rt and 36 h at 100 °C. Then, the reaction mixture was cooled down to room temperature, the mixture filtrated through a filter to remove excess $Na_2S_2O_5$ and the solvent removed by evaporation. The biphasic mixture was subsequently extracted with EtOAc (3 × 30 mL), dried over $MgSO_4$ and concentrated under reduced pressure to afford the **S_{10}** product as an pale-yellow oil (1.80g, 10.5 mmol, 70 % yield).
^{1}H NMR (400 MHz, CDCl3) δ 5.58 (p, J = 2.5 Hz, 1H), 3.77 (dddd, J = 16.2, 4.6, 2.3, 1.3 Hz, 1H), 3.64 (ddt, J = 16.2, 3.1, 2.1 Hz, 1H), 3.55 (ddp, J = 11.4, 5.7, 1.8 Hz, 1H), 2.48 (ddt, J = 14.3, 4.0, 1.9 Hz, 1H), 2.29 – 2.18 (m, 1H), 2.11 (dddt, J = 15.9, 11.7, 4.6, 2.4 Hz, 1H), 1.97 (dddt, J = 14.9, 5.3, 3.5, 1.8 Hz, 1H), 1.86 (dddt, J = 13.5, 5.3, 3.4, 1.6 Hz, 1H), 1.69 – 1.57 (m, 1H), 1.44 (qt, J = 13.2, 3.2 Hz, 1H), 1.27 (dd, J = 13.2, 3.2 Hz, 1H). **^{13}C NMR** (101 MHz, CDCl3) δ 140.6, 113.1, 64.2, 56.2, 31.6, 25.9, 25.6, 23.9.
Data agrees with those previously reported in the literature. [7]

STEP 2 : According to a literature procedure[15], to a 100 mL Schlenk flask equipped with a magnetic stirrer and placed under an argon atmosphere was added **S_{10}** (1.7 g, 10 mmol,

1 equiv) and freshly distilled allyl bromide (1.7 mL, 20 mmol, 2 equiv) in THF (20 mL). The reaction mixture was then cooled to −105 °C using a cyclohexene bath and allowed to equilibrate at this temperature. Subsequently, a solution of LiHMDS in THF (1.8 g, 11 mmol, 1.05 equiv in 6 mL THF) was added dropwise over 10 minutes. After the addition was complete, the reaction mixture was allowed to warm gradually to 0 °C and was then quenched with a saturated NH_4Cl solution. The solvent was removed under reduced pressure, and the resulting residue was extracted with EtOAc (3 × 15 mL) and washed with water (2 × 10 mL). Purification by column chromatography on silica gel using Et_2O/Pentane (25:75, Rf = 0.26) afforded $\mathbf{S_{11}}$ as a pale-yellow oil (1.1 g, 5.2 mmol, 52% yield).

^{1}H NMR (400 MHz, $CDCl_3$) δ 5.68 (dddd, J = 17.6, 10.2, 7.6, 6.3 Hz, 1H), 5.46 (q, J = 2.5 Hz, 1H), 5.08 – 4.95 (m, 2H), 3.52 – 3.42 (m, 1H), 3.34 (ddt, J = 10.9, 5.0, 1.5 Hz, 1H), 2.56 (dtt, J = 14.0, 6.2, 1.5 Hz, 1H), 2.31 (ddt, J = 14.1, 4.2, 1.9 Hz, 1H), 2.24 – 2.05 (m, 2H), 2.04 – 1.89 (m, 1H), 1.81 (dqd, J = 13.2, 3.3, 1.5 Hz, 1H), 1.71 (dqd, J = 13.4, 3.2, 1.2 Hz, 1H), 1.49 (tdd, J = 13.1, 12.1, 3.7 Hz, 1H), 1.40 – 1.19 (m, 1H), 1.22 – 0.99 (m, 1H). **^{13}C NMR** (101 MHz, $CDCl_3$) δ 139.3, 133.1, 118.4, 64.4, 64.0, 34.5, 31.4, 25.8, 25.6. **HRMS (APCI)** m/z: $[M+H]^+$ Calcd for $C_{11}H_{17}O_2S$ 213.09438. Found 213.09484.

STEP 3 : In a two-neck round-bottom flask equipped with a stirrer and a condenser, $\mathbf{S_{11}}$ (900 mg, 4.24 mmol, 1 equiv) was dissolved in 10 mL of distilled pyridine. The mixture was then refluxed at 115 °C for 16 hours. After cooling to room temperature, 10 mL of 1 M HCl was added, and the mixture was extracted with pentane (3 × 20 mL). The combined organic layers were filtered through a silica pad and concentrated under reduced pressure to afford $\mathbf{S_{12}}$ as a translucent oil (296 mg, 2.0 mmol, 46% yield)

^{1}H NMR (400 MHz, $CDCl_3$) δ 5.85 – 5.70 (m, 2H), 5.57 (tt, J = 3.7, 1.6 Hz, 1H), 5.24 (dt, J = 11.7, 7.6 Hz, 1H), 4.98 (dq, J = 17.2, 1.8 Hz, 1H), 4.92 (dq, J = 10.1, 1.6 Hz, 1H), 2.97 – 2.88 (m, 2H), 2.12 – 1.98 (m, 5H), 1.65 – 1.43 (m, 4H). **^{13}C NMR** (101 MHz, CDCl3) δ 137.7, 135.2, 132.9, 127.4, 126.1, 33.3, 29.1, 25.7, 23.0, 22.3. **HRMS (EI)** m/z: $[M^{\bullet}]^+$ Calcd for $C_{11}H_{16}$ 148.12465. Found 148.12472.

DFT calculations

Computational method

All calculations were performed using the ORCA 6.0 or 6.1 package.[18] Reaction mechanisms were elucidated through density functional theory (DFT) using dispersion-corrected range-separated functional ωB97X[19] with zero damping D3 dispersion correction [20] and Ahlrichs polarized valence triple-ζ basis set DEF2-TZVP[21] for all atoms.

Geometry optimizations were performed in the gas phase without imposing any symmetry constraints. The nature of all stationary points (minima and transition states) was confirmed by analytical frequency calculations: minima exhibited no imaginary frequencies, whereas transition states showed a single imaginary frequency. Each optimized transition state was further validated by relaxation along the reaction coordinate in both directions to ensure connectivity between the appropriate energy minima. DeltaG are given at 298.15 K and 1 atm pressure.

All DFT data underlying the present work, including inputs and outputs are openly accessible via ioChem-BD repository : https://doi.org/10.19061/iochem-bd-6-630

---

[18] (*a*) F. Neese. *WIREs Comput. Mol. Sci.* **2025**, *15* (2), e70019. (*b*) F. Neese. *J. Comput. Chem.* **2003**, *24* (14), 1740–1747. (c) F. Neese, F. Wennmohs, A. Hansen, U. Becker. *Chem. Phys.* **2009**, *356* (1–3), 98–109. (*d*) S. Grimme, J. Antony, S. Ehrlich, H. Krieg. *J. Chem. Phys.* **2010**, *132* (15), 154104. (*e*) D. Bykov, T. Petrenko, R. Izsák, S. Kossmann, U. Becker, E. Valeev, F. Neese. *Mol. Phys.* **2015**, *113* (13–14), 1961–1977. (*f*) B. Helmich-Paris, B. De Souza, F. Neese, R.J. Izsák. *Chem. Phys.* **2021**, *155* (10), 104109. (*g*) F. Neese. *J. Comput. Chem.* **2023**, *44* (3), 381–396.

[19] Y.-S. Lin, G.-D. Li, S.-P. Mao, J.-D. Chai. *J. Chem. Theory Comput.* **2013**, *9* (1), 263–272.

[20] S. Grimme, J. Antony, S.Ehrlich, H. Krieg. *J. Chem. Phys.* **2010**, *132* (15), 154104.

[21] F. Weigend, R. Ahlrichs. *Phys. Chem. Chem. Phys.* **2005**, *7* (18), 3297.

Stoichiometric deprotonation and cyclization

Energies are calculated using the energies of separated species **AMTMP** + **1** in Hartree and converted to kcal/mol.

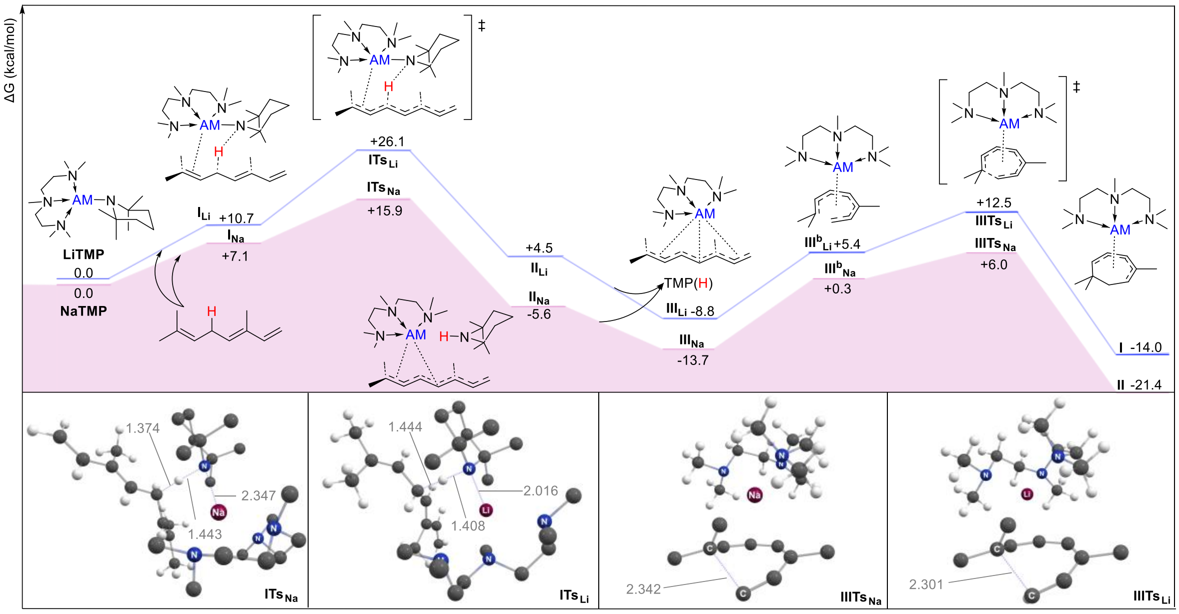

*Figure S49 : Lowest Gibbs energy profile of electrocyclization of β-Ocimene **1** calculated in gas phase at T=298.15K and P = 1atm where AM = Li (blue path) or Na (pink path) (kcal/mol). Structures of TS are displayed with relevant bond length in Å, with some H omitted for clarity. Images have been generated using Chemcraft software.*

First step involves the approach of **AMTMP** and **1** forming the adduct I_{AM}, allowing the superbase to deprotonate ocimene through **ITS**$_{AM}$ forming a transition state where the N–H and C–H bonds are in longer than classical double-bond, typical for deprotonation TS. Then, after decoordination and removal of newly formed TMP(H) from the system, the heptatrienyl anion **III**$_{AM}$ is formed with a strong delocalized character, as the former is completely linear.

Then this heptatrienyl anion can twist to form the 'band-like' structure, placing the terminal carbons in a perfect position (2.3 Å apart one from another) for the electrocyclization to occur and form the corresponding pentadienyl anion **I** and **II**.

Catalytic turnover

To understand the catalytic process, DFT calculations have been performed on the deprotonation of the resting state **I** by neo-allocimene **1'** (major in solution) and TMP(H). Regarding the structure of obtained cycles, three deprotonations' sites are possible (C_3, C_5 and C_7) yielding to the 3 known products. Then, catalytic protonation/deprotonation between **I** and **4**$^{a\text{-}c}$ allows the formation of the most stable, conjugated products **4**^{a} and **4**^{b} observed experimentally.

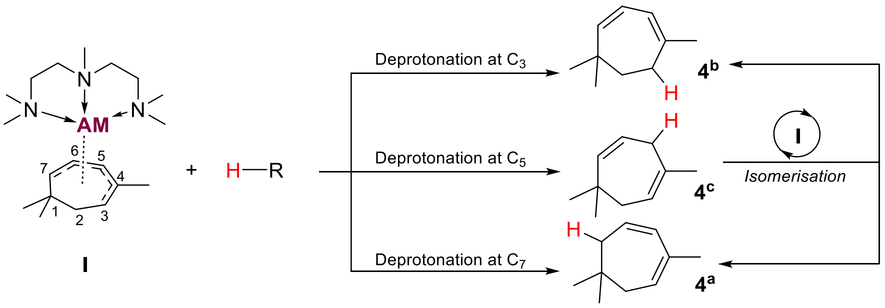

*Figure S50 : Possible deprotonations sites of **I**.*

Deprotonation of **I** at C_3

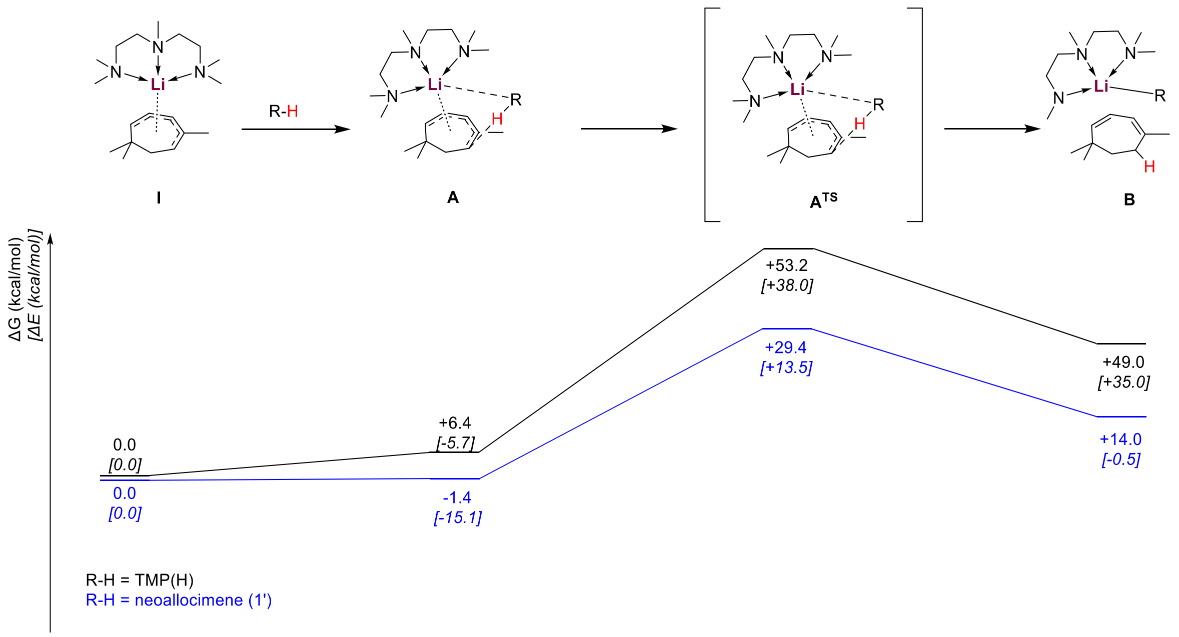

*Figure S51 : Lowest Gibbs energy profile of **I** deprotonation by TMP(H) or neoalloocimene in C_3 (in kcal.mol^{-1}). (ωB97X-D3 / DEF2-TZVP / Gas phase)*

Deprotonation of **I** at C_5

For the deprotonation at C_5, decoordination of one PMDETA arm was necessary to locate transitions states, due to high steric hindrance. TS of deprotonation with TMP(H) was not localized for this position.

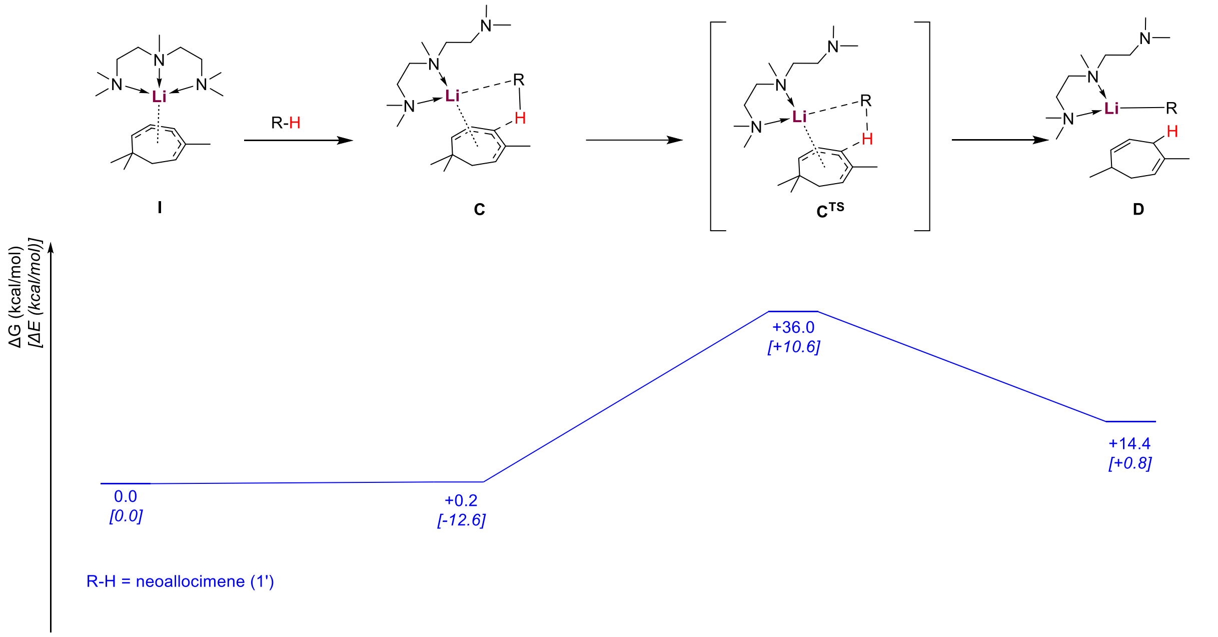

*Figure S52 : Lowest Gibbs energy profile of **I** deprotonation by TMP(H) or neoalloocimene in C_5 (in kcal.mol^{-1}). (ωB97X-D3 / DEF2-TZVP / Gas phase)*

Deprotonation of **I** at C_7

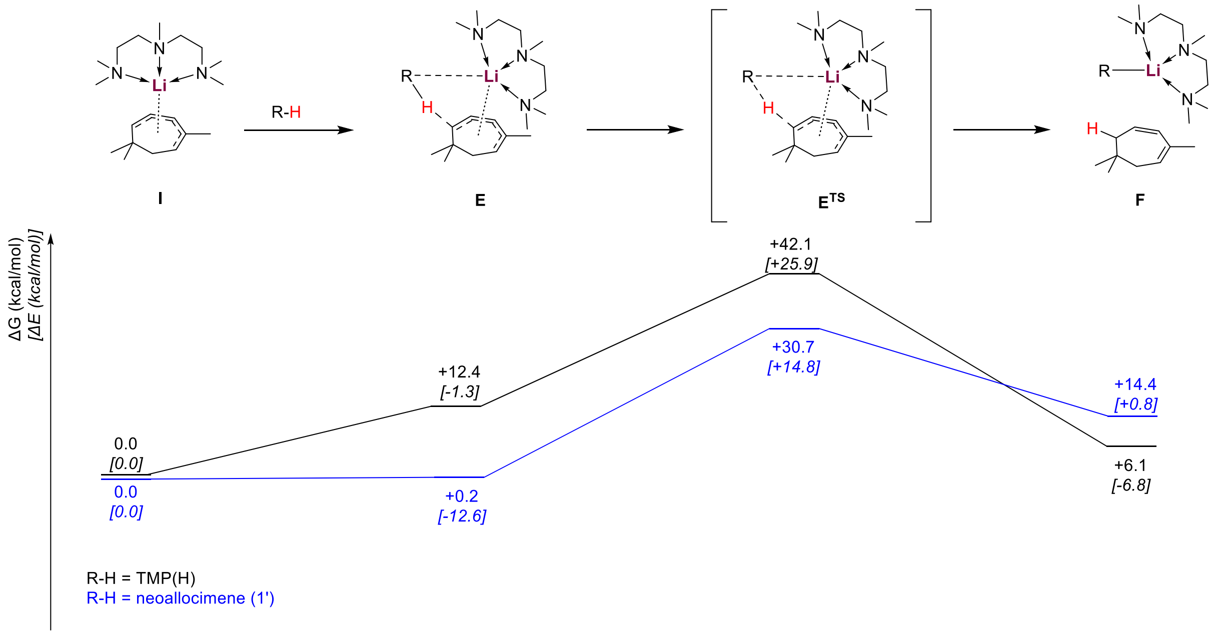

*Figure S53 : Lowest Gibbs energy profile of **I** deprotonation by TMP(H) or neoalloocimene in C_7 (in kcal.mol^{-1}). (ωB97X-D3 / DEF2-TZVP / Gas phase)*

Implicit solvation

To assess how implicit solvation influences computed energetics, we carried out optimization calculation on one of the lowest TS found in gas phase (**A**^{TS}, deprotonation at C_3) using two solvent models. First, we used a custom CPCM[22] built from the experimental refractive index of ocimene (n^{20}/D = 1.485) and the dielectric constant of limonene (ε = 2.36), since ε for ocimene could not be found in the literature. Second, we performed SMD[23] calculations in benzene, which we consider the closest standard solvent for our reaction conditions. The ΔG values reported below correspond to the free-energy difference between the separated reactants and one of the lowest-energy transition state found in gas phase (**A**^{TS}), after applying the standard-state correction (-1.9 kcal/mol for associative steps) from the gas phase (1 atm) to the condensed phase (1 M).

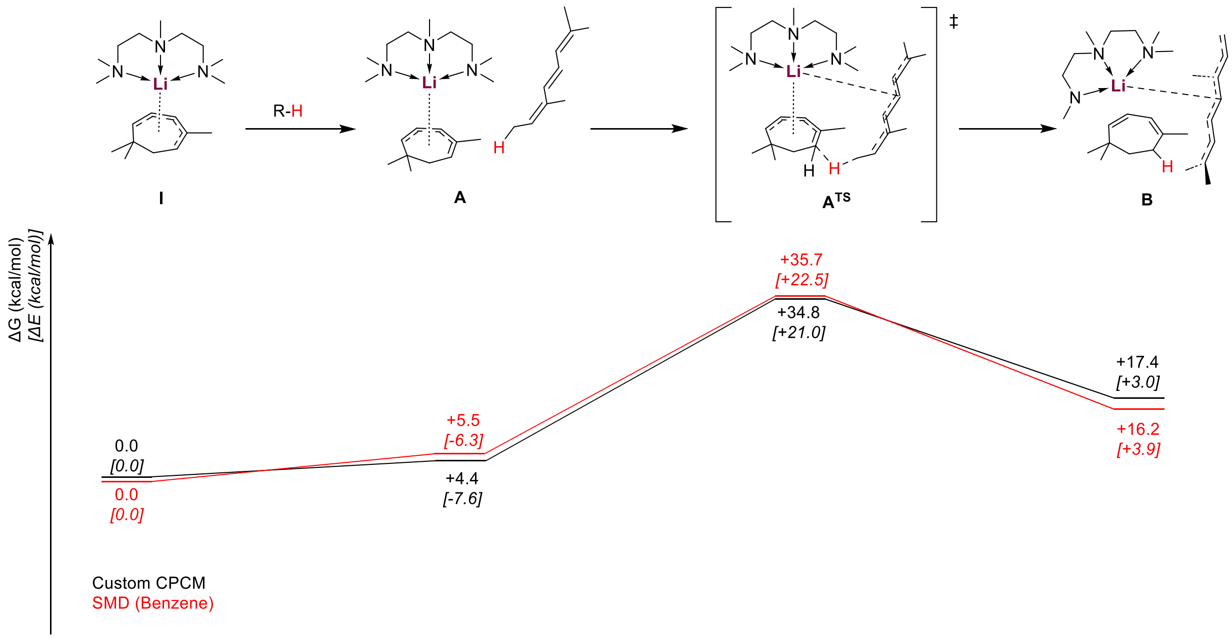

*Figure S54 : Lowest Gibbs energy profile of **I** deprotonation of neoalloocimene at C_3 with solvation (in kcal.mol^{-1}). (ωB97X-D3 / DEF2-TZVP / Custom CPCM (black) / SMD (benzene) (red)).*

[22] V. Barone, M. Cossi. *J. Phys. Chem. A* **1998**, *102* (11), 1995–2001.
[23] A. V. Marenich, C. J. Cramer, D. G. Truhlar. *J. Phys. Chem. B.* **2009**, *113* (18), 6378–6396.

NMR spectra of new products

Starting materials

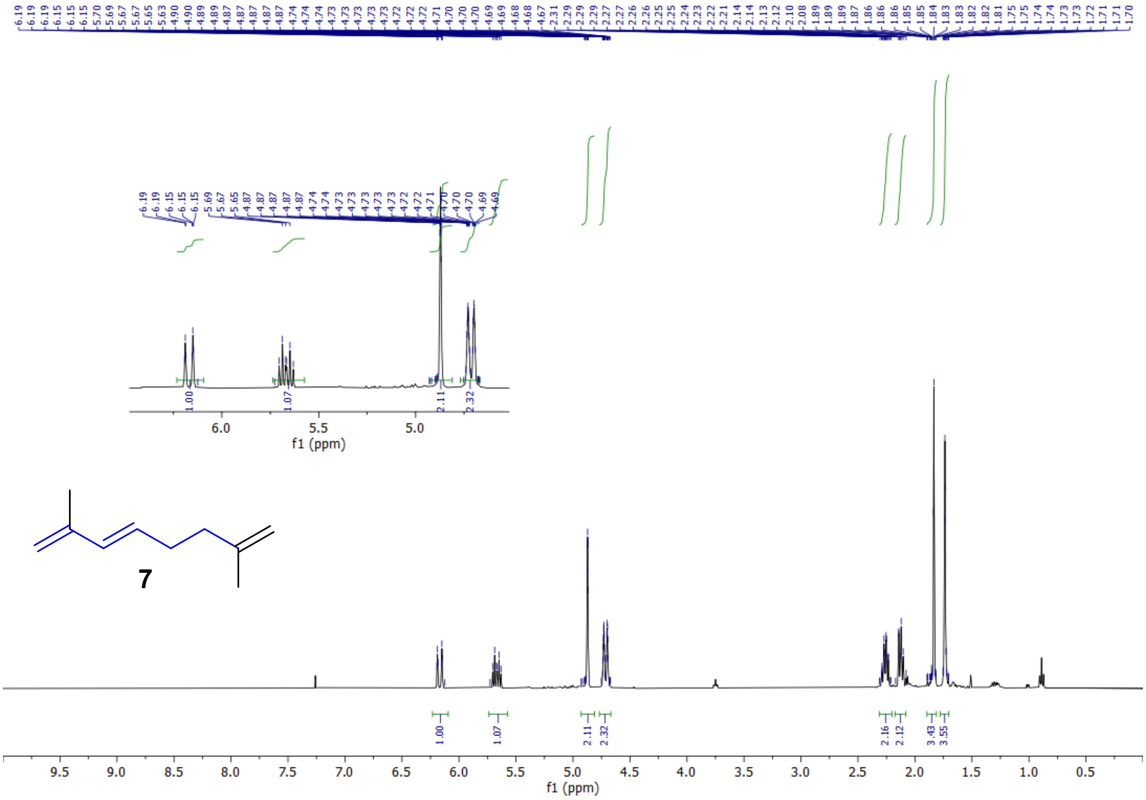

*Figure S55 : ^{1}H NMR spectrum of **7** ($CDCl_3$).*

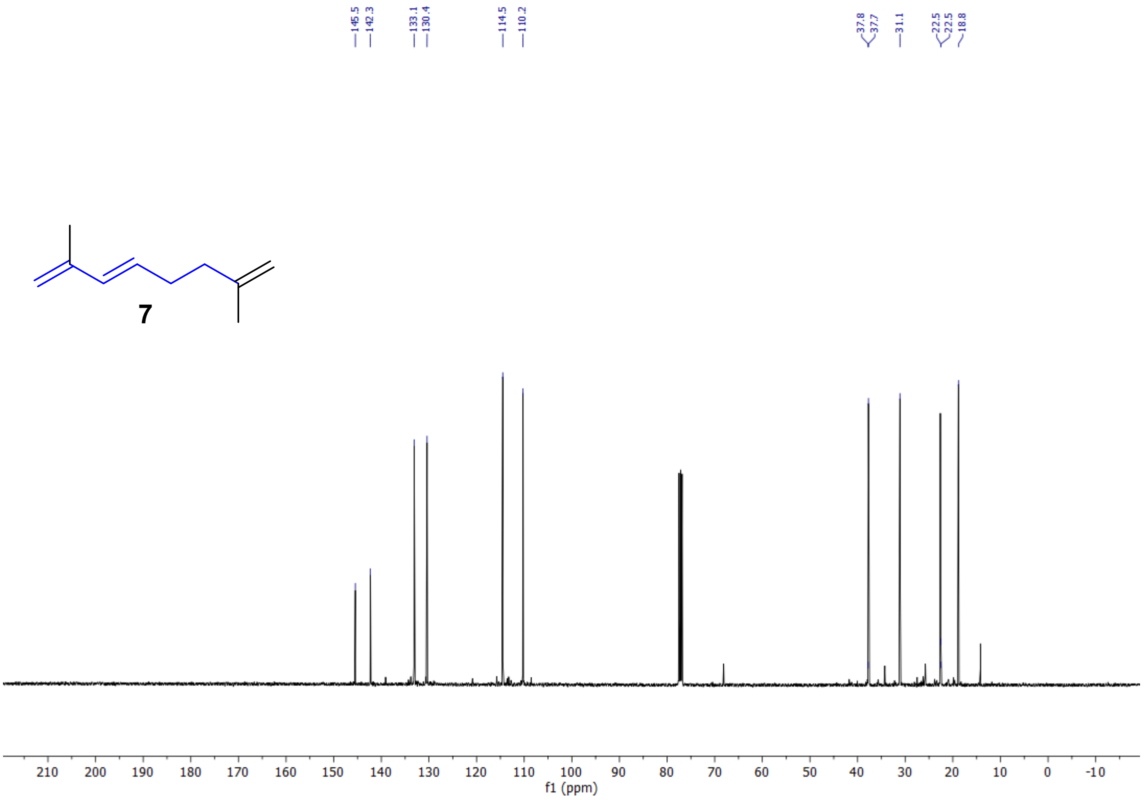

*Figure S56 : ^{13}C NMR spectrum of **7** ($CDCl_3$).*

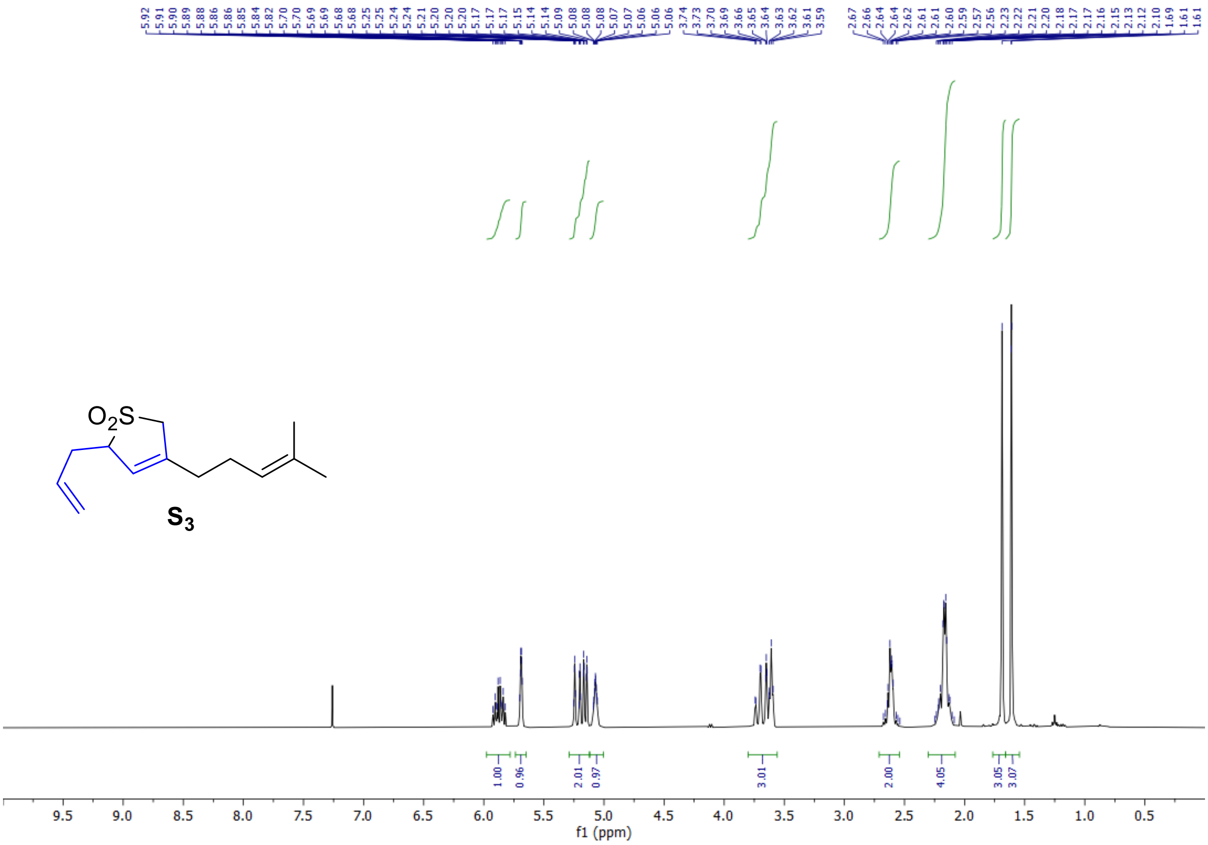

Figure S57 : ^{1}H NMR spectrum of ***S₃*** *(CDCl₃).*

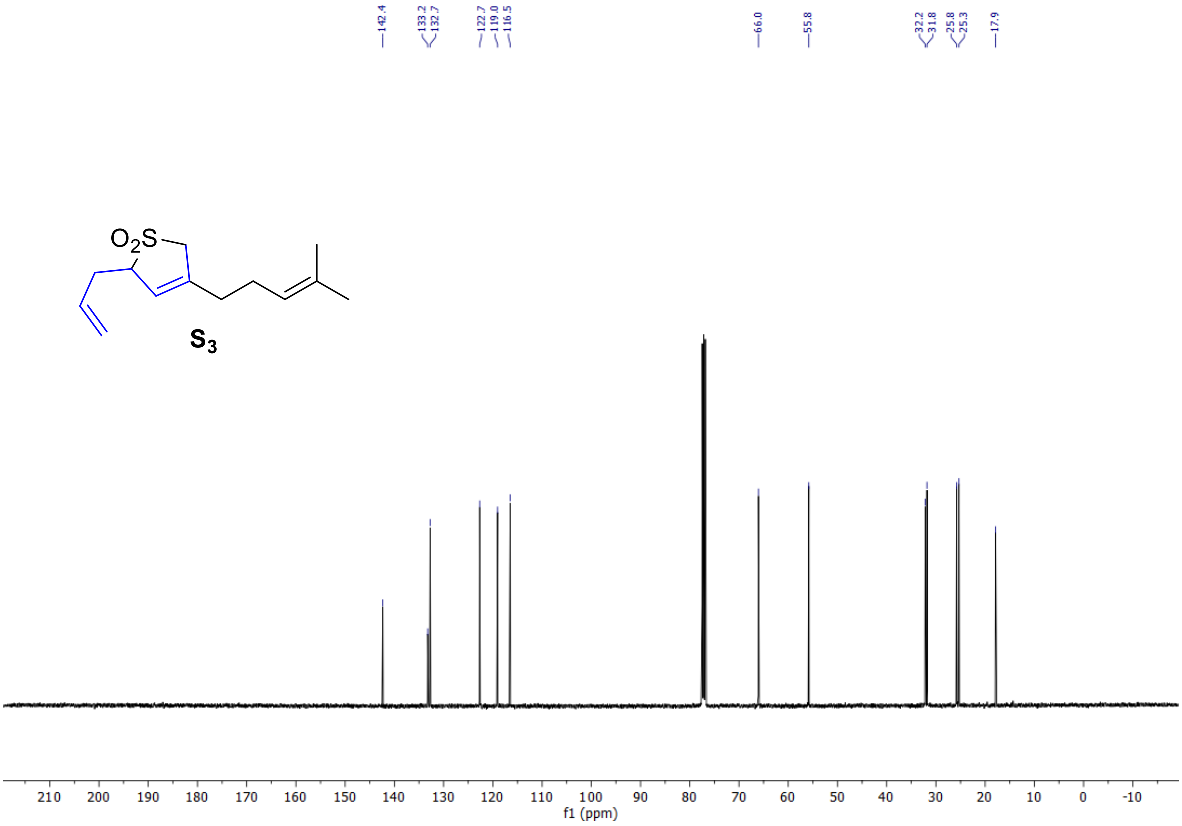

Figure S58 : ^{13}C NMR spectrum of ***S₃*** *(CDCl₃).*

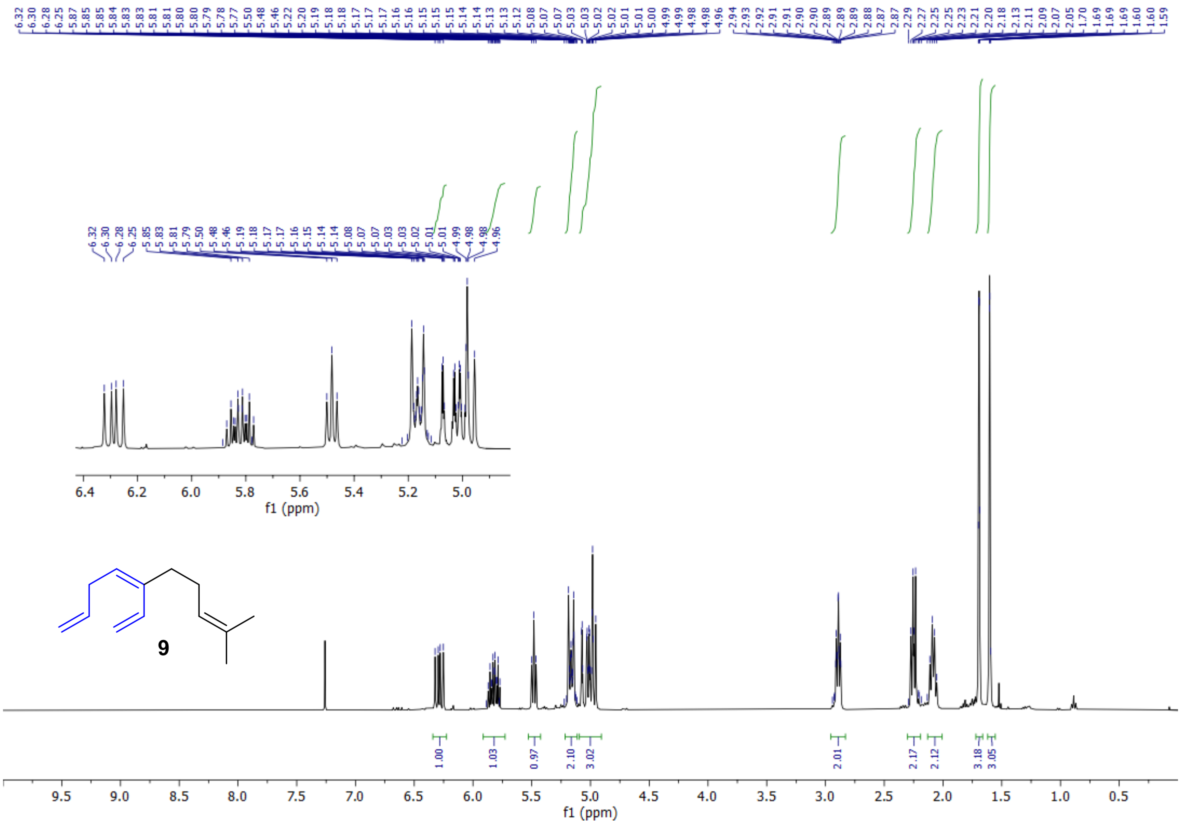

*Figure S59 : ^{1}H NMR spectrum of **9** ($CDCl_3$).*

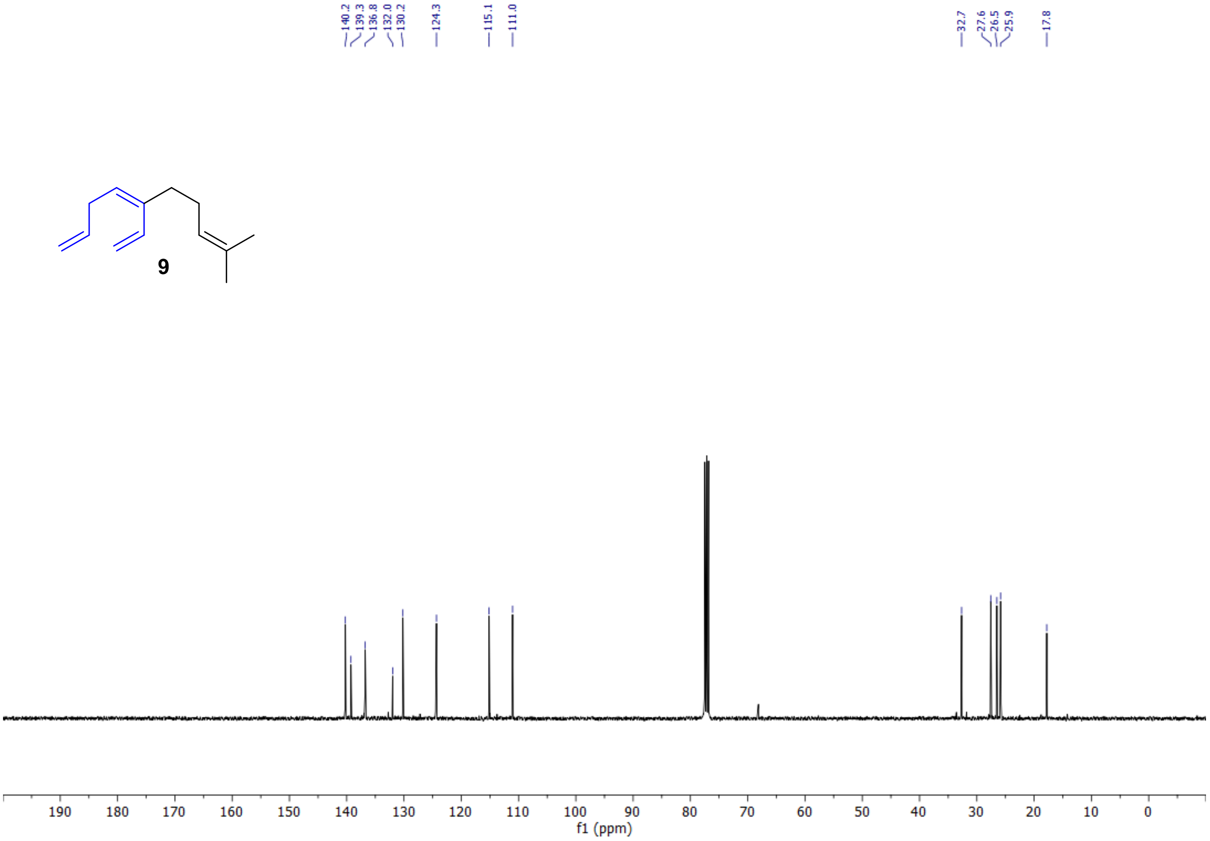

*Figure S60 : ^{13}C NMR spectrum of **9** ($CDCl_3$).*

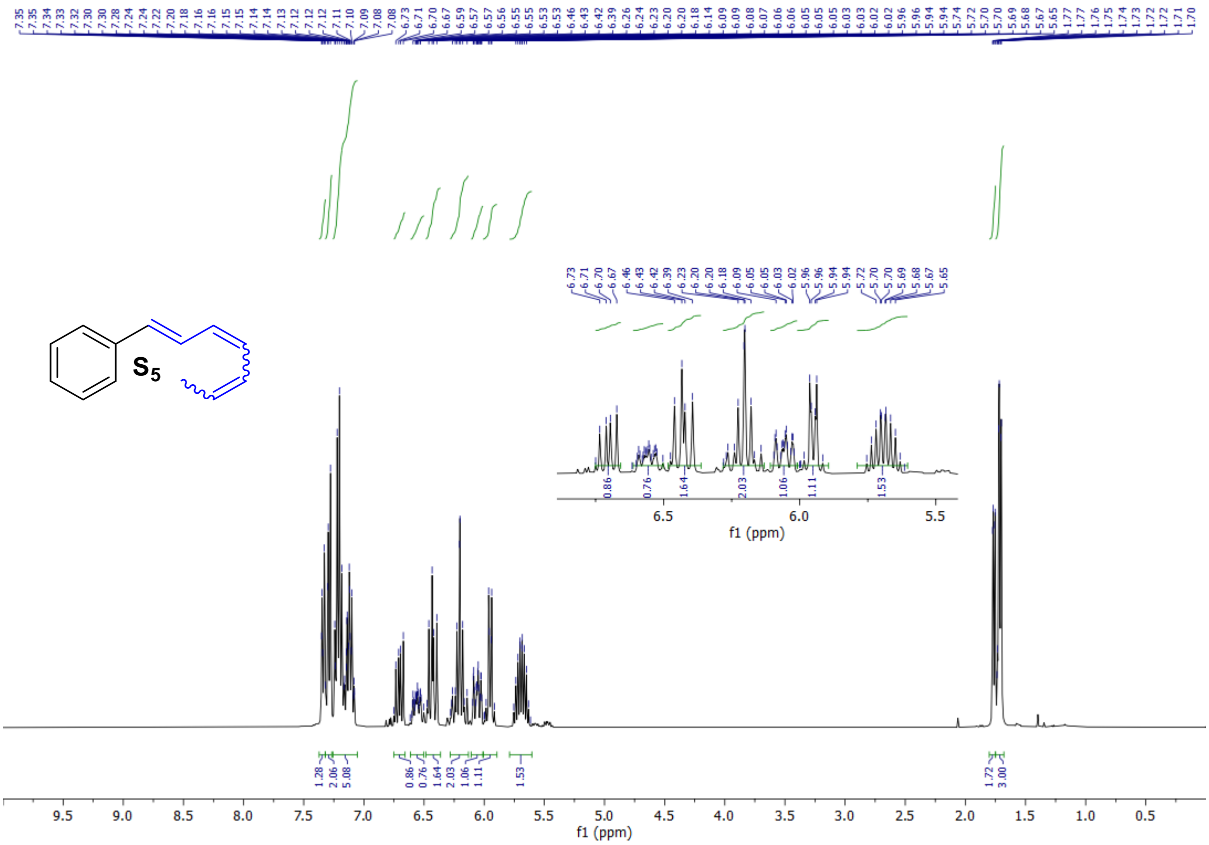

*Figure S61 : ^{1}H NMR spectrum of **S₅** (CDCl₃).*

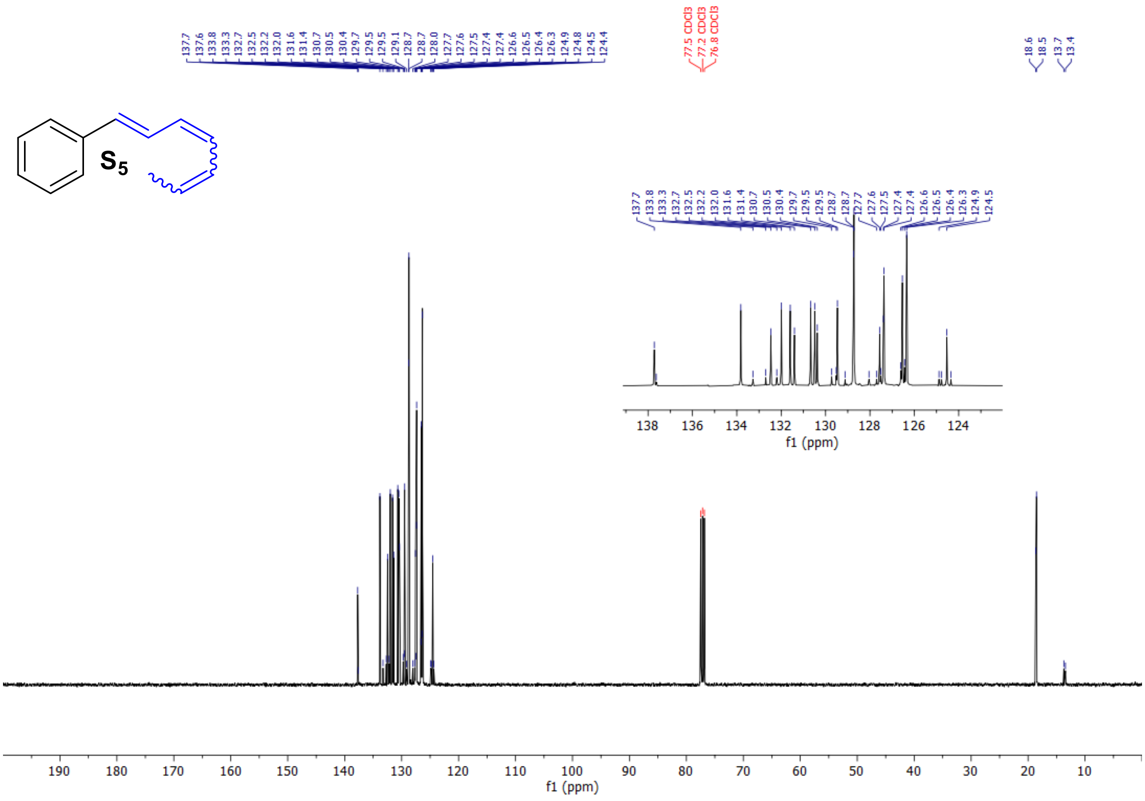

*Figure S62 : ^{13}C NMR spectrum of **S₅** (CDCl₃).*

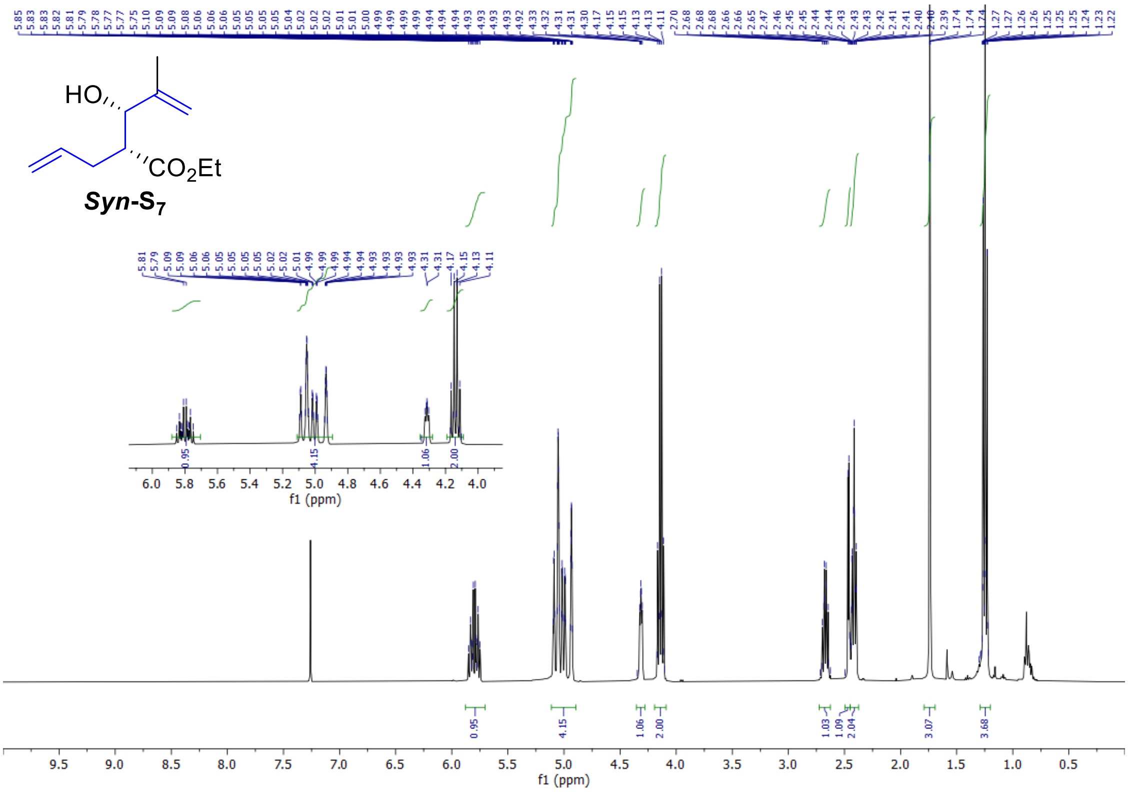

Figure S63 : ^{1}H NMR spectrum of ***Syn*-S$_7$** ($CDCl_3$).

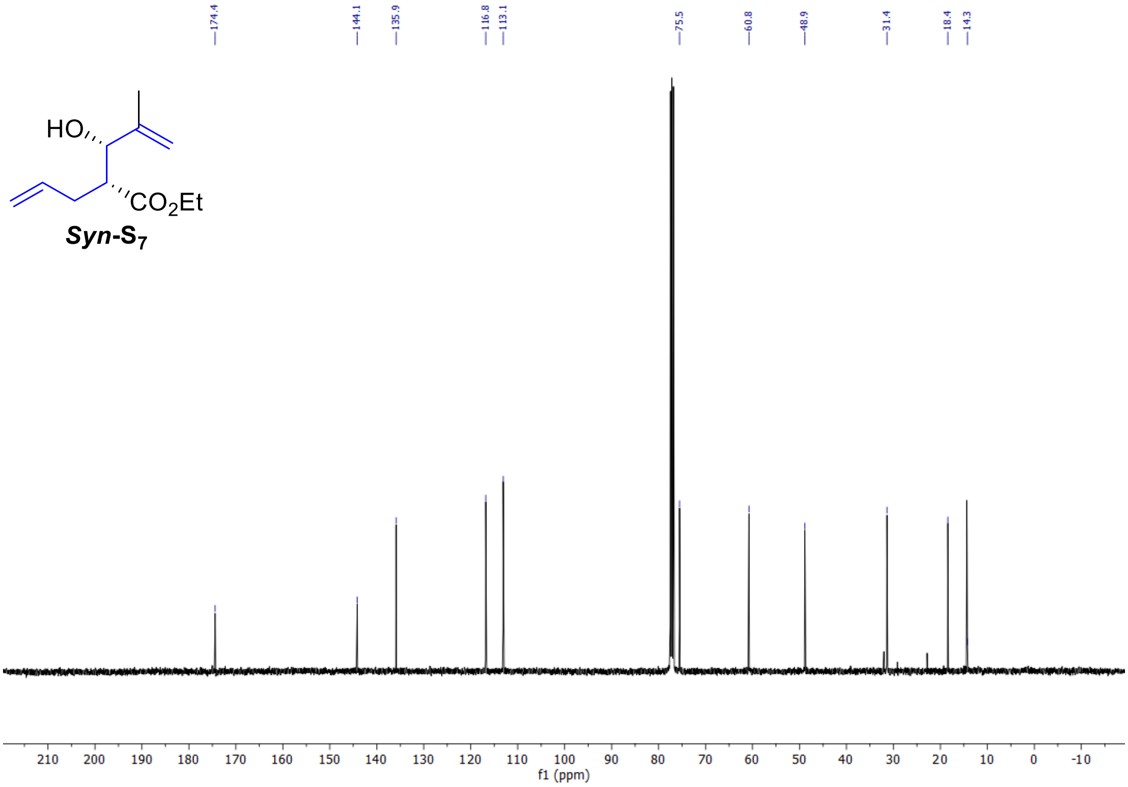

Figure S64 : ^{13}C NMR spectrum of ***Syn*-S$_7$** ($CDCl_3$).

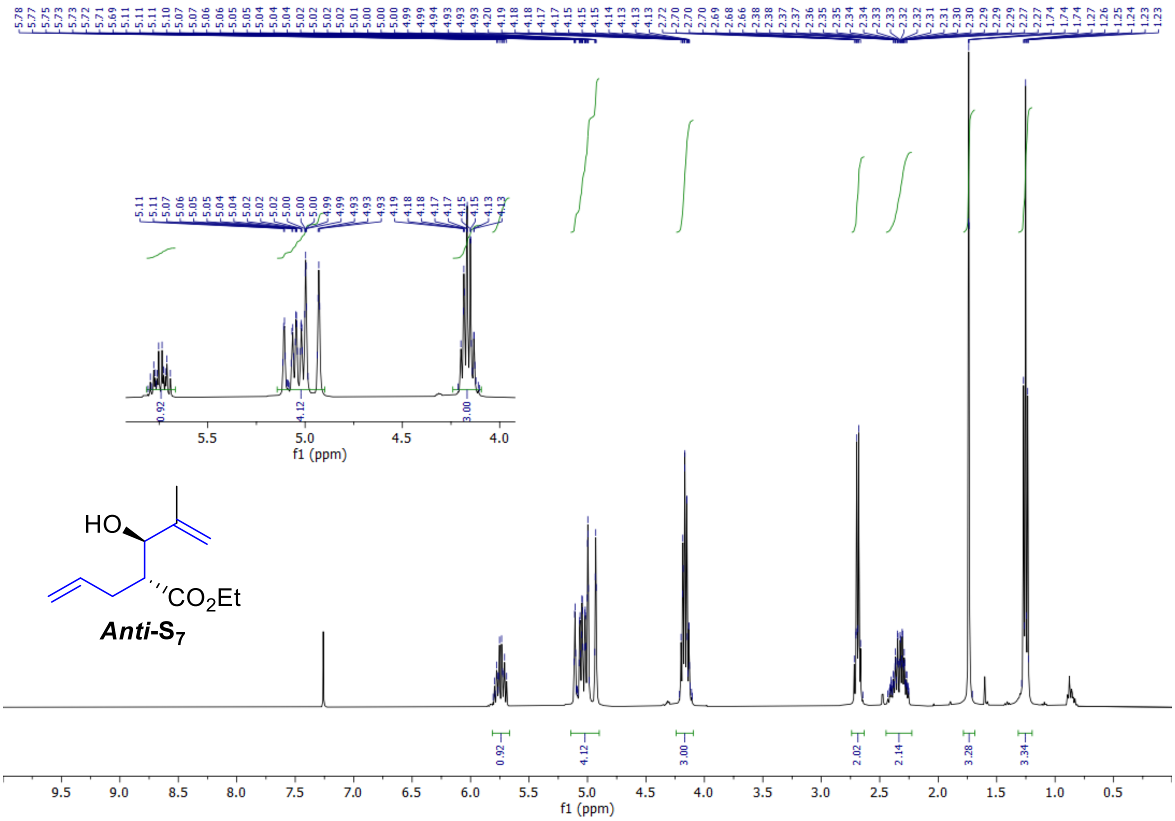

*Figure S65 : ^{1}H NMR spectrum of **Anti-S₇** (CDCl₃).*

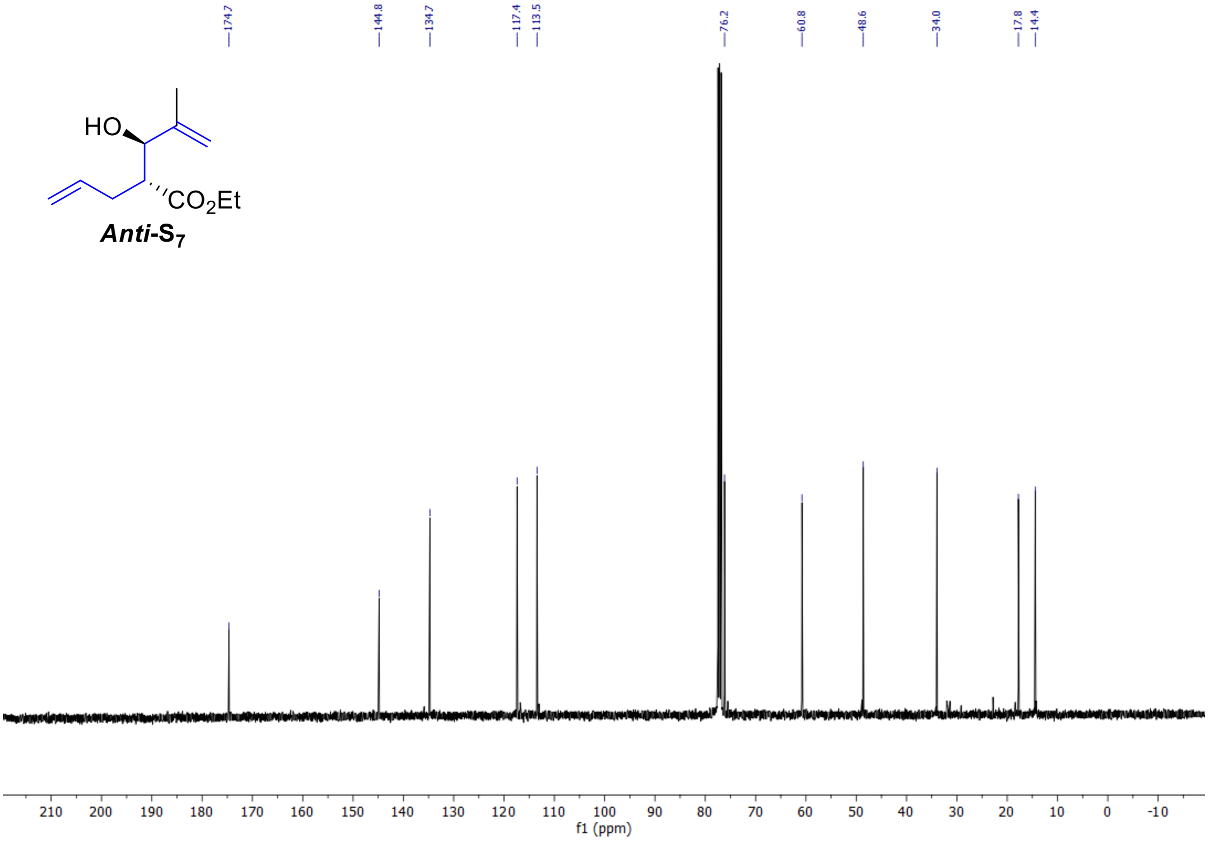

*Figure S66 : ^{13}C NMR spectrum of **Anti-S₇** (CDCl₃).*

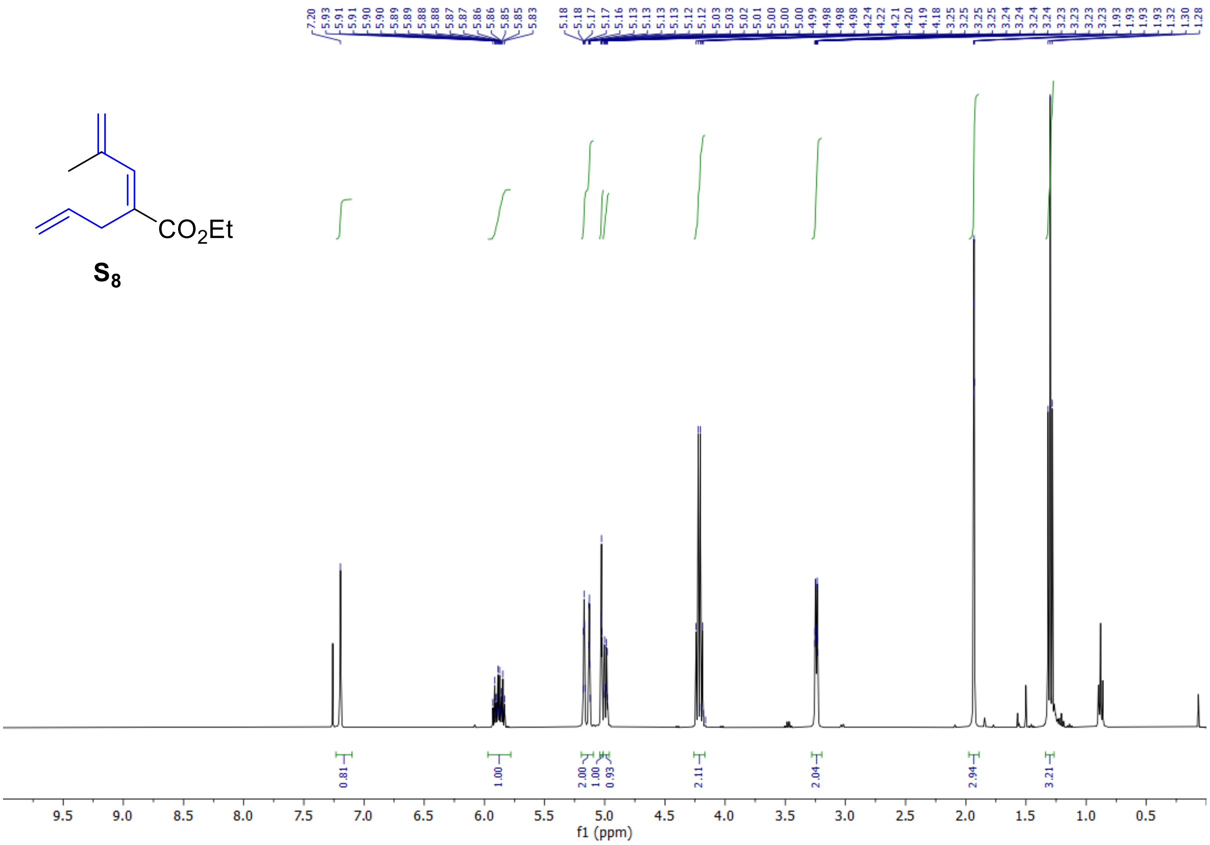

Figure S67 : ^{1}H NMR spectrum of **S₈** (CDCl₃).

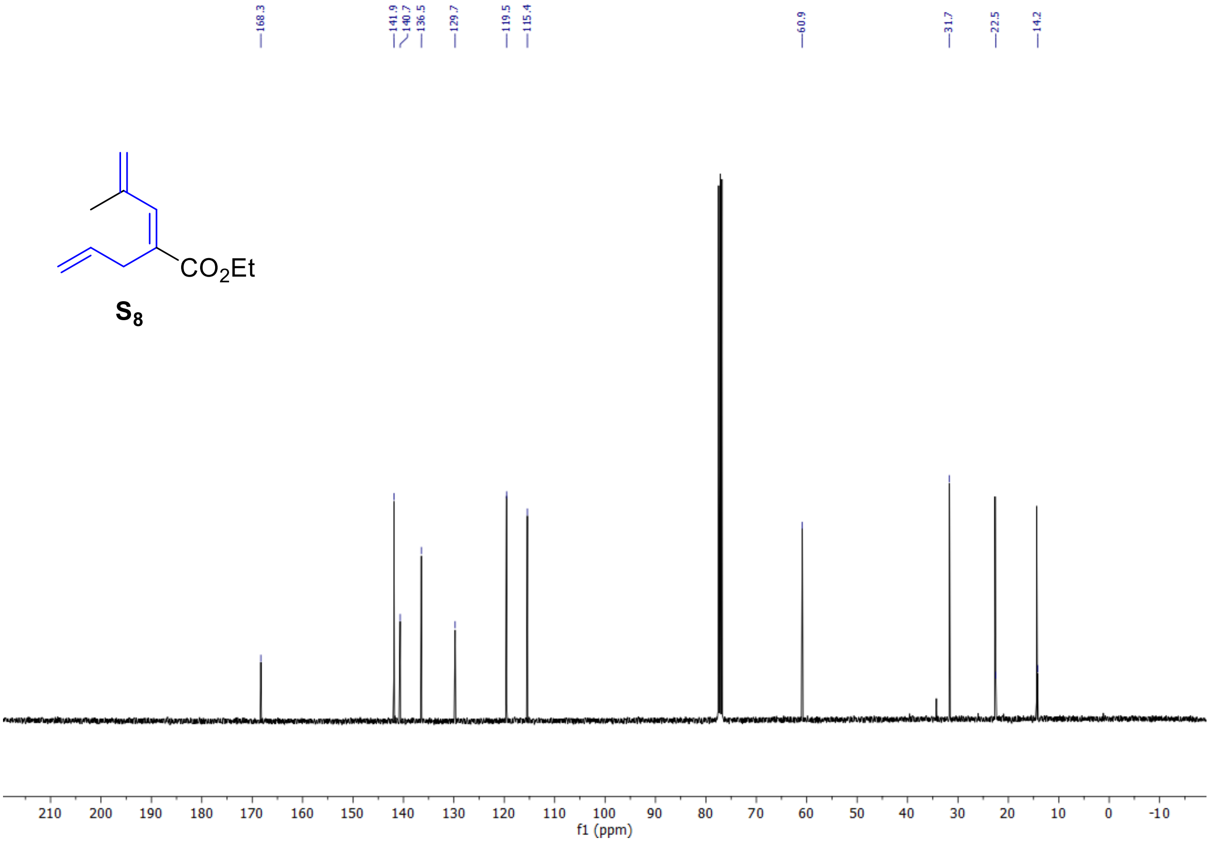

Figure S68 : ^{13}C NMR spectrum of **S₈** (CDCl₃).

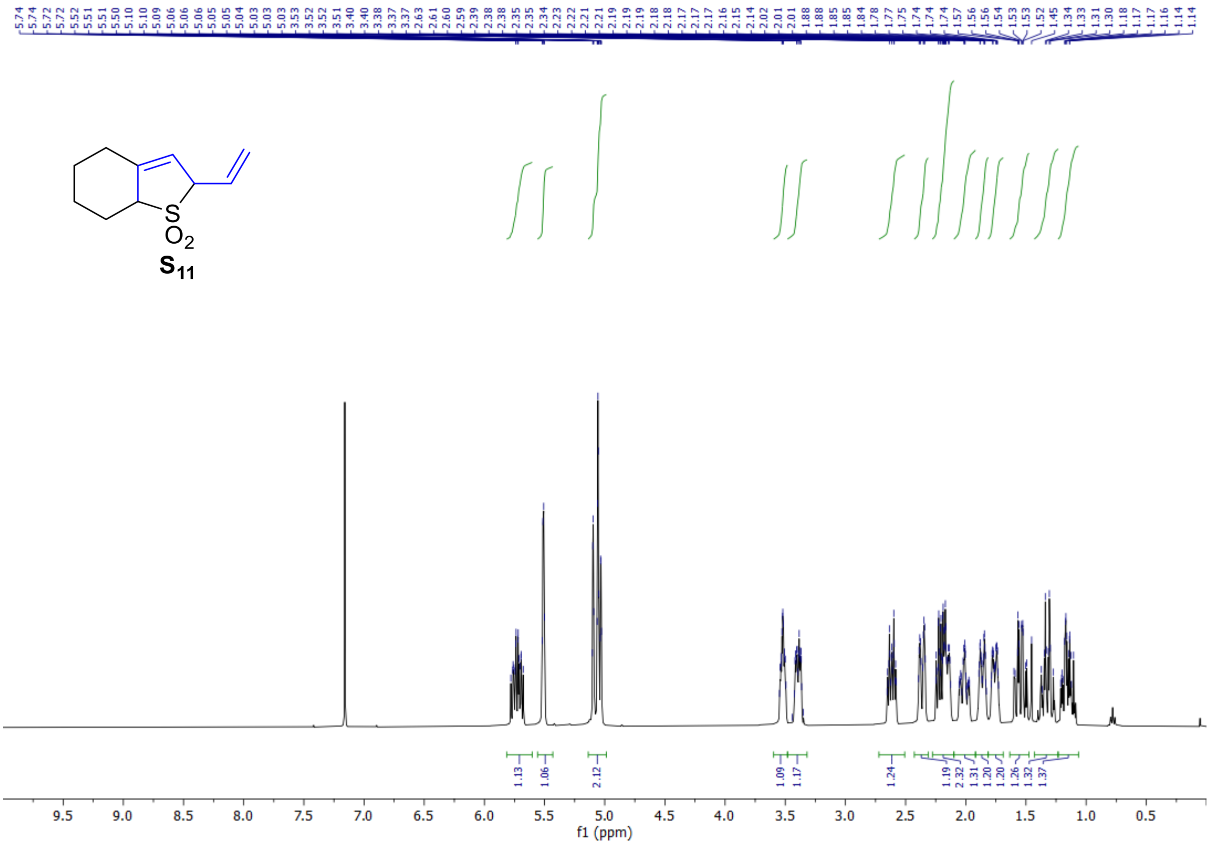

*Figure S69 : ^{1}H NMR spectrum of **S₁₁** (CDCl₃).*

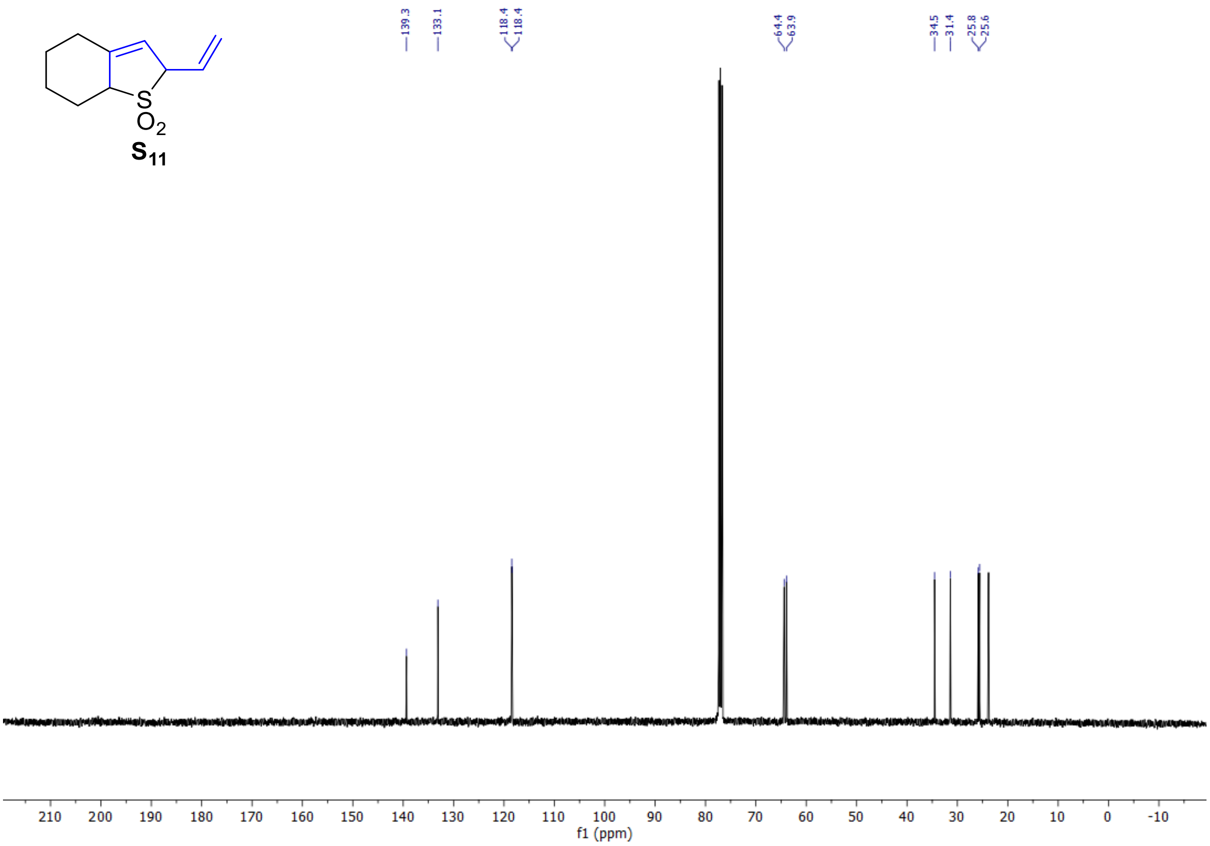

*Figure S70 : ^{13}C NMR spectrum of **S₁₁** (CDCl₃).*

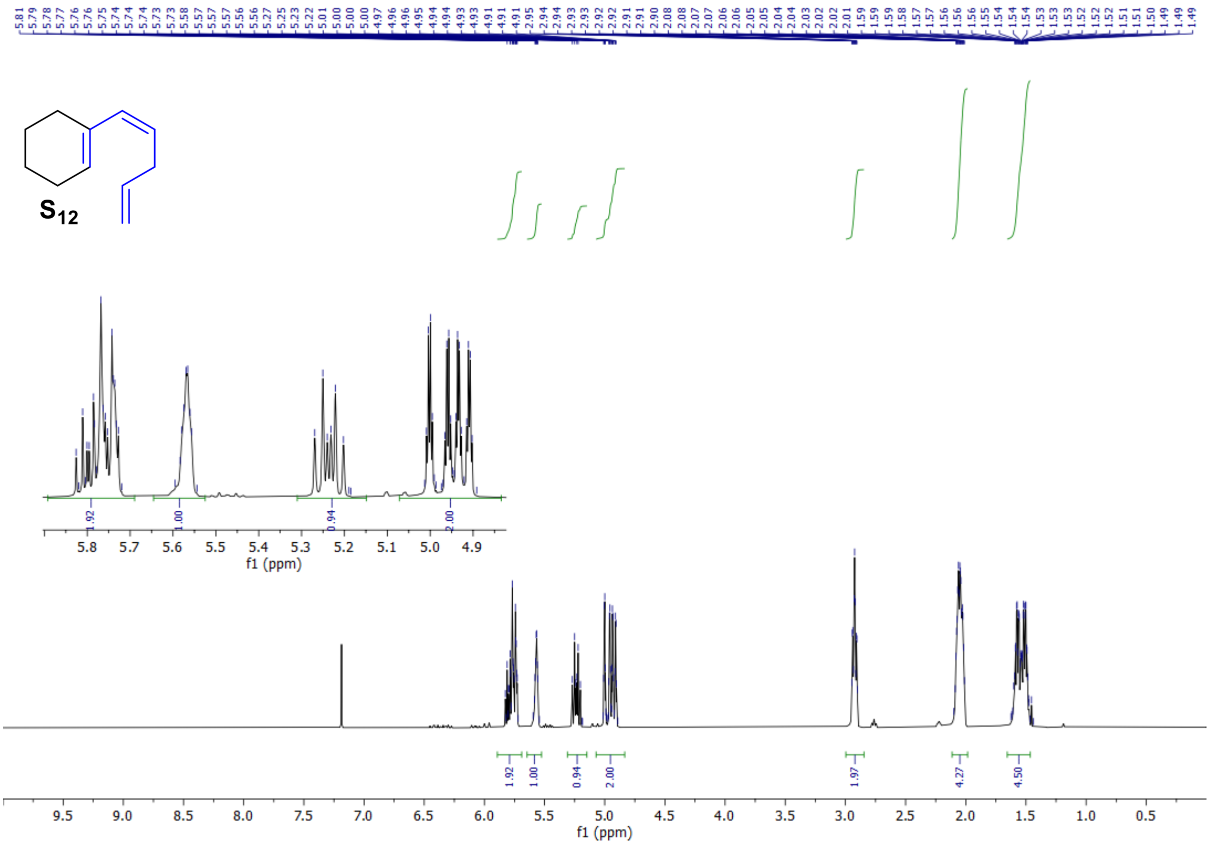

Figure S71 : ^{1}H NMR spectrum of ***S*** $_{12}$ *(CDCl$_3$).*

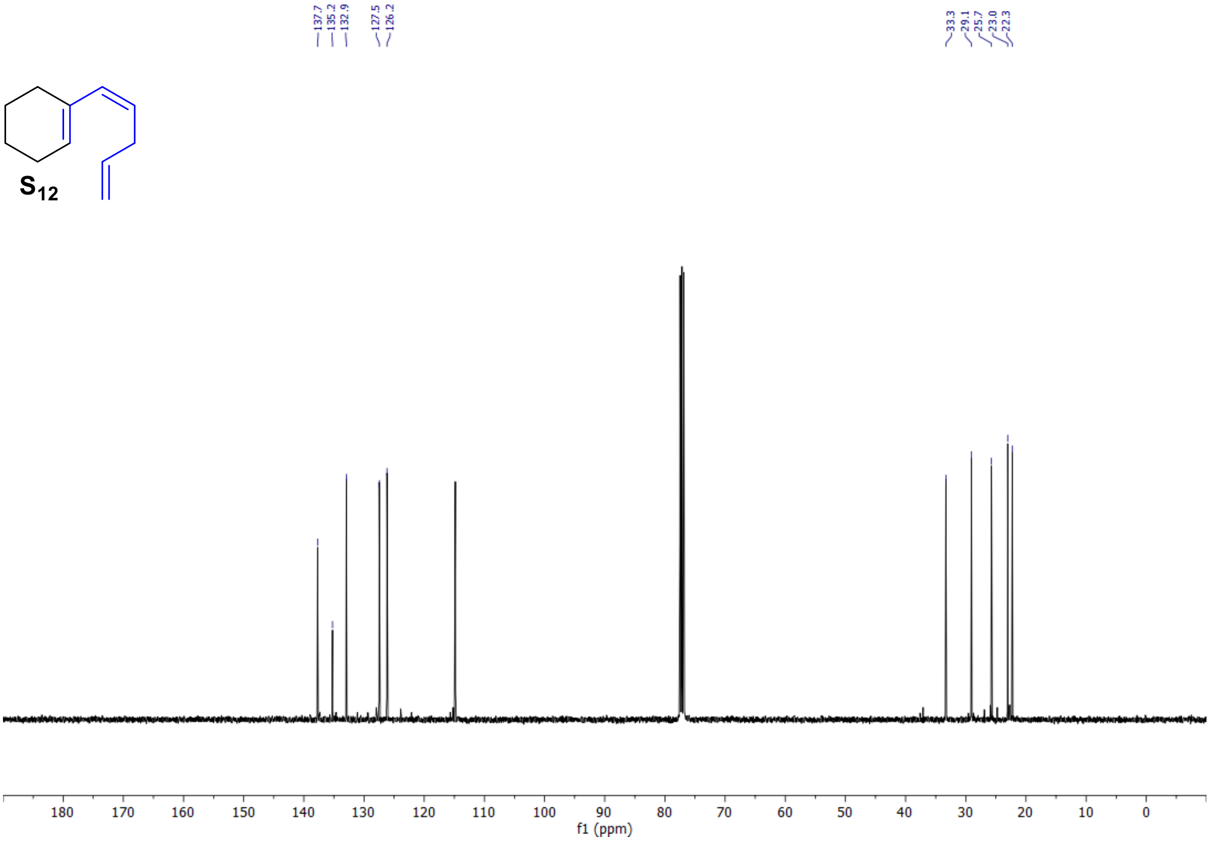

Figure S72 : ^{13}C NMR spectrum of ***S*** $_{12}$ *(CDCl$_3$).*

Cyclized products

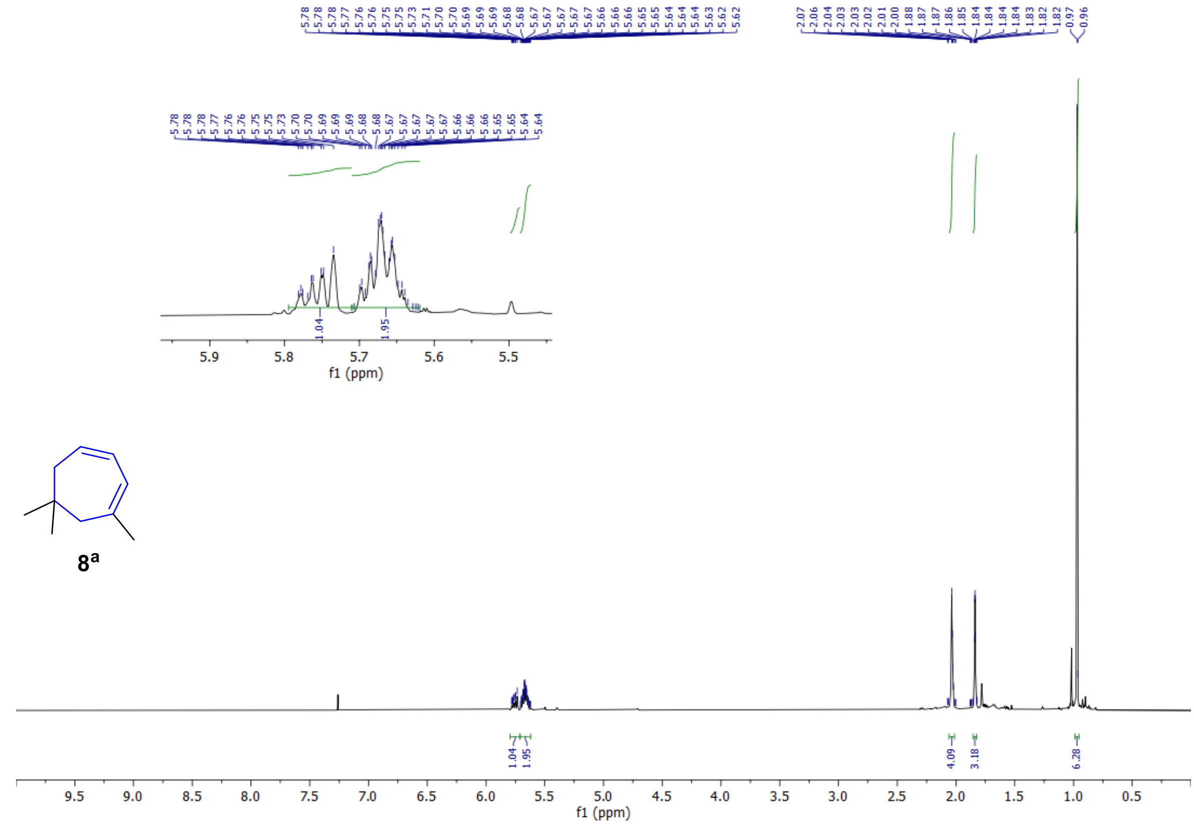

*Figure S73 : ^{1}H NMR spectrum of **8**[a] ($CDCl_3$).*

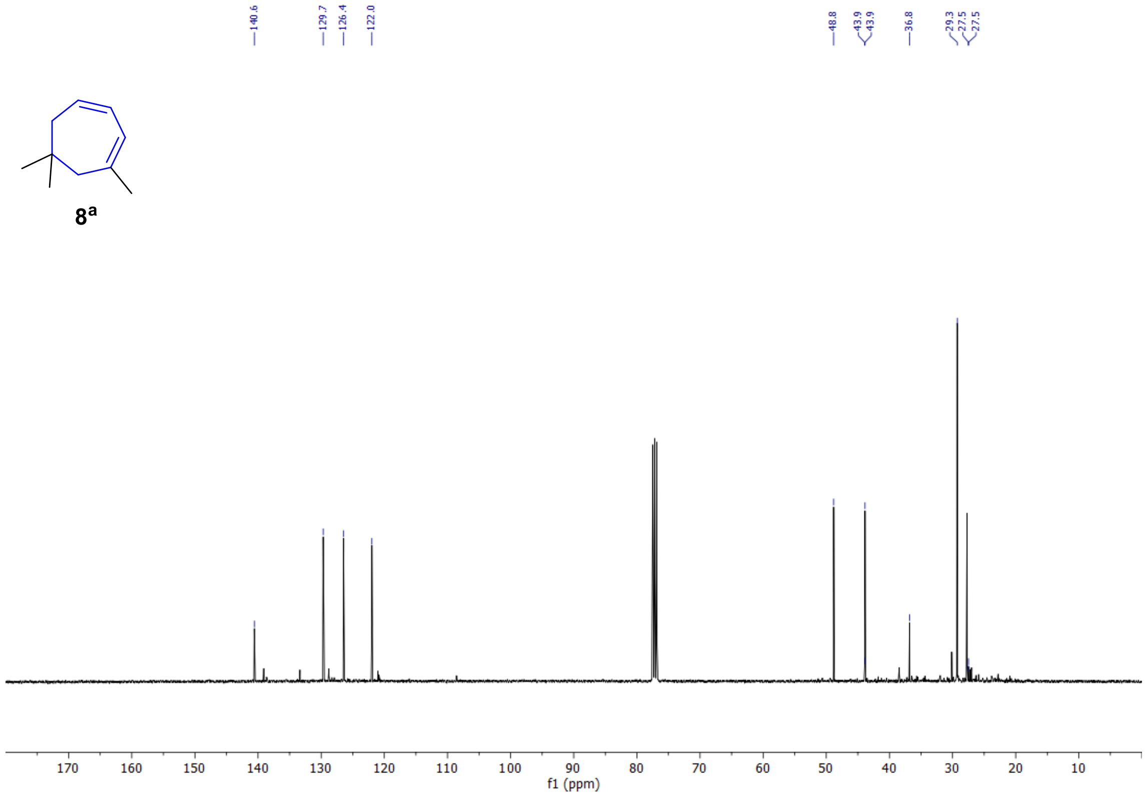

*Figure S74 : ^{13}C NMR spectrum of **8**[a] ($CDCl_3$).*

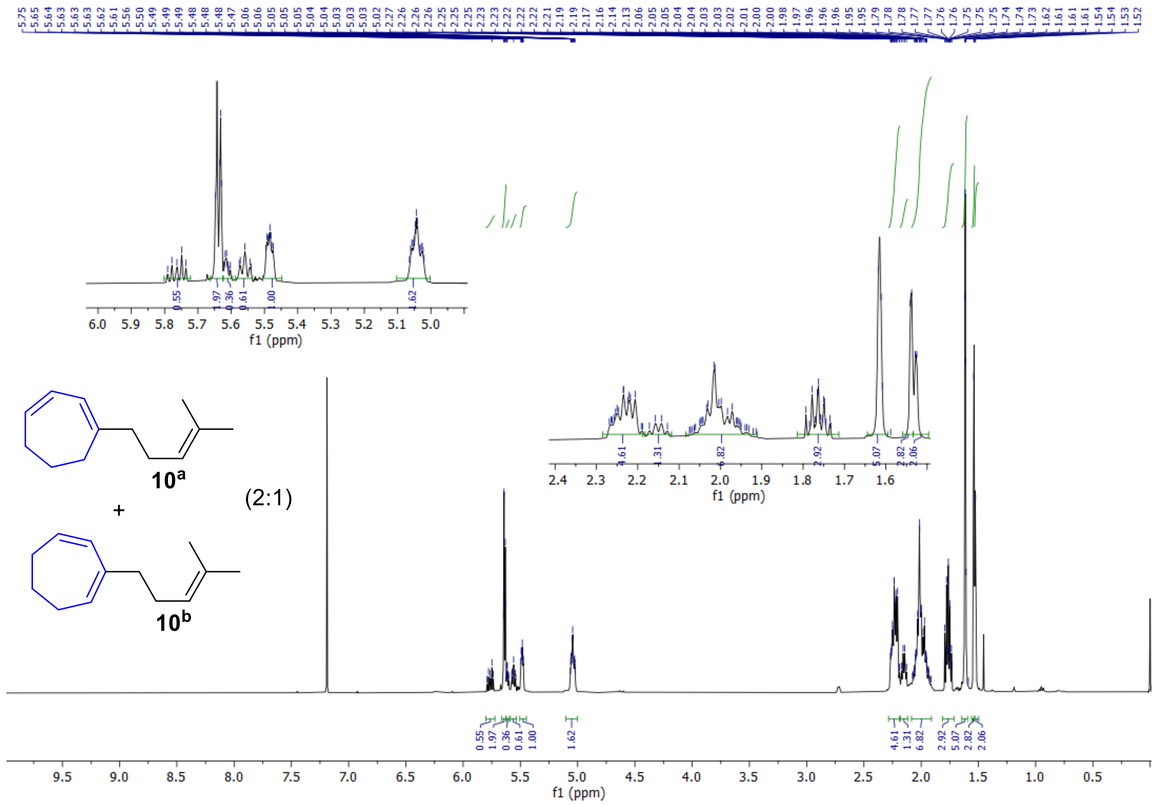

Figure S75 : ^{1}H NMR spectrum of **10**[a-b] mixture (CDCl$_3$).

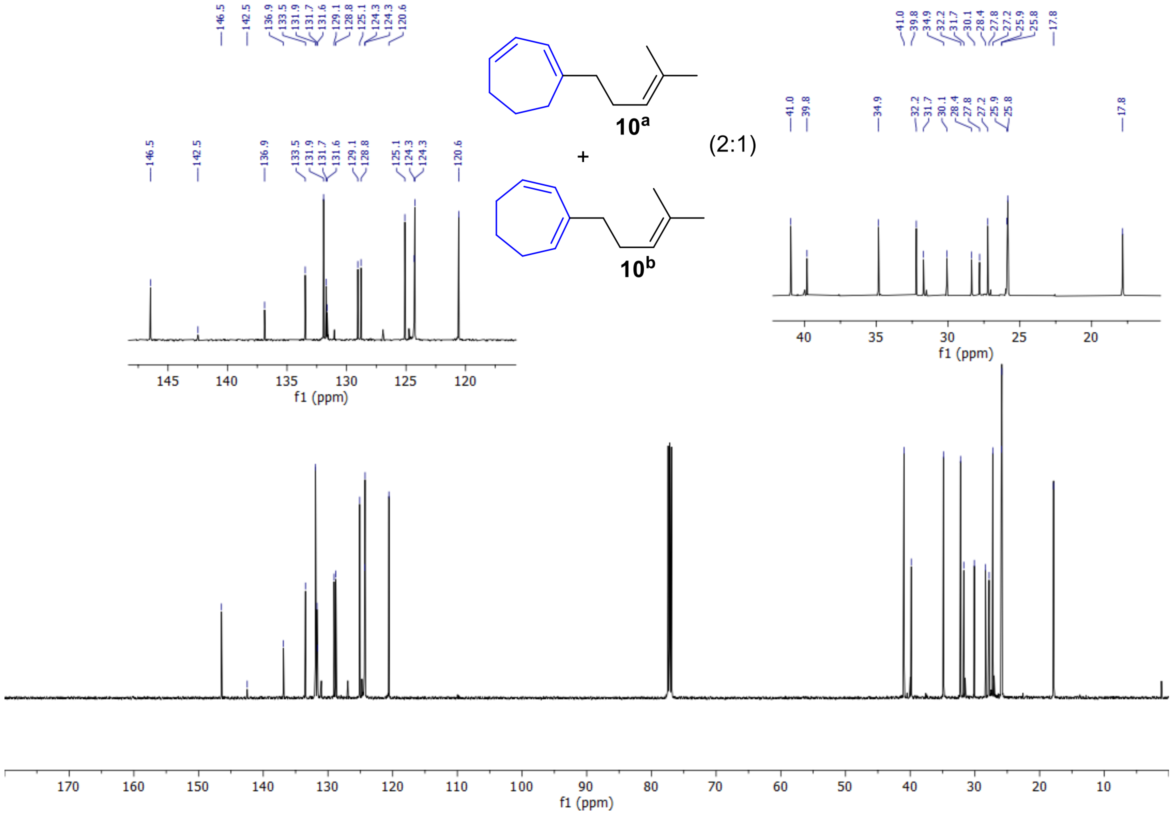

Figure S76 : ^{13}C NMR spectrum of **10**[a-b] mixture (CDCl$_3$).

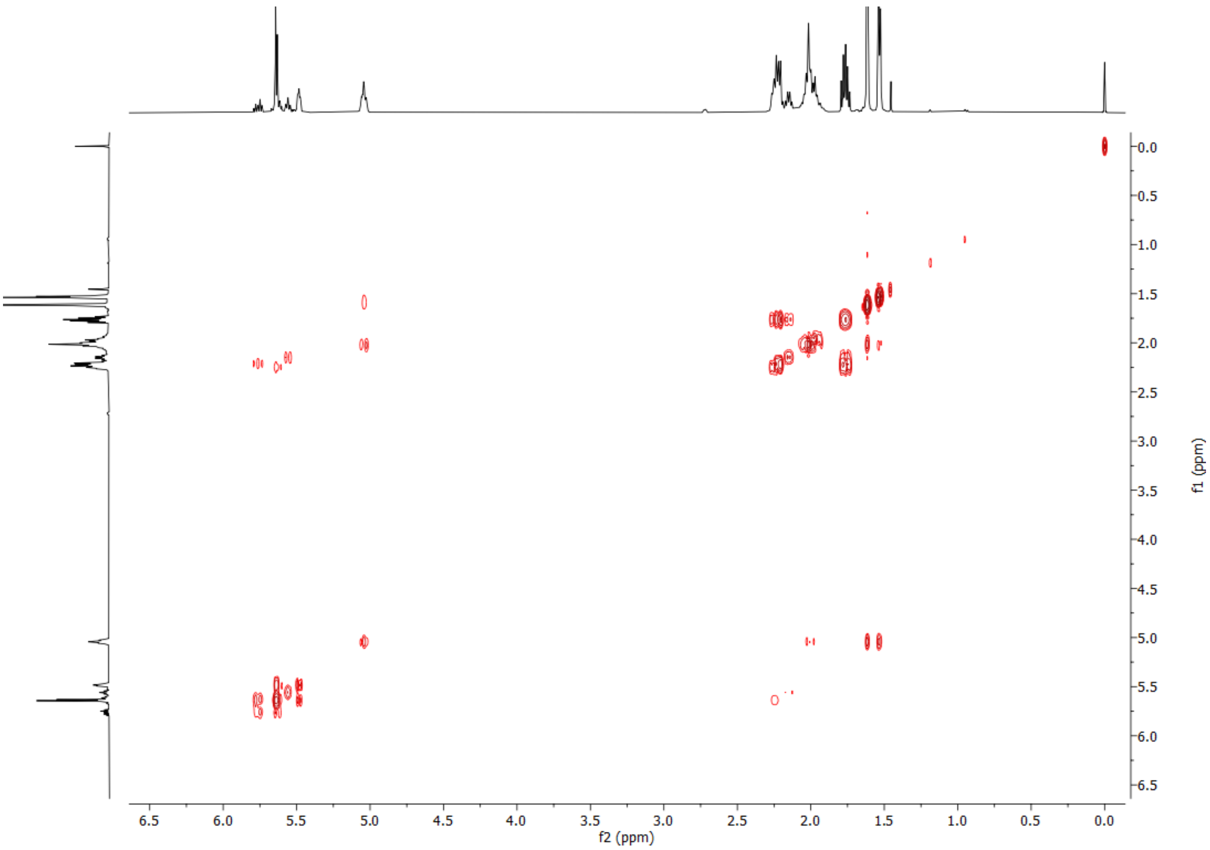

*Figure S77 : COSY spectrum of **10**$^{a\text{-}b}$ mixture (CDCl$_3$).*

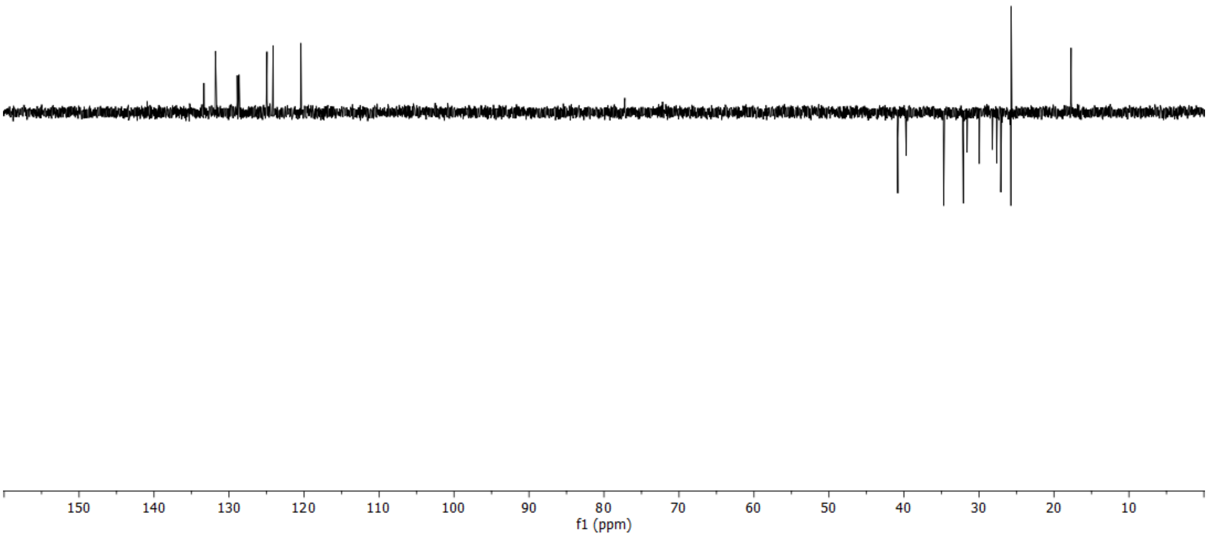

*Figure S78 : DEPT^{135} spectrum of **10**$^{a\text{-}b}$ mixture (CDCl$_3$).*

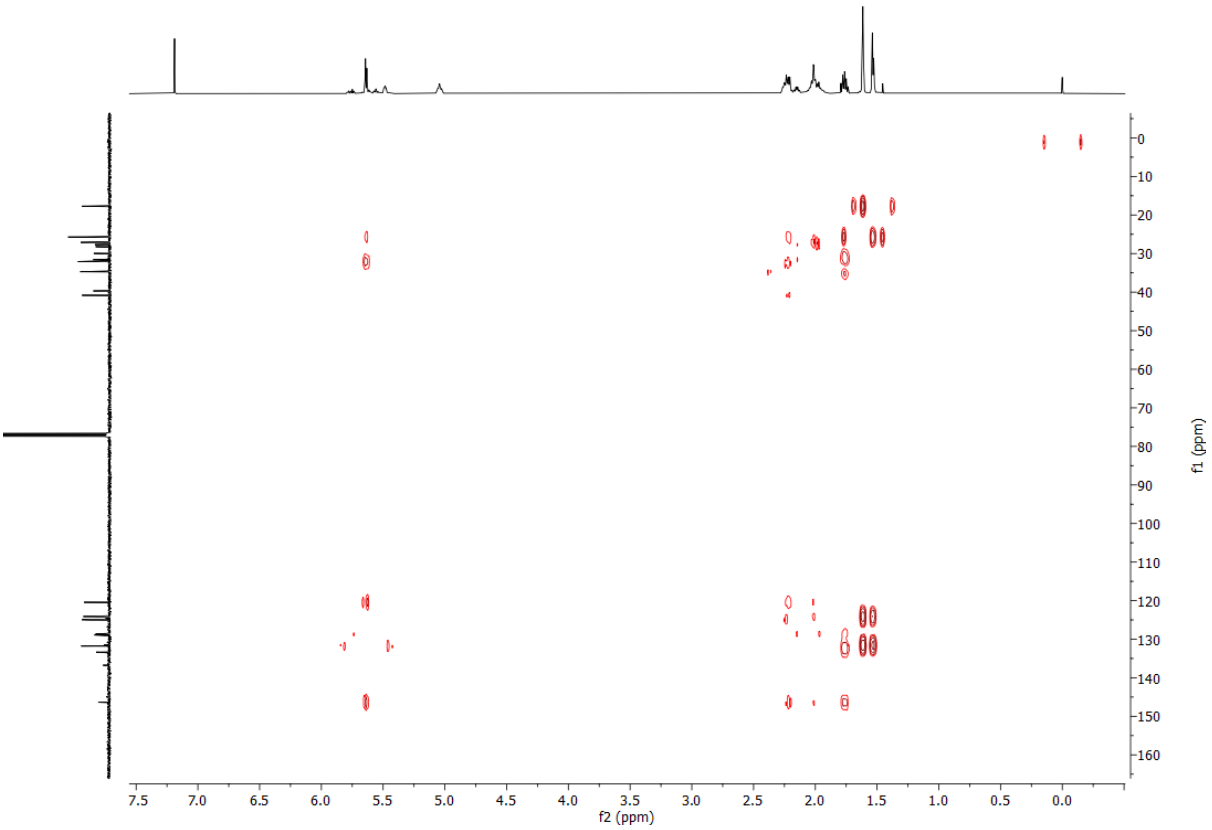

*Figure S79 : HMBC spectrum of **10**^a-b^ mixture (CDCl$_3$).*

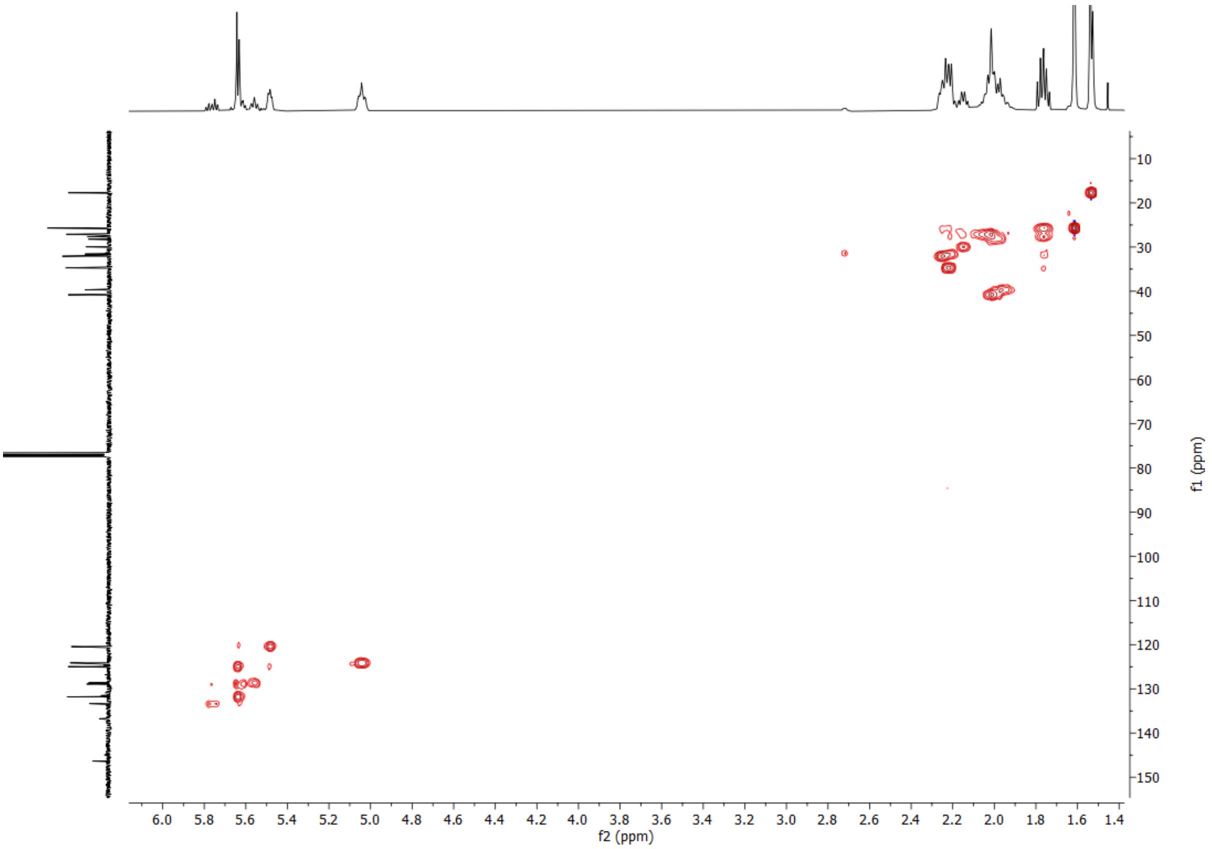

*Figure S80 : HSQC spectrum of **10**^a-b^ mixture (CDCl$_3$).*
